# DNA Strand Displacement with Base Pair Stabilizers: Purine‐2,6‐Diamine and 8‐Aza‐7‐Bromo‐7‐Deazapurine‐2,6‐Diamine Oligonucleotides Invade Canonical DNA and New Fluorescent Pyrene Click Sensors Monitor the Reaction

**DOI:** 10.1002/chem.202202412

**Published:** 2022-11-15

**Authors:** Aigui Zhang, Dasharath Kondhare, Peter Leonard, Frank Seela

**Affiliations:** ^1^ Laboratory of Bioorganic Chemistry and Chemical Biology Center for Nanotechnology Heisenbergstrasse 11 48149 Münster Germany; ^2^ Laboratorium für Organische und Bioorganische Chemie Institut für Chemie neuer Materialien Universität Osnabrück Barbarastrasse 7 49069 Osnabrück Germany

**Keywords:** base pair stabilizer, hybridization, oligonucleotides, pyrene fluorescence, strand displacement

## Abstract

Purine‐2,6‐diamine and 8‐aza‐7‐deaza‐7‐bromopurine‐2,6‐diamine 2’‐deoxyribonucleosides (**1** and **2**) were implemented in isothermal DNA strand displacement reactions. Nucleoside **1** is a weak stabilizer of dA‐dT base pairs, nucleoside **2** evokes strong stabilization. Strand displacement reactions used single‐stranded invaders with single and multiple incorporations of stabilizers. Displacement is driven by negative enthalpy changes between target and displaced duplex. Toeholds are not required. Two new environmental sensitive fluorescent pyrene sensors were developed to monitor the progress of displacement reactions. Pyrene was connected to the nucleobase in the invader or to a dendritic linker in the output strand. Both new sensors were constructed by click chemistry; phosphoramidites and oligonucleotides were prepared. Sensors show monomer or excimer emission. Fluorescence intensity changes when the displacement reaction progresses. Our work demonstrates that strand displacement with base pair stabilizers is applicable to DNA, RNA and to related biopolymers with applications in chemical biology, nanotechnology and medicinal diagnostics.

## Graphical abstract

**Base pair stabilizers 1** and **2** were used to drive isothermal displacement reactions. Fluorescent pyrene sensors were developed to monitor the reaction progress by fluorescence change.

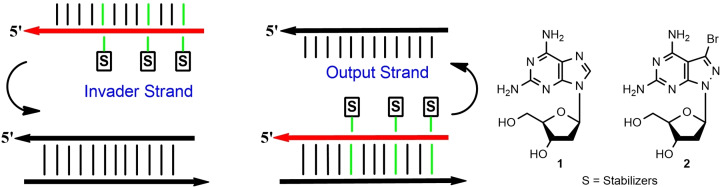

## Introduction

DNA strand displacement is a process in which one strand of duplex DNA is replaced by an invader strand, thereby releasing an output strand from the DNA double helix. The reaction takes place at constant temperature, is thermodynamically driven, and controlled by a negative ΔG value. Displacement occurs in many biological contexts and technological applications; in gene regulation, dynamic DNA nanotechnology, biosensing, and has led to the construction of DNA motors.^[1]^ The heart of strand displacement is hybridization and the stronger binding of the invader strand to the target duplex compared to the released strand. Additional base pairs, so‐called toeholds were implemented to stabilize the invader complex.^[2]^ Another option to strengthen the binding of the invader strand to duplex DNA is the use of modified nucleosides in place of canonical nucleosides evoking higher base pair stability.^[3]^ This process does not require toeholds. Toehold‐free displacement was recently reported for anomeric DNA.^[3d]^ In this protocol, different stabilities of heterochiral vs. homochiral DNA were applied.

In the realm of DNA, a number of modified nucleosides have been reported that stabilize Watson‐Crick base pairs.^[4]^ Some of them are naturally occurring; most are of synthetic origin. Nucleobases, sugar moieties, exocyclic substituents and the phosphate residue are common targets for functionalization. Chemistry, biology, medicinal diagnostics and therapeutics and more recently nanotechnology make use of this phenomenon.^[5]^ Our laboratory has reported a series of compounds that are in common use for DNA detection, sensing, sequencing and the construction of entirely new DNAs.[^4b^, ^4c^, ^6^]

**Figure 1:**
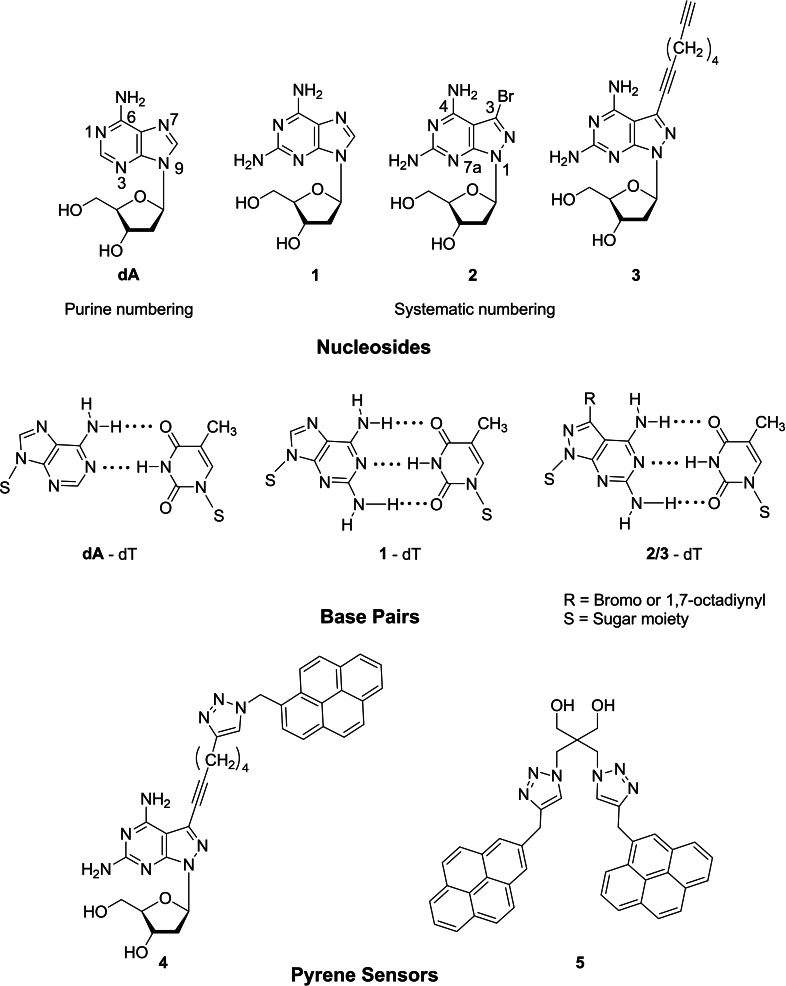
Up: Modified nucleoside stabilizers **1–3** incorporated in the invader strand for the displacement reaction. Middle: Bidentate and tridentate base pairs. Low: Pyrene sensors **4** and **5** used to monitor the progress of displacement reactions.

The purine‐2,6‐diamine nucleoside **1** ^[7]^ is a constituent of the Cyanophage S‐2 DNA and replaces dA in the virus genome to 100 % (Figure 1).^[7a]^ This nucleoside is a weak DNA stabilizer when it substitutes dA in the dA‐dT base pair (purine numbering is used throughout the results and discussion section and systematic numbering in the Experimental Section, Figure 1).^[8]^ With respect to dA, compound **1** contains an additional amino group at the 2‐position of the nucleobase. Related nucleosides such as 2‐amino‐7‐deazaadenine and 2‐amino‐8‐aza‐7‐deazaadenine 2’‐deoxyribonucleosides show similar properties.[^4b^, ^9^] Contrary to **1**, the 8‐aza‐7‐bromo‐7‐deazapurine‐2,6‐diamine 2’‐deoxyribonucleoside **2** is a strong DNA stabilizer.[^4b^, ^10^] Here, the purine ring is replaced by a pyrazolo[3,4‐*d*]pyrimidine skeleton and the heterocycle carries the amino group at the 2‐position as in **1**. The presence of an additional bromo atom at the 7‐position enhances base pair stability (Figure 1). According to this, nucleoside **2** is more effective in duplex stabilization than nucleoside **1**. Furthermore, compound **2** possesses a more stable glycosylic bond compared to the labile purine nucleoside **1**.^[10a]^ In this work, we use the stabilizing forces of **1** and **2** to drive DNA strand displacement. Toeholds are not required.

For strand displacements reactions, sensors have to be implemented that monitor the progress of the displacement reaction and provide information on their kinetics. For this, FRET quencher systems are commonly applied.^[3d]^ Recently, our laboratory used the fluorescence of ethidium bromide fluorescence as external sensor. For this work, the two new environmental sensitive fluorescence pyrene sensors **4** and **5** were developed and were implemented in the displacement systems.

## Results and Discussion

### Design of strand displacement systems A and B and function of base pair stabilizers and sensors

Stabilization of DNA is beneficial for many applications in DNA research. For the purpose, in the toehold‐assisted displacement systems additional base pairs are implemented strengthening the binding capacity of the invader strand.[^2^, ^3^] In the systems described in this work, existing base pairs are stabilized by base pair stabilizers. Stabilizer **1** is a naturally occurring nucleoside and has a long history in DNA research.[^7^, ^11^] Stabilizer **2** is of synthetic origin. To provide the option for labelling, the clickable linker nucleoside **3** was designed. Nucleoside **2** found widespread application as efficient DNA stabilizer without disturbing the DNA double helical structure.[^8h^, ^10^] The stabilizing properties of **1** and **2** were used to strengthen homochiral and heterochiral DNA and to harmonize the stability of the dA‐dT and dG‐dC base pair.[^8h^, ^10^] The stabilizers **1** and **2** have been already incorporated in DNA by solid‐phase synthesis employing phosphoramidite chemistry.[^8h^, ^10^, ^12^] The phosphoramidite building block of **3** with a clickable linker is described in this work.

**Scheme 1:**
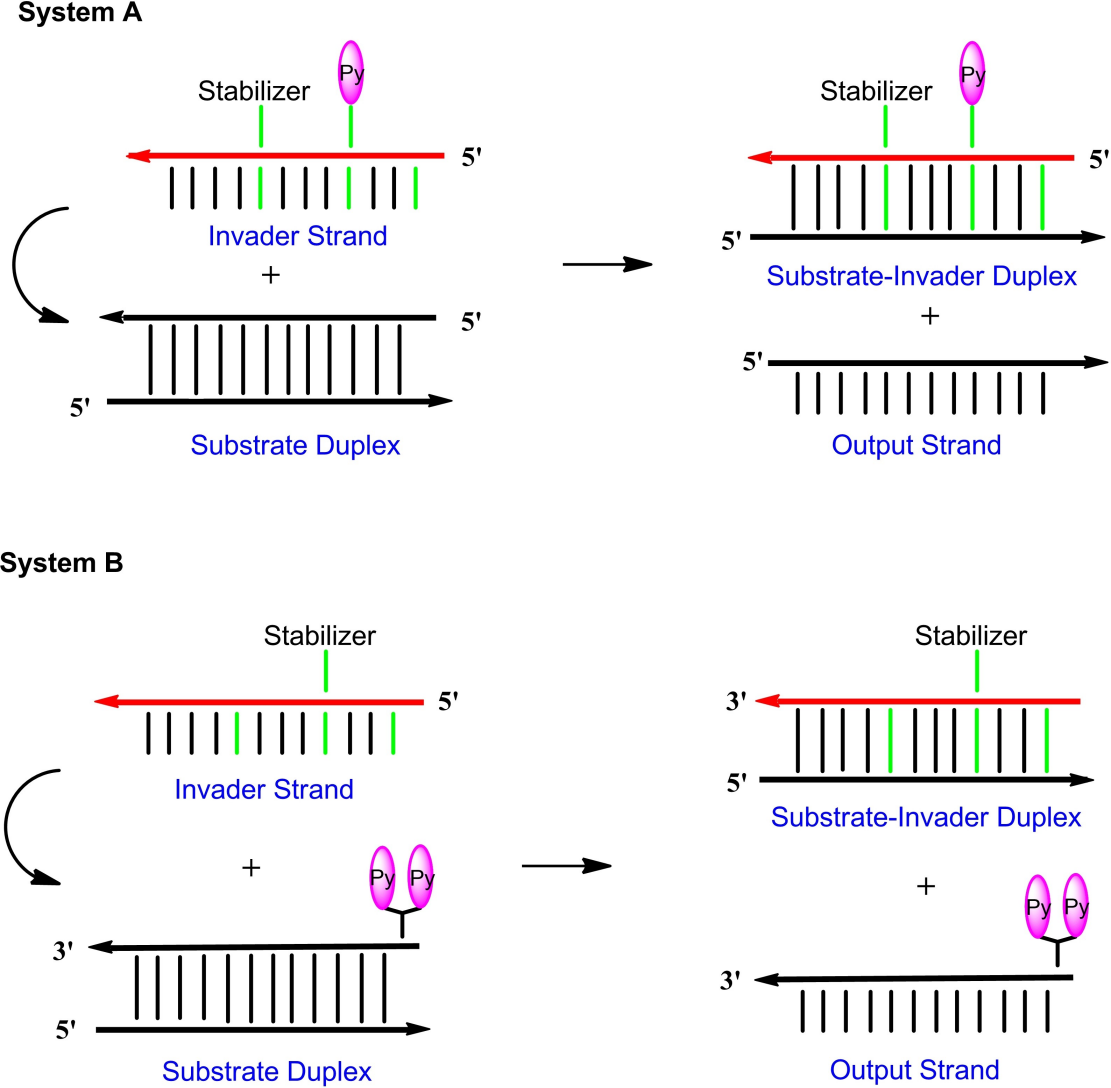
Schematic view of the starting and the end point of the displacement reactions according to system A and system B.

To monitor the progress of strand displacement reactions, pyrene fluorescence was utilized. To this end, the new pyrene sensors **4** and **5** were developed. In sensor **4** pyrene is linked to the nucleobase, in sensor **5** two proximal pyrene residues are connected to an abasic linker unit. The phosphoramidite of the bis‐pyrene sensor **5** is described in this work, pyrene sensor **4** is accessible by click chemistry on oligonucleotides. Scheme 1 shows the principle of the strand displacement reaction for systems A and B using base pair stabilizers. In system A, a single pyrene residue is part of the invader, whereas a bis‐pyrene linker is part of the target duplex in system B and is later part of the output strand. The dendritic pyrene linker forms an extension of the backbone at the 5’‐site. The single pyrene in system A shows monomer emission, whereas excimer emission is observed for the bis‐pyrene linker in system B. Stabilizers **1** and **2** are expected to act as the driving force for the displacement in both systems.

### Syntheses of stabilizers and pyrene sensors for incorporation in DNA

**Scheme 2:**
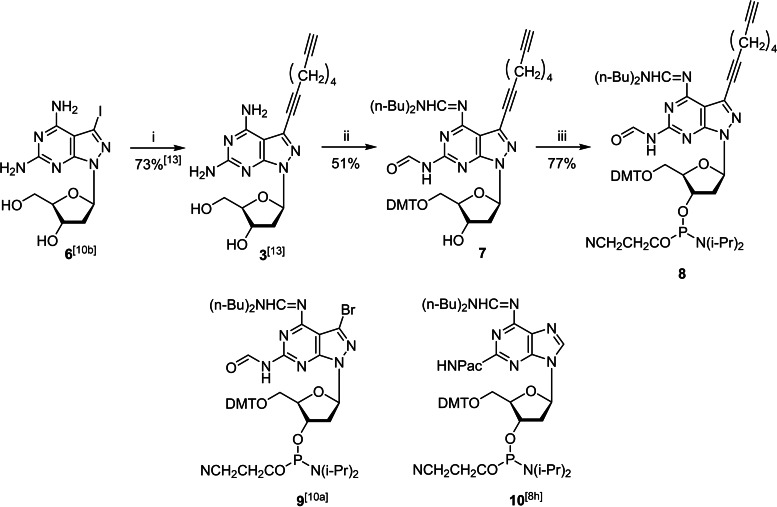
Synthesis of phosphoramidite **8** and structures of building blocks **9** and **10** used in this study. *Reagents and conditions*: (i) *Sonogashira* cross‐coupling: alkyne, Pd(0)(PPh_3_)_4_, Et_3_N, CuI, DMF, rt; (ii) a) *N,N‐*dibutylformamide dimethylacetal, MeOH, 40 °C, 2 h; b) 4,4′‐dimethoxytrityl chloride, pyridine, rt; (iii) NC(CH_2_)_2_OP(Cl)N(*i*‐Pr)_2_, DIPEA, DCM, rt.

Key compounds for the synthesis of modified invader oligonucleotides are the phosphoramidites **8–10**, that are used together with standard phosphoramidites to synthesize 12‐mer oligonucleotides used in the displacement studies. Phosphoramidites **9** and **10** have been already described by our laboratory;[^8h^, ^10a^] the synthesis of phosphoramidite **8** is reported herein (Scheme 2).

Starting material for the octadiynyl nucleoside **3** was the 2,6‐diamino‐7‐iodo nucleoside **6**.^[10b]^ *Sonogashira* cross‐coupling performed with **6** in anhydrous DMF and excess of 1,7‐octadiyne afforded the side chain derivative **3** in 73 % yield.^[13]^ Then, both amino groups of nucleoside **3** were protected. Protecting groups were carefully chosen, as the 2‐ and 6‐amino groups differ in reactivity. For the related 7‐bromo‐2,6‐diamino nucleoside **2** the combination of a formyl group for 2‐amino protection and a dibutylamidine residue for the 6‐amino group was found to be the optimal combination.^[10a]^ The same strategy was used for compound **3**. Reaction of **3** with *N*,*N*‐dibutylformamide dimethylacetal gave the 2,6‐bis‐amidine nucleoside together with traces of the 2‐formyl‐6‐amidine compound as detected by TLC (CH_2_Cl_2_/MeOH, 9 : 1). After evaporation, the reaction mixture was directly used without purification in the next step. Tritylation with DMT−Cl was performed under standard conditions. As reported for other nucleosides,[^9b^, ^10a^] the amidine group at the 2‐position is selectively hydrolyzed during the work‐up procedure and the formyl derivative **7** was isolated in 51 % yield over 2 steps. Finally, phosphitylation of **7** with chloro(2‐cyanoethoxy)(diisopropylamino)phosphine gave the phosphoramidite **8** in 77 %.

**Scheme 3:**
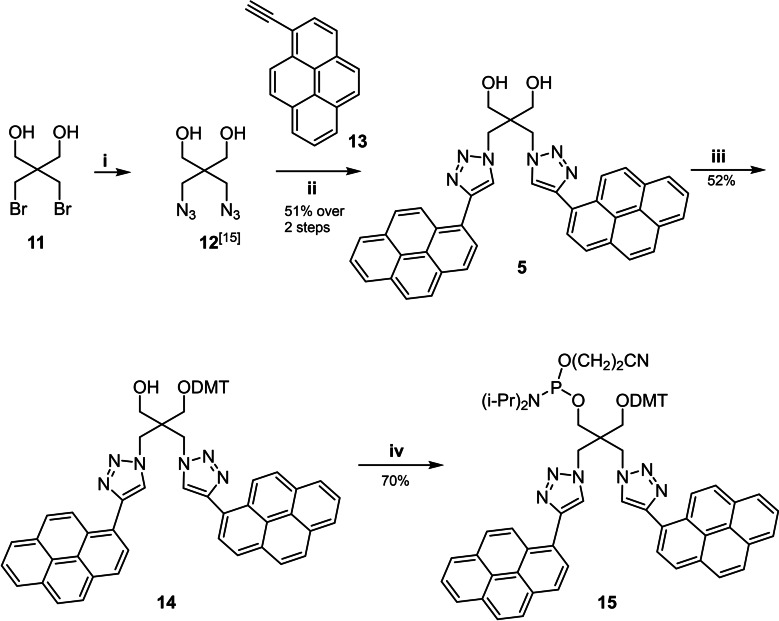
*Reagents and conditions*: (i) NaN_3_, DMF, 110 °C;^[15]^ (ii) CuSO_4_•5H_2_O, sodium ascorbate, THF/H_2_O (3 : 1), 80 °C; (iii) 4,4′‐dimethoxytrityl chloride, pyridine, rt; (iv) NC(CH_2_)_2_OP(Cl)N(*i*‐Pr)_2_, DIPEA, DCM, rt.

The phosphoramidite **15** containing two pyrene residues attached to a dendritic linker was prepared by the route shown in Scheme 3. Pyrene alkyne **13** ^[14]^ was clicked to the dendritic bis‐azide **12** ^[15]^ in *t*‐BuOH/H_2_O/THF with CuSO_4_/ascorbic acid as catalysts. The bis‐pyrene click adduct **5** was purified by column chromatography and obtained in 51 % yield over 2 steps. Then, 4,4‐dimethoxytritylation was performed (→**14**, 52 %). Finally, phosphitylation furnished phosphoramidite **15** (70 %). We became inspired to construct a bis‐pyrene sensor by our own studies on 1,3‐pronanediol linkers and on pyrene double click functionalization performed on tripropargylamine nucleosides and oligonucleotides.^[16]^ Further, motivation came from the work of the Yamana group and Saito and earlier work of Letsinger.^[17]^ Yamana developed a linker similar to ours that differ in the connectivity of the pyrene residues to the propanediole unit.^[17b]^ As the synthesis of our bis‐pyrene sensor is significantly shorter than that of Yamana, we took advantage of this matter.

All newly synthesized compounds were characterized by ESI‐TOF mass spectra, extinction coefficients and by ^1^H‐, ^13^C NMR spectra. ^1^H‐^13^C correlated (HMBC and HSQC) NMR spectra were used to assign the ^13^C NMR signals. For details, see the Experimental section, for data Tables S1, S2, Supporting Information and for spectra, Figures S5‐S31, Supporting Information.

### Synthesis of functionalized oligonucleotides and their duplex stability

For the construction of the strand displacement systems, a series of oligonucleotides were synthesized. Synthesis was performed on solid‐phase with modified phosphoramidites **8–10** and **15** (bis‐pyrene) and employed together with those of canonical DNA. Single and multiple incorporations of modified nucleosides were performed. Coupling yields were always higher than 95 %. After synthesis, oligonucleotides were cleaved from the solid support and deprotected in conc. 28 % aq. NH_3_ at 55 °C for 12 h. Detritylation was performed with 2.5 % dichloroacetic acid in CH_2_Cl_2_. Oligonucleotides were purified before and after detritylation by reversed‐phase HPLC on a RP‐18 column. HPLC purity profiles are documented in the Supporting Information (Figure S3, Supporting Information). New oligonucleotides are shown in Table 1 together with their molecular masses determined by MALDI‐TOF mass spectrometry.

**Table 1:** Synthesized oligonucleotides and their molecular masses determined by MALDI‐TOF mass spectrometry.

Entry	Oligonucleotides	M.W. calcd.^[a]^ exp.^[b]^	Entry	Oligonucleotides	M.W. calcd.^[a]^ exp.^[b]^
ODN‐**1**	5’‐d(TAG GTC AAT ACT)^[8h]^	–	ODN‐**2**	5’‐d(AGT ATT GAC CTA)^[8h]^	–
ODN‐**3**	5’‐d(AGT **1**TT GAC CTA)^[8h]^	3659.4 3660.3	ODN‐**4**	5’‐d(**1**GT 1TT G**1** C CTA)^[8h]^	3689.4 3689.2
ODN‐**5**	5’‐d(AGT **2**TT GAC CTA)^[8h]^	3738.3 3738.3	ODN‐**6**	5’‐d(**2**GT **2**TT G**2** C CTA)^[8h]^	3926.1 3927.7
ODN‐**7**	5’‐d(**2**GT **3**TT G**2** C CTA)	3952.4 3951.7	ODN‐**8**	5’‐d(**2**GT **4**TT G**2** C CTA)	4209.7 4209.3
ODN‐**9**	5’‐d(AGT **3**TT G**2** C CTA)	3858.5 3857.9	ODN‐**10**	5’‐d(AGT 4TT G**2** C CTA)	4115.8 4115.0
ODN‐**11**	5’‐d(**2**GT **3**TT GAC CTA)	3858.5 3858.2	ODN‐**12**	5’‐d(**2**GT **4**TT GAC CTA)	4115.8 4114.9
ODN‐**13**	5’‐d(**5**AGT ATT GAC CTA)	4346.1 4345.7			
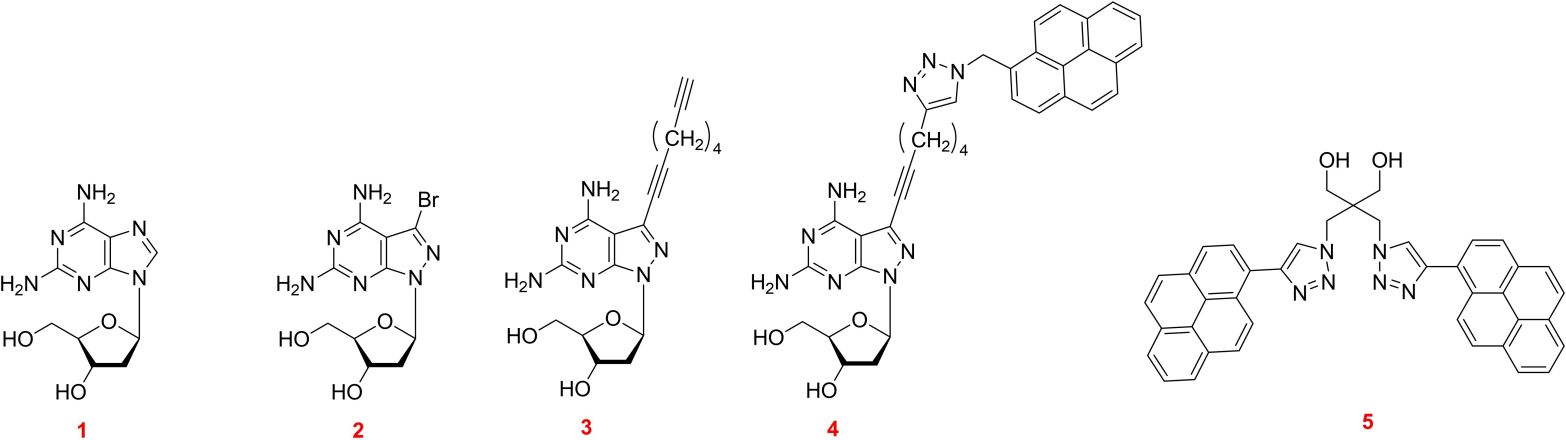					

[a] Calculated on the basis of the molecular mass of [*M*+H]^+^. [b] Determined by MALDI‐TOF mass‐spectrometry as [*M+*H]^+^ in the linear positive mode.

In order to prove the applicability of modified oligonucleotides containing stabilizer **1** or **2** for the toehold‐ free displacement systems A and B, thermodynamic stability of duplexes was investigated. To this end, single‐stranded oligonucleotides were hybridized, thermal melting curves were measured, *T*_m_ values were determined and thermodynamic data were calculated (Tables 2, 3, Tables S3‐5, and Figures S3, S4, Supporting Information). In all experiments, the single strand concentration was 5 μM and the buffer solution contained 100 mM NaCl, 10 mM MgCl_2_, and 10 mM Na‐cacodylate (pH 7.0).

**Table 2:** *T*_m_ values of oligonucleotide duplexes before and after addition of the invader strand containing 2,6‐diamino nucleosides **1**–**4** or the bis‐pyrene linker **5**.^[a]^

Displacement System A before Invader Input	*T*_m_^[b]^ [°C]	Displacement System A after Invader Input with Output Strand	*T*_m_^[c]^ [°C]	Displacement System A before Invader Input	*T*_m_^[b]^ [°C]	Displacement System A after Invader Input with Output Strand	*T*_m_^[c]^ [°C]
Substrate Duplex 5’‐d(TAG GTC AAT ACT) (ODN‐**1**) 3’‐d(ATC CAG TTA TGA) (ODN‐**2**) Invader Input 3’‐d(ATC CAG TT **1** TGA) (ODN‐**3**)	47	5’‐d(TAG GTC AAT ACT) (ODN‐**1**) 3’‐d(ATCCAGTT **1** TGA) (ODN‐**3**) + 3’‐d(ATC CAG TTA TGA) (ODN‐**2**)	52	Substrate Duplex 5’‐d(TAG GTC AAT ACT) (ODN‐**1**) 3’‐d(ATC CAG TTA TGA) (ODN‐**2**) Invader Input 3’‐d(ATC CAG TT **2** TGA) (ODN‐**5**)	47	5’‐d(TAG GTC AAT ACT) (ODN‐**1**) 3’‐d(ATCCAGTT **2** TGA) (ODN‐**13**) + 3’‐d(ATC CAG TTA TGA) (ODN‐**2**)	54
Substrate Duplex 5’‐d(TAG GTC AAT ACT) (ODN‐**1**) 3’‐d(ATC CAG TTA TGA) (ODN‐**2**) Invader Input 3’‐d(ATC C **1** G TT **1** TG **1** ) ( ODN‐**4**)	47	5’‐d(TAG GTC AAT ACT) (ODN‐**1**) 3’‐d(ATC C **1** G TT **1** TG **1** ) (ODN‐**4**) + 3’‐d(ATC CAG TTA TGA) (ODN‐**2**)	52	Substrate Duplex 5’‐d(TAG GTC AAT ACT) (ODN‐**1**) 3’‐d(ATC CAG TTA TGA) (ODN‐**2**) Invader Input 3’‐d(ATC C **2** G TT **2** TG **2** ) (ODN‐**6**)	47	5’‐d(TAG GTC AAT ACT) (ODN‐**1**) 3’‐d(ATC C **2** GTT **2** TG **2** ) (ODN‐**6**) + 3’‐d(ATC CAG TTA TGA) (ODN‐**2**)	60
Substrate Duplex 5’‐d(TAG GTC AAT ACT) (ODN‐**1**) 3’‐d(ATC CAG TTA TGA) (ODN‐**2**) Invader Input 3’‐d(ATC C **2** G TT**3** TG **2** ) (ODN‐**7**)	47	5’‐d(TAG GTC AAT ACT) (ODN‐1) 3’‐d(ATC C **2** G TT **3** TG **2** ) (ODN‐**7**) + 3’‐d(ATC CAG TTA TGA) (ODN‐**2**)	58	Substrate Duplex 5’‐d(TAG GTC AAT ACT) (ODN‐**1**) 3’‐d(ATC CAG TTA TGA) (ODN‐**2**) Invader Input 3’‐d(ATC C **2** G TT **4** TG **2** ) (ODN‐**8**)	47	5’‐d(TAG GTC AAT ACT) (ODN‐**1**) 3’‐d(ATC C **2** G TT **4** TG **2** ) (ODN‐**8**) + 3’‐d(ATC CAG TTA TGA) (ODN‐**2**)	66
Substrate Duplex 5’‐d(TAG GTC AAT ACT) (ODN‐**1**) 3’‐d(ATC CAG TTA TGA) (ODN‐**2**) Invader Input 3’‐d(ATC C **2** G TT **3** TGA) (ODN‐**9**)	47	5’‐d(TAG GTC AAT ACT) (ODN‐**1**) 3’‐d(ATC C **2** G TT **3** TGA) (ODN‐**9**) + 3’‐d(ATC CAG TTA TGA) (ODN‐**2**)	56	Substrate Duplex 5’‐d(TAG GTC AAT ACT) (ODN‐**1**) 3’‐d(ATC CAG TTA TGA) (ODN‐**2**) Invader Input 3’‐d(ATC C **2** G TT**4** TGA) (ODN‐**10**)	47	5’‐d(TAG GTC AAT ACT) (ODN‐**1**) 3’‐d(ATC C **2** G TT**4** TGA) (ODN‐**10**) + 3’‐d(ATC CAG TTA TGA) (ODN‐**2**)	67
Substrate Duplex 5’‐d(TAG GTC AAT ACT) (ODN‐**1**) 3’‐d(ATC CAG TTA TGA) (ODN‐**2**) Invader Input 3’‐d(ATC CAG TT **3** TG **2** ) (ODN‐**11**)	47	5’‐d(TAG GTC AAT ACT) (ODN‐**1**) 3’‐d(ATC CAG TT **3** TG **2** ) (ODN‐**11**) + 3’‐d(ATC CAG TTA TGA) (ODN‐**2**)	54	Substrate Duplex 5’‐d(TAG GTC AAT ACT) (ODN‐**1**) 3’‐d(ATC CAG TTA TGA) (ODN‐**2**) Invader Input 3’‐d(ATC CAG TT **4** TG **2** ) (ODN‐**12**)	47	5’‐d(TAG GTC AAT ACT) (ODN‐**1**) 3’‐d(ATC CAG TT **4** TG **2** ) (ODN‐**12**) + 3’‐d(ATC CAG TTA TGA) (ODN‐**2**)	63
Substrate Duplex 5’‐d(TAG GTC AAT ACT) (ODN‐**1**) 3’‐d(ATC CAG TTA TGA **5** ) (ODN‐**13**) Invader Input 3’‐d(ATC C **1** G TT **1** TG **1** ) (ODN‐**4**)	50	5’‐d(TAG GTC AAT ACT) (ODN‐**1**) 3’‐d(ATC C **1** GTT **1** TG **1** ) (ODN‐**4**) + 3’‐d(ATC CAG TTA TGA **5** ) (ODN‐**13**)	53	Substrate Duplex 5’‐d(TAG GTC AAT ACT) (ODN‐**1**) 3’‐d(ATC CAG TTA TGA **5** ) (ODN‐**13**) Invader Input 3’‐d(ATC C **2** G TT **2** TG **2** ) (ODN‐**6**)	50	5’‐d(TAG GTC AAT ACT) (ODN‐**1**) 3’‐d(ATCC **2** GTT **2** TG **2** ) (ODN‐**6**) + 3’‐d(ATC CAG TTA TGA **5** ) (ODN‐**13**)	59

^[a]^ Measured at 260 nm at a concentration of 5 μM+5 μM single strand at a heating rate of 1.0 °C/min in 100 mM NaCl, 10 mM MgCl_2_, and 10 mM Na‐cacodylate (pH 7.0). ^[b]^ *T*_m_ values were calculated from the heating curves using the program *Meltwin 3.0*.^[18]^ ^[c]^ *T*_m_ values were calculated from the heating curves after adding the corresponding invader strands with 5 μM concentration. The standard deviation for the *T*_m_ values is ±0.5 °C. For the formulas of compounds **1**–**5**, see the legend to Table 3.

**Table 3:** *T*_m_ values of oligonucleotide duplexes containing 2,6‐diamino nucleosides **1**–**4** or the bis‐pyrene linker **5**.^[a]^

Duplexes Only	*T*_m_^[b]^ [°C]	Duplexes Only	*T*_m_^[b]^ [°C]	Duplexes Only	*T*_m_^[b]^ [°C]	Duplexes Only	*T*_m_^[b]^ [°C]
5’‐d(TAG GTC AAT ACT) (ODN‐**1**) 3’‐d(ATC CAG TTA TGA) (ODN‐**2**)	47	5’‐d(TAG GTC AAT ACT) (ODN‐**1**) 3’‐d(ATC C **2** G TT **3** TG **2** ) (ODN‐**7**)	58	5’‐d(TAG GTC AAT ACT) (ODN‐**1**) 3’‐d(ATC C **2** G TT **4** TG **2** ) (ODN‐**8**)	66	5’‐d(TAG GTC AAT ACT) (ODN‐**1**) 3’‐d(ATC CAG TTA TGA **5** ) (ODN‐**13**)	50
5’‐d(TAG GTC AAT ACT) (ODN‐**1**) 3’‐d(ATC CAG TT **1** TGA) (ODN‐**3**)	50	5’‐d(TAG GTC AAT ACT) (ODN‐**1**) 3’‐d(ATC C **2** G TT **3** TGA) (ODN‐**9**)	57	5’‐d(TAG GTC AAT ACT) (ODN‐**1**) 3’‐d(ATC C **2** G TT **4** TGA) (ODN‐**10**)	67	5’‐d(TAG GTC AAT ACT) (ODN‐**1**) 3’‐d(ATC CAG TT **2** TGA) (ODN‐**2**)	54
5’‐d(TAG GTC AAT ACT) (ODN‐**1**) 3’‐d(ATC C **1** G TT **1** TG **1** ) (ODN‐**4**)	52	5’‐d(TAG GTC AAT ACT) (ODN‐**1**) 3’‐d(ATC CAG TT **3** TG **2** ) (ODN‐**11**)	54	5’‐d(TAG GTC AAT ACT) (ODN‐**1**) 3’‐d(ATC CAG TT **4** TG **2** ) (ODN‐**12**)	62	5’‐d(TAG GTC AAT ACT) (ODN‐**1**) 3’‐d(ATC C **2** G TT **2** TG **2** ) (ODN‐**6**)	59
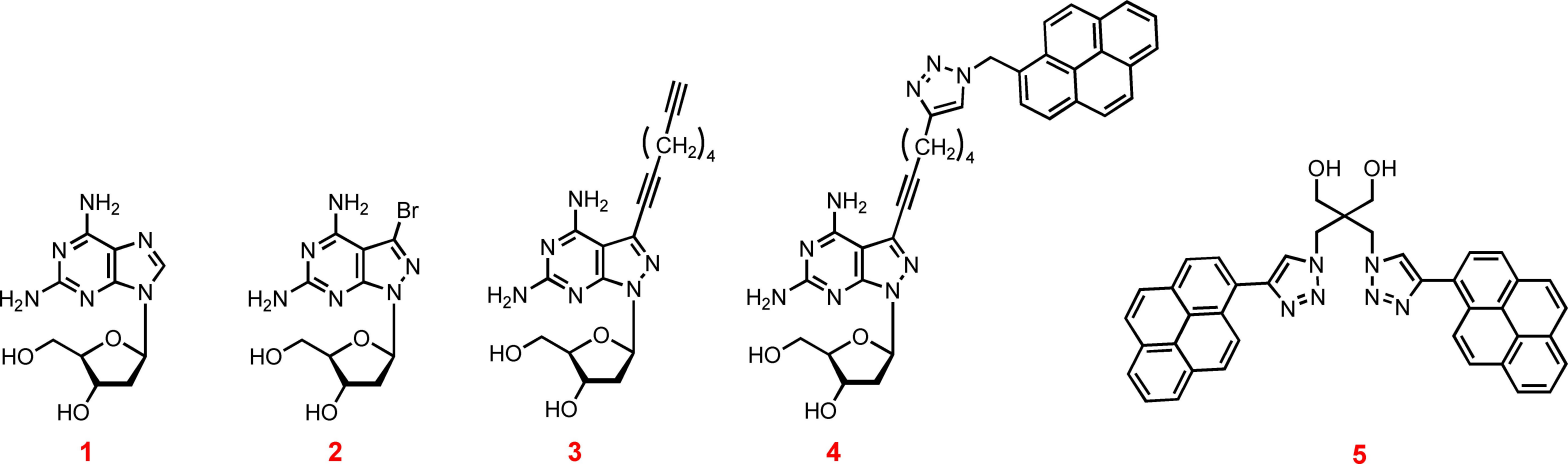							

^[a]^ Measured at 260 nm at a concentration of 5 μM+5 μM single strand at a heating rate of 1.0 °C/min in 100 mM NaCl, 10 mM MgCl_2_, and 10 mM Na‐cacodylate (pH 7.0). ^[b]^ *T*_m_ values were calculated from the heating curves using the program *Meltwin 3.0*.^[18]^ The standard deviation for the *T*_m_ values is ±0.5 °C. “Duplexes Only” means duplexes without the presence of invader or released strands.

The unmodified duplex ODN‐**1**•ODN‐**2** that is commonly used in our laboratory was the basis for all further experiments in this work. It shows a *T*_m_ value of 47 °C (Table 2, left column). The *T*_m_ value increases to 52 °C when the output strand ODN‐**2** was displaced by the invader strand ODN‐**4** containing three 2,6‐diaminopurine nucleosides **1** in place of three dA residues. When the same was performed with invader strand ODN‐**6** (stabilizer **2**), the stability increase was significantly stronger (*T*_m_=60 °C, ODN‐**1**•ODN‐**6**). Replacing the central stabilizer **2** by the octadiynyl nucleoside **3** (ODN‐**1**•ODN‐**7**) gave a *T*_m_ of 58 °C. Functionalization of **3** with pyrene (→**4**) yielded additional stabilization (*T*_m_=66 °C, ODN‐**1**•ODN‐**8**) (for details, see the Experimental section). Duplex stability depends on the number of incorporations and their position. Earlier, it was reported that stabilizer **2** shows favourable proton donor properties compared to the 2,6‐diaminopurine nucleoside **1**. Most likely the enhanced amide character of the 2‐amino group of **2** compared to **1** results from the changes of electronic properties of the two ring systems (8‐aza‐7‐deazapurine vs. purine) and the electronic contribution of the halogen atom. Changes in base stacking and hydration have to be also considered. The same can be considered for stabilizer **3** and the pyrene sensor **4**. From *T*_m_ values and thermodynamic data, it is obvious that multiple incorporations of the 2,6‐diamino nucleosides **2**, **3** and **4** fulfil the expectation as stabilizers of the dA‐dT base pairs in the displacement system. According to the higher stability of modified duplexes containing nucleoside **2**, a clear advantage is observed for nucleoside **2** with respect to **1**. Apparently, the third hydrogen bond is stronger in the **2**‐dT base pair as in the **1**‐dT pair and develops the same stability as in the dG‐dC pair.[^8^, ^10^]

The octadiynyl modified nucleoside **3** with a clickable side chain was used to introduce pyrene. The terminal bis‐pyrene linker in duplex ODN‐**1**•ODN‐**13** induces only marginal but measurable stabilization. The *T*_m_ values in the presence or absence of invaders or output molecules are very similar to those of the “duplexes only” (Table 3).

### Isothermal displacement reaction with oligonucleotide invaders containing base pair stabilizers 1 and 2 and pyrene or bis‐pyrene sensors

DNA strand displacement is a dynamic process in which a single‐stranded invader DNA replaces a strand with the same or similar strand recognition in the duplex target. The displacement reaction starts usually at the termini of duplexes as terminal base pairs are less stable than internal pairs.[^1^, ^2^, ^3^] The reaction is driven by the enthalpy change between the substrate duplex and the displaced duplex. The kinetics depends on the size of the duplex, the invader itself, and on many other parameters such as strand concentration, temperature or ions present in solution. When the displacement is finished, the back reaction is unfavorable, when the free energy difference between substrate and displaced duplex is substantial.

When sensors are part of a displacement system, they affect the stability of the parent duplex.^[19]^ Consequently, all thermodynamic and kinetic data obtained for common displacement systems including those of the often used FRET system do not display the real situation of the systems in the absence of sensors. This is also visible from stability data of Tables 2, 3 (duplex stabilities in the presence or the absence of pyrene sensors). Nevertheless, sensors are required to monitor displacement and fluorescence is the method of choice.

**Figure 2:**
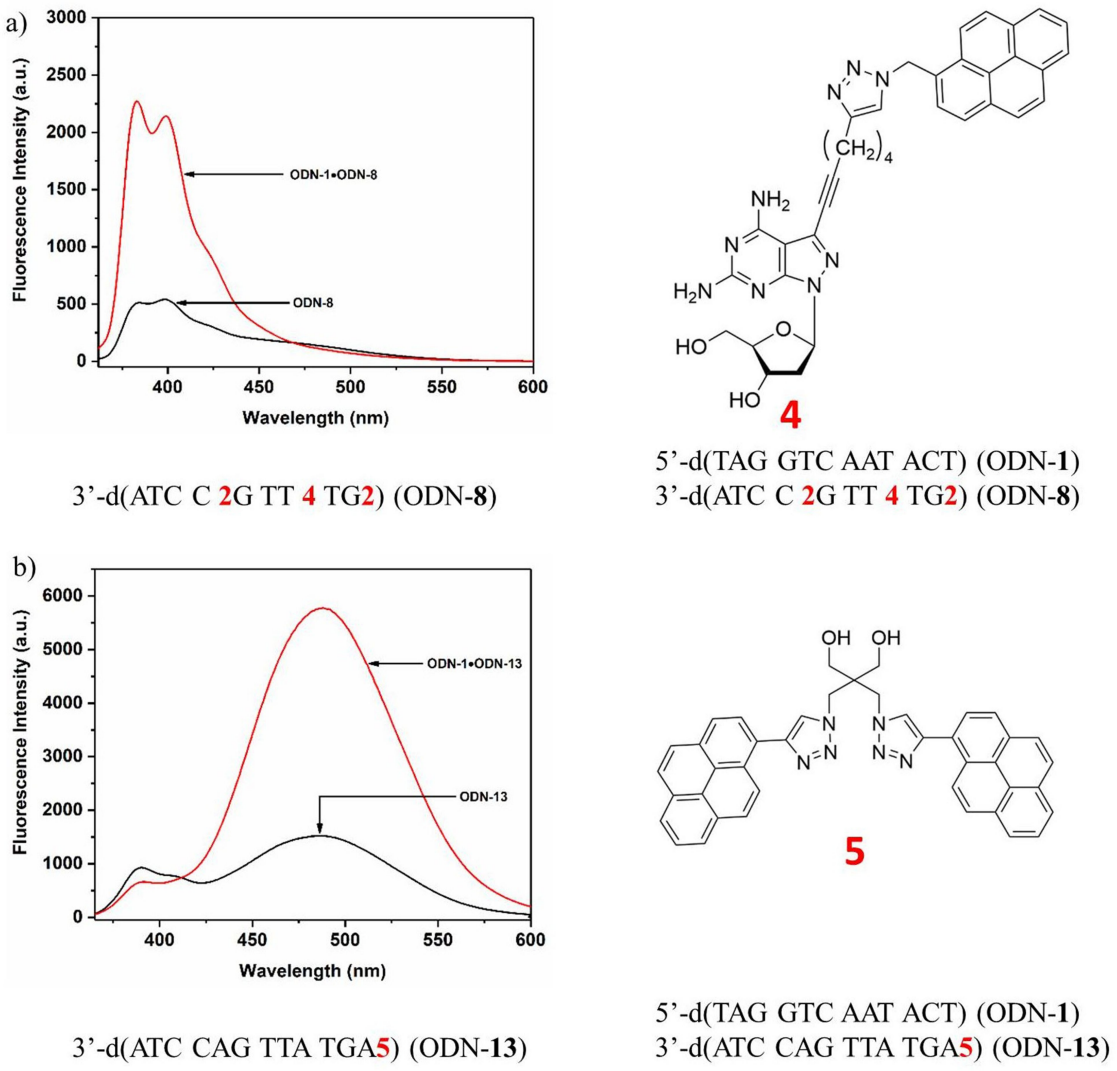
Fluorescence of a) oligonucleotide ODN‐**8** and the corresponding duplex 5’‐d(TAG GTC AAT ACT) (ODN‐**1**) • 3’‐d(ATC C**2**G TT**4** TG**2**) (ODN‐**8**); b) oligonucleotide ODN‐**13** and the corresponding duplex 5’‐d(TAG GTC AAT ACT) (ODN‐**1**) • 3’‐d(ATC CAG TTA TG**5**) (ODN‐**13**). The excitation wavelengths of for the sensors are 345 nm for **4** and 347 nm for **5**. The excitation and emission bandpass was 10 nm for all measurements.

Herein, fluorescence changes of pyrene are used.^[20]^ To this end, the fluorescence of single strands and duplexes decorated with pyrene residues were measured and compared. To this end, 5 μM (sensor **4**) or 2.5 μM (sensor **5**) solutions of single strands in buffer were prepared and fluorescence was measured before and after addition of complementary strands (Figure 2, Figure S2, Supporting Information). Invader oligonucleotides with sensor **4** show monomer emission around 384 and 398 nm, whereas oligonucleotides with the dendritic bis‐pyrene linker **5** show excimer emission at 488 nm. The oligonucleotides were excited at 345 nm (sensor **4**) and 347 nm (sensor **5**).

From Figures 2a and 2b significant fluorescence changes between single strands and duplexes are apparent. This is the case for oligonucleotides with sensor **4** having the pyrene residue connected to the nucleobase as well as for oligonucleotides with the terminal bis‐pyrene linker **5**. According to this, their application in strand displacement was verified.

**Scheme 4:**
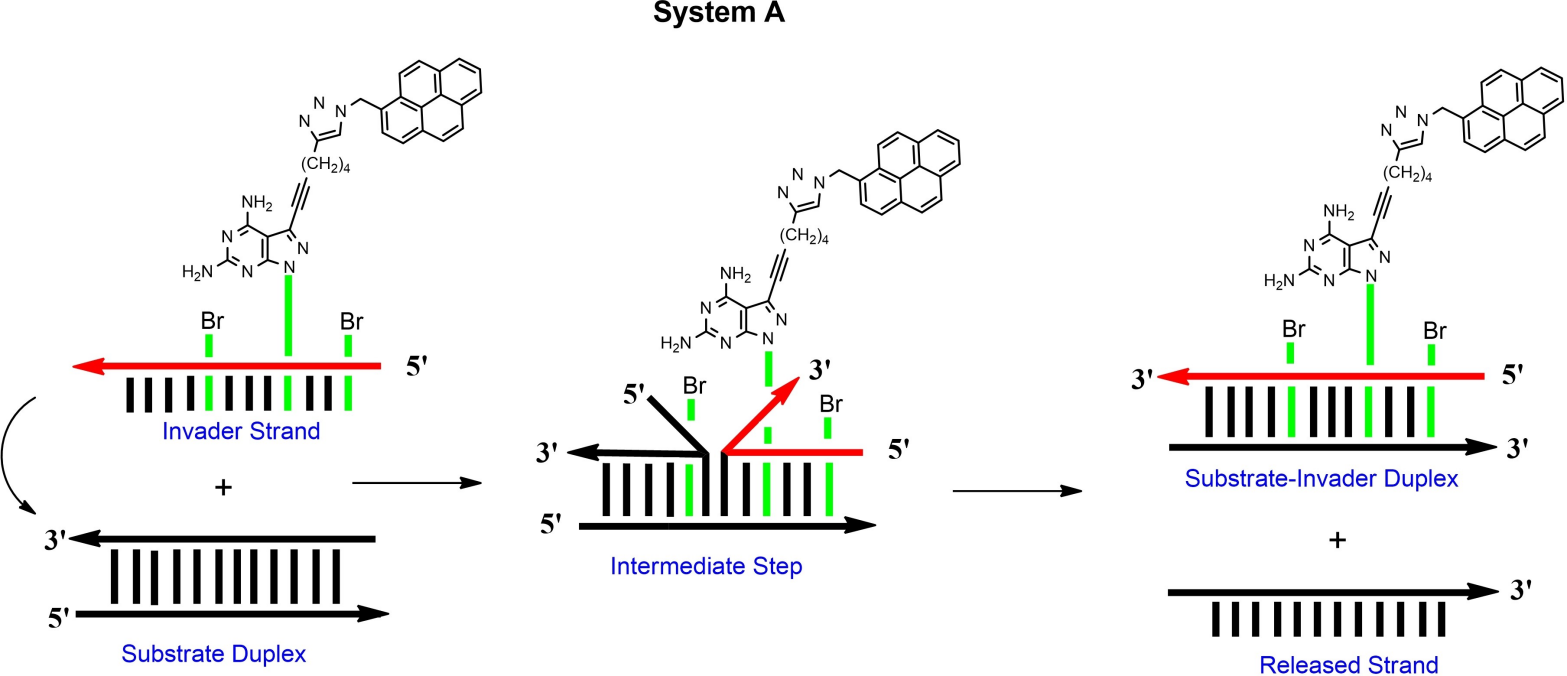
Displacement reaction with pyrene sensor **4** according to system A.

**Figure 3:**
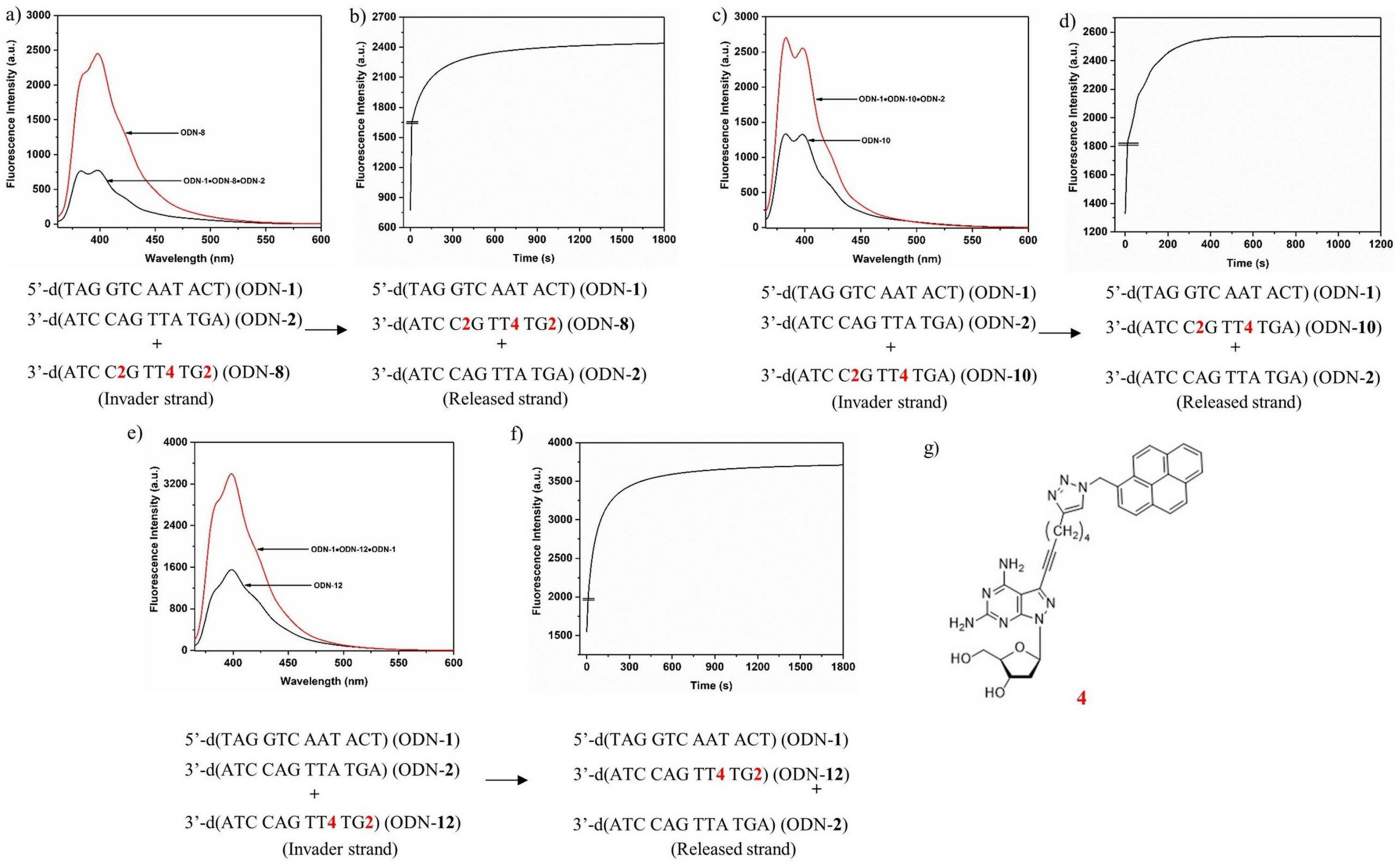
Displacement reactions according to system A. a), c), d) Fluorescence spectra showing the starting and the end point of the particular displacement reaction. b), d), f) Reaction progress followed by fluorescence changes. g) Structure of the nucleoside pyrene sensor used in system A. All measurements were performed at an excitation wavelength of 345 nm with 5 μM single‐strand concentration in 100 mM NaCl, 10 mM MgCl_2_, and 10 mM Na‐cacodylate (pH 7.0).

Next, the progress of the displacement reactions was followed by time‐dependent recording of pyrene fluorescence change. On molecular level, the invader strand targets the fraying ends of the substrate duplex and displacement progresses by strand migration (Scheme 4). To this end, the invader single strand ODN‐**8** was added to the standard duplex ODN‐**1•**ODN‐**2**. The reaction was performed at 22 °C and the pyrene monomer emission increase was followed. Due to the formation of the new invader‐substrate duplex ODN‐**1•**ODN‐**8**, the fluorescence intensity increased by about a factor of 3 (Figure 3, left spectrum). Excitation occurred at 345 nm and emission at 398 nm. The displacement reaction was performed with a series of oligonucleotides containing stabilizer **2** in which the number of incorporations and position was altered. Plotting the fluorescence change at an emission wavelength of 398 nm against the time scale reveals an exponential growth in the beginning of the displacement reaction, which ends in an asymptotic behavior after prolonged reaction time. The graphs are shown in Figure 3 together with the steady‐state fluorescence curves of the starting duplex and the product duplex.

Under those conditions, displacement is fast. From the time‐dependent measurements, half‐life values of below 100 s were calculated. The final equilibrium of the displacement reaction was observed after several minutes. The reaction progress of other displacements performed on invaders with a different number of stabilizers is similar. Displacement monitoring started about 5 s after the initiation of the reaction. The delay was the result of sample mixing before measurement and the start of the fluorescence spectrometer. From these experiments, it is obvious that the base pair stabilizer **2** can be used to drive the displacement reaction and a toehold is unnecessary.

**Scheme 5:**
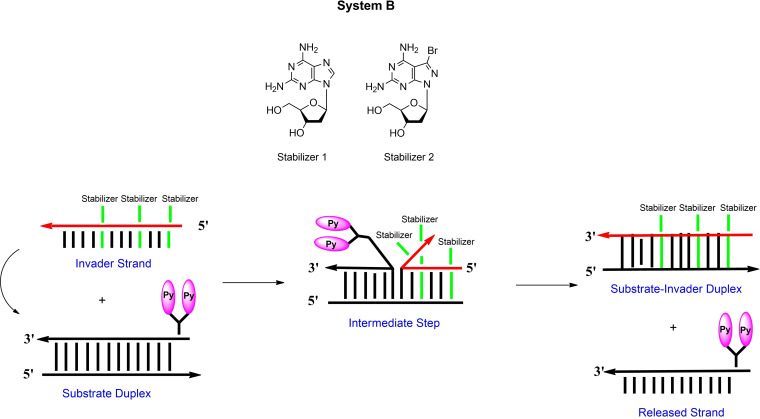
Displacement reaction according to system B using pyrene sensor **5** and stabilizers **1** and **2**.

**Figure 4:**
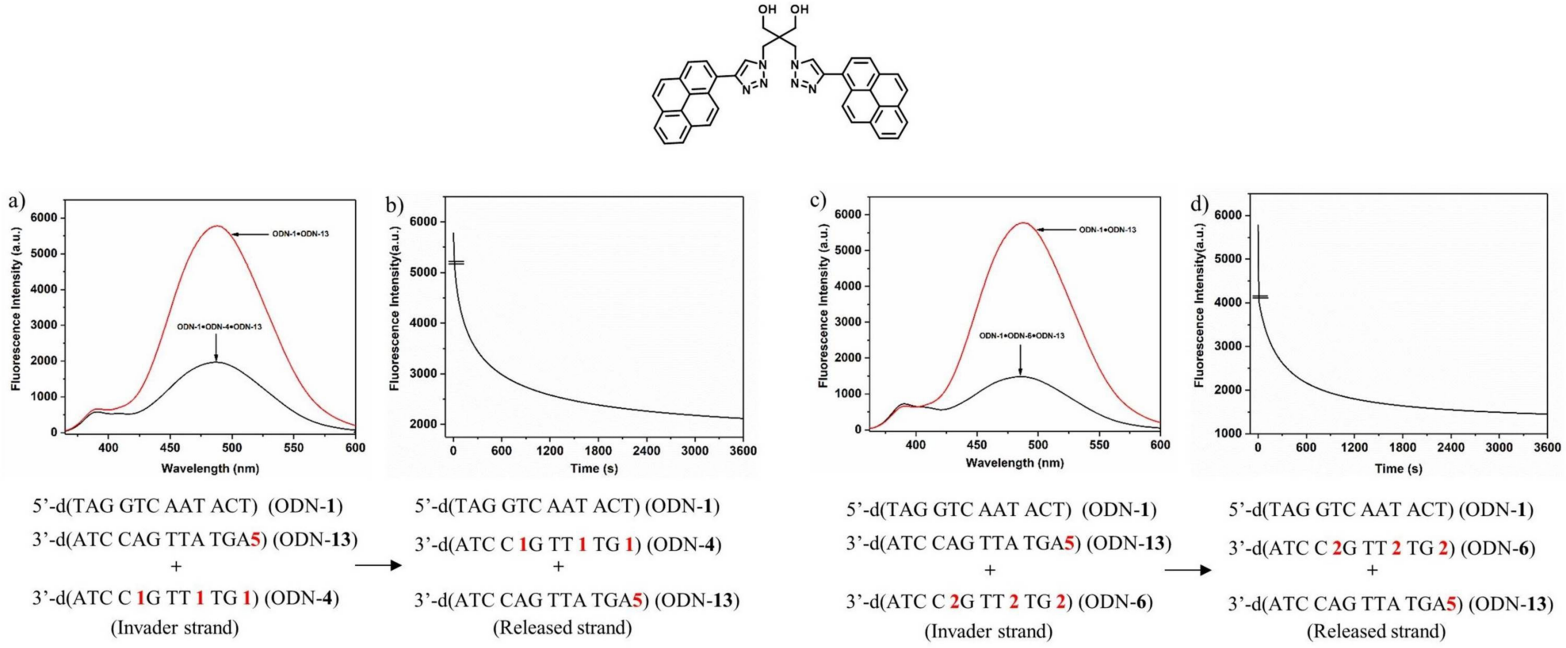
Displacement reactions according to system B. a), c) Fluorescence spectra showing the starting and the end point of the particular displacement reaction. b), d) Reaction progress followed by fluorescence changes. Up: Structure of the pyrene sensor used in System B. The bandpass for excitation and emission was 10 nm. All measurements were performed with an excitation wavelength of 347 nm with 2.5 μM single‐strand concentration in 100 mM NaCl, 10 mM MgCl_2_, and 10 mM Na‐cacodylate (pH 7.0).

In system A, stabilizer and sensor were implemented in the single stranded invader. In system B, the sensor is part of the target duplex (Scheme 5). System B was designed to compare the displacement reaction of oligonucleotide invaders containing either three incorporations of stabilizer **1** or three incorporations of stabilizer **2**. After displacement, only the stabilizers **1** or **2** are part of the final duplexes, and the sensor is released with the output strand. According to the bis‐pyrene fluorescence behavior, excimer emission decreases after displacement. From Figure 4, it is apparent that the less efficient stabilizer **1** leads to a slower displacement as the effective stabilizer **2**. This is in line with the higher thermodynamic stability of the duplexes induced by stabilizer **2** compared to that of stabilizer **1**. It slows‐down the reaction. Other factors can be excluded as system B differs only in the structures of stabilizers. According to the stabilizing property of the clickable compound **3** and its ability to develop fluorescence after click reaction (→**4**), this compound comprises already the capacity to act as base pair stabilizer and as sensor in a combined system.

## Conclusions

Purine‐2,6‐diamine (2‐aminoadenine) and 8‐aza‐7‐deaza‐7‐bromopurine‐2,6‐diamine 2’‐deoxyribonucleosides **1** and **2** have been used to strengthen invader strand interactions in DNA strand displacement. They were incorporated in oligonucleotides and implemented in isothermal displacement systems. Displacement reactions were performed at 22 °C without transferring energy to the system. Displacement is driven by negative enthalpy changes between the target and the displaced duplex. The displacement was performed with 12‐mer oligonucleotides duplexes and single strand invaders with single and multiple incorporations of modified nucleosides. *T*_m_ data and thermodynamic values obtained from UV melting profiles were used to set up the displacement systems. From those, the applicability of the base stabilizers in strand displacement was established.

For strand displacement reactions, sensors were implemented that monitor the progress of the displacement. Two new environmental sensitive fluorescent pyrene sensors (**4** and **5**) were designed. In one sensor pyrene was connected to the nucleobase and was present in the invader strand, the other sensor contained a bis‐pyrene unit and was implemented in a dendritic linker. The constructions of both sensors base on click chemistry, and phosphoramidites were prepared for solid‐phase synthesis. One sensor shows monomer and the other excimer emission and both show fluorescence changes when they are part of duplexes or single strands. This fluorescence change was used to follow strand exchange in two systems; A and B. In both systems displacement is fast and efficient. Our work demonstrates that base pair stabilizers can expand the utility of displacement reactions. Toeholds as used in other displacement systems are not required. Other displacement set‐ups are possible on the basis of our results in the realm of DNA and RNA. Due to modified base incorporation, invader strand and displaced duplexes might be not cleaved by nucleolytic enzymes or cleavage will be retarded. This makes the systems applicable to living matter. Applications in chemical biology, nanotechnology or for diagnostic or therapeutic uses are feasible.

## Experimental Section

**General**: All chemicals and solvents were of laboratory grade as obtained from commercial suppliers and were used without further purification. Thin‐layer chromatography (TLC) was performed on TLC aluminium sheets covered with silica gel 60 F254 (0.2 mm). Flash column chromatography (FC): silica gel 60 (40‐60 μM) at 0.4 bar. UV‐spectra were recorded on a UV‐spectrophotometer: *λ*_max_ (ϵ) in nm, ϵ in dm^3^ mol^−1^ cm^−1^. NMR spectra were measured at 599.74 MHz, 399.89 MHz or 300.15 MHz for ^1^H, at 150.82 MHz, 100.56 MHz or 75.47 MHz for ^13^C and at 121.5 MHz for ^31^P. ^1^H‐^13^C correlated (HMBC, HSQC) NMR spectra were used for the assignment of the ^13^C signals (Tables S1, S2, Supporting Information). The *J* values are given in Hz; δ values in ppm relative to Me_4_Si as internal standard. For NMR spectra recorded in DMSO‐*d_6_*, the chemical shift of the solvent peak was set to 2.50 ppm for ^1^H NMR and 39.50 ppm for ^13^C NMR. ESI‐TOF mass spectra of nucleosides were recorded on a Micro‐TOF spectrometer.

**Oligonucleotide syntheses and characterization**: Solid‐phase oligonucleotide syntheses were performed with an ABI 392–08 synthesizer at 1 μmol scale (trityl‐on mode) employing the phosphoramidites **8–10**, as well as standard building blocks, giving an average coupling yield of over 95 %. After cleavage from the solid support, the oligonucleotides were deprotected in 28 % aqueous ammonia at 55 °C for 12 h. The 4,4’‐dimethoxytrityl containing oligonucleotides were purified by reversed‐phase HPLC (RP‐18) with a gradient system at 260 nm: (A) MeCN, (B) 0.1 m (Et_3_NH)OAc (pH 7.0)/MeCN, 95 : 5; gradient I: 0–3 min 10–15 % A in B, 3–15 min 15–50 % A in B; flow rate 0.7 mL min^−1^ mL min. The purified “trityl‐on” oligonucleotides were treated with 2.5 % CHCl_2_COOH/CH_2_Cl_2_ for 2 min at 8 °C to remove the 4,4’‐dimethoxytrityl residues. The detritylated oligomers were further purified by reversed phase HPLC with gradient II: 0–20 min 0–20 % A in B; 20–25 min, 20 % A in B; flow rate 0.7 mL min^−1^. The oligonucleotides were desalted on a reversed‐phase column (RP‐18) by using water for the elution of salts, and the oligonucleotides were eluted with H_2_O/MeOH (2 : 3). The oligonucleotides were lyophilized with a SpeedVac evaporator to yield colorless solids, which were frozen at −24 °C. The purity of all oligonucleotides was confirmed by RP‐18 HPLC (Figure S3, Supporting Information) and MALDI‐TOF mass spectrometry (Table 1). The extinction coefficients ϵ_260_ (H_2_O) of the nucleosides were determined as: dA 15 400, dG 11 700, dT 8800, dC 7300, **1** 8200, **2** 8700, **3** 10300, **4** 23800, **5** 12700 mol^−1^ dm^3^ cm^−1^nur . The extinction coefficients of the oligonucleotides were calculated from the sum of the extinction coefficients of their constituent nucleosides, with a hypochromic change of 20 % for the single strands.

## Fluorescence Studies

**General**: Fluorescence measurements were performed using a F‐7000 fluorescence spectrophotometer (Hitachi, Tokyo, Japan). Cleaned and dried Hellma analytics cuvettes (2 mL volume) with caps were used for the fluorescence study. All measurements were performed at ambient temperature (22 °C).

**Fluorescence of oligonucleotide duplexes**: 5 μM+5 μM (sensor **4**) or 2.5 μM+2.5 μM (sensor **5**) of single‐stranded oligonucleotides were added to 1 mL buffer (100 mM NaCl, 10 mM MgCl_2_, and 10 mM Na‐cacodylate, pH 7.0). To determine the excitation wavelengths of the sensors, UV spectra of oligonucleotides were measured. The long wavelengths UV maximum were used for the determination of the emission of the oligonucleotide. The monochromator was set to the long wavelength maximum of fluorescence emission, and the wavelength of the excitation monochromator is scanned across the desired excitation range. Then, the intensity of fluorescence is recorded on the detector as a function of excitation wavelength. The long‐wavelength maximum from the excitation spectrum was used to measure the emission spectra. Accordingly, oligonucleotides containing sensor **4** were excited at 345 nm, oligonucleotides incorporating sensor **5** were excited at 347 nm. Oligonucleotides with sensor **4** show monomer emission around 384 and 398 nm, oligonucleotides with the dendritic bis‐pyrene linker **5** show excimer emission at 488 nm. The UV absorbance was below 0.01 to exclude inner filter effects.

**Reaction progress of the strand displacement reactions**: To a solution containing 5 μM (sensor **4**) or 2.5 μM (sensor **5**) of the particular duplex in a fluorescence cuvette, the invader strand was added in the same concentration by a micro pipette. Then, the cuvette was shaken vigorously and set in the spectrophotometer. Then, time dependent fluorescence measurements were carried out at room temperature for a particular time interval. The instrument parameters were set as follows: Excitation wavelength (EX) 345 nm, emission wavelength (EM) 398 nm for system A, EX 347 nm, EM 488 nm for system B. Slit widths were set to 10 nm for excitation and 10 nm for emission. Fluorescence data were processed using the program *OriginPro* 2017.

**1‐(2‐Deoxy‐5‐*O*‐(4,4′‐dimethoxytriphenylmethyl)‐β‐D‐*erythro*‐pentofuranosyl)‐*N*** ^**4**^ **‐[(di‐n–butylamino)methylidene]‐*N*** ^**6**^ **‐formyl‐3‐(octa‐1,7‐diynyl)‐1*H*‐pyrazolo[3,4‐*d*]pyrimidin‐4,6‐diamine (7)**: To a suspension of 3^[13]^ (200 mg, 0.53 mmol) in MeOH (5 mL) *N,N*‐dibutylformamide dimethylacetal (549 mg, 2.70 mmol) was added. The mixture was stirred in an oil‐bath at 40 °C for 2 h. Then, the solvent was evaporated to dryness. The crude product was dissolved in anhydrous pyridine (5 mL). Then, the solution was treated with dimethoxytrityl chloride (600 mg, 1.77 mmol) at rt under stirring (45 min). The mixture was poured into 5 % aq. NaHCO_3_ solution (15 mL) and extracted with CH_2_Cl_2_ (2×20 mL). The organic layers were combined and dried over Na_2_SO_4_. After evaporation of the solvent, the residue was applied to FC (silica gel, column 2×8 cm, CH_2_Cl_2_/acetone, stepwise gradient from 98 : 2 to 95 : 5) to give 7 as a colorless foam (142 mg, 56 %). TLC (silica gel, CH_2_Cl_2_/acetone, 9 : 1): *R*_f_ 0.5; ^1^H NMR (400 MHz, [D_6_]DMSO, 26 °C): *δ*=10.69 (d, *J*=10.0 Hz, 1H; CHO), 9.54 (d, *J*=10.0 Hz, 1H; NH), 8.95 (s, 1H; N=CH), 7.34–7.25 (m, 2H; Ar−H), 7.25–7.10 (m, 7H; Ar−H), 6.81–6.69 (m, 4H; Ar−H), 6.47 (dd, *J*=6.9, 4.4 Hz, 1H; H‐1’), 5.30 (d, *J*=4.8 Hz, 1H; HO‐3‘), 4.49 (p, *J*=5.9 Hz, 1H; H‐3’), 3.92–3.84 (m, 1H; H‐4‘), 3.69 (d, *J=*2.4 Hz, 8H; 2×OCH_3_, 2×H‐5’), 3.49 (t, *J*=7.3 Hz, 2H; NCH_2_), 3.09–2.95 (m, 2H; NCH_2_), 2.84–2.73 (m, 2H; H‐2’_β_), 2.26 (dt, *J*=13.0, 6.8 Hz, 1H; H‐2’_α_), 2.18 (td*, J*=6.9, 2.7 Hz, 2H; CH_2_), 1.74–1.52 (m, 8H; 4×CH_2_), 1.40–1.20 (m, 6H; 3×CH_2_), 0.92 (t, *J=*7.3 Hz, 6H; 2×CH_3_). ^13^C NMR (101 MHz, [D_6_]DMSO, 26 °C): δ=164.4, 162.7, 158.2, 157.9, 155.8, 155.5, 145.1, 135.7, 129.6, 129.2, 127.7, 126.4, 113.0, 105.4, 93.1, 85.3, 85.2, 84.1, 83.6, 73.9, 71.4, 70.5, 64.1, 54.9, 51.1, 44.8, 37.9, 30.3, 28.8, 27.4, 27.2, 19.7, 19.2, 18.5, 17.3, 13.8, 13.6; UV (MeOH): *λ*_max_ (ϵ)=234 (35600), 260 (26900), 284 (17400), 326 (25600 dm^3^ mol^−1^ cm^−1^); HRMS (ESI‐TOF) m/z calcd for C_49_H_57_N_7_NaO_6_^+^ [*M*+Na]^+^: 862.4263; found: 862.4265.

**1‐(2‐Deoxy‐5‐*O*‐(4,4′‐dimethoxytriphenylmethyl)‐β‐D‐*erythro*‐pentofuranosyl)‐*N*** ^**4**^ **‐[(di‐n–butylamino)methylidene]‐*N*** ^**6**^ **‐formyl‐3‐(octa‐1,7‐diynyl)‐1*H*‐pyrazolo[3,4‐*d*]pyrimidin‐4,6‐diamine 3′‐(2‐cyanoethyl‐*N***, ***N*** **‐diisopropyl)phosphoramidite (8)**: To a solution of compound 7 (250 mg, 0.3 mmol) in dry CH_2_Cl_2_ (5 mL) were added, *N*,*N*‐diisopropylethylamine (101 μL, 0.59 mmol) and chloro(2‐cyanoethoxy)(diisopropylamino)phosphine (100 μL, 0.45 mmol) under stirring at rt. The stirring was continued for 10 min and the reaction mixture quenched with 5 mL CH_2_Cl_2_. An aq. solution of 5 % NaHCO_3_ (10 mL) was added, the layers separated, and the aqueous layer extracted with CH_2_Cl_2_ (2×20 mL). The combined organic extracts were dried over Na_2_SO_4_, filtered, evaporated and applied to FC (silica gel, column 2×6 cm, CH_2_Cl_2_/acetone, 95 : 5). Evaporation of the main zone yielded compound 8 as a colorless foam (231 mg, 74 %). TLC (silica gel, CH_2_Cl_2_/acetone, 98 : 2): *R*_f_ 0.7, 0.6. ^31^P NMR (121 MHz, CDCl_3_, 26 °C): *δ=*148.3, 148.4. HRMS (ESI‐TOF) m/z calcd for C_58_H_74_N_9_NaO_7_P^+^ [*M*+Na]^+^: 1062.5341; found: 1062.5345.

**2,2‐Bis((4‐(pyren‐1‐yl)‐1*H*‐1,2,3‐triazol‐1‐yl)methyl)propane‐1,3‐diol** (**5)**: 2,2‐Bis(bromomethyl)propane‐1,3‐diol **11** ^[15]^ (0.4 g, 1.53 mmol) and NaN_3_ (0.4 g, 6.15 mmol) were dissolved in 5 mL DMF. The solution was stirred at 110 °C overnight and filtered. After evaporation of the solvent, the resulting solid was dissolved in 10 mL CH_2_Cl_2_. The filtrate was obtained and evaporated, dissolved in 20 mL diethyl ether and washed with 10 mL aq. NaCl. The organic phase was dried over anhydrous Na_2_SO_4_ and concentrated. Compound **12** ^[15]^ was obtained as a colorless oil and used in the next step without further purification.

Compound **5** was dissolved in dry THF (21 mL) and degassed for 5 min with N_2_. Then, 1‐ethynylpyrene **13** (0.83 g, 3.66 mmol) was added, and stirring and degassing were continued for another 5 min. Then, a freshly prepared 1 M solution of sodium ascorbate (0.6 mL, 0.61 mmol) in water and a 7.5 % copper(II) sulfate solution in water (0.55 mL, 0.15 mmol) were added. The final ratio of THF to H_2_O in the reaction mixture was maintained as 3 : 1. Finally, *N*,*N*‐diisopropylethylamine (DIPEA) was added to the reaction mixture (0.47 g, 3.66 mmol). The reaction mixture was refluxed at 80 °C overnight with stirring, evaporated and partitioned between H_2_O and CH_2_Cl_2_. The organic layer was washed with water followed by brine, dried over Na_2_SO_4_, and concentrated. Then, the residue was purified by FC (silica gel, column 3×10 cm, CH_2_Cl_2_/MeOH, 95 : 5) to give **13** as a colorless solid (0.5 g, 51 %, 2 steps). TLC (CH_2_Cl_2_/MeOH, 95 : 5): *R*_f_ 0.38; ^1^H NMR (600 MHz, [D_6_]DMSO, 26 °C): *δ*=8.90 (d, *J*=9.3 Hz, 2H; pyrene‐H), 8.77 (s, 2H; triazole‐H), 8.37 (d, *J*=1.4 Hz, 4H; pyrene‐H), 8.32 (ddd, *J=*14.1, 7.7, 1.1 Hz, 4H; pyrene‐H), 8.27–8.19 (m, 6H; pyrene‐H), 8.10 (t, *J*=7.6 Hz, 2H; pyrene‐H), 4.77 (s, 4H; 2×OCH_2_), 3.49 (d, *J*=4.5 Hz, 4H; 2×NCH_2_); ^13^C NMR (151 MHz, [D_6_]DMSO, 26 °C): *δ*=145.9, 130.9, 130.6, 130.4, 128.0, 127.7, 127.5, 127.3, 126.4, 126.4, 125.5, 125.3, 125.2, 125.1, 124.9, 124.3, 123.9, 60.3, 50.1, 45.7; UV (MeOH): *λ*_max_ (ϵ) 244 (43400), 268 (21400), 278 (32300), 345 (26000 dm^3^ mol^−1^ cm^−1^). HRMS (ESI‐TOF) m/z calcd for C_41_H_30_N_6_NaO_2_^+^ [*M*+Na]^+^: 661.2328; found: 661.2327.

**3‐(4,4’‐Dimethoxytrityl)‐2,2‐bis((4‐(pyren‐1‐yl)‐1*H*‐1,2,3‐triazol‐1‐yl)methyl)propan‐1‐ol (14)**: Compound **5** (100 mg, 0.156 mmol) was dried by repeated co‐evaporation with dry pyridine (3×3 mL) and suspended in dry pyridine (3 mL). Then, DMT−Cl (64 mg, 0.1875 mmol) was added, and the mixture was stirred for 4 h at rt. Then, the mixture was diluted with CH_2_Cl_2_ (10 mL) and a 5 % aqueous solution of NaHCO_3_ (20 mL) was added. The organic phase was dried over Na_2_SO_4_ and evaporated, and the residue was separated by FC (silica gel, column 10×3 cm, CH_2_Cl_2_/MeOH, 98 : 2) to give **14** as a colorless solid (77 mg, 52 %). TLC (CH_2_Cl_2_/MeOH, 95 : 5): *R*_f_ 0.67; ^1^H NMR (600 MHz, [D_6_]DMSO, 26 °C): *δ*=8.81 (d, *J=*9.3 Hz, 2H; pyrene‐H), 8.51 (s, 2H; triazole‐H), 8.40–8.30 (m, 6H; pyrene‐H), 8.28–8.19 (m, 8H; pyrene‐H), 8.11 (t, *J=*7.6 Hz, 2H; pyrene‐H), 7.39 (ddt, *J*=6.0, 3.3, 1.6 Hz, 2H; Ar−H), 7.28–7.20 (m, 6H; Ar−H), 7.18–7.10 (m, 1H; Ar−H), 6.76–6.67 (m, 4H; Ar−H), 4.85 (d, *J*=14.3 Hz, 2H; OCH_2_), 4.79 (d, *J*=14.3 Hz, 2H; OCH_2_), 3.56 (d, *J*=4.4 Hz, 2H; NCH_2_), 3.45 (s, 6H; 2 × OCH_3_), 3.23 (s, 2H; NCH_2_); ^13^C NMR (151 MHz, [D_6_]DMSO, 26 °C): *δ*=158.0, 149.6, 145.9, 144.5, 136.1, 134.9, 130.9, 130.6, 130.4, 129.9, 127.9, 127.8, 127.7, 127.5, 127.3, 127.1, 126.7, 126.5, 126.1, 125.5, 125.2, 125.2, 125.0, 124.9, 124.3, 123.9, 123.9, 113.0, 86.0, 62.3, 60.9, 54.7, 50.8, 45.2; UV (MeOH): *λ*_max_ (ϵ) 244 (101400), 269 (47300), 278 (69700), 348 (55300 dm^3^ mol^−1^ cm^−1^). HRMS (ESI‐TOF) m/z calcd for C_62_H_48_N_6_NaO_4_^+^ [*M*+Na]^+:^963.3635; found: 963.3638.

**3‐(4,4’‐Dimethoxytrityl)‐2,2‐bis((4‐(pyren‐1‐yl)‐1*H*‐1,2,3‐triazol‐1‐yl)methyl)propyl 3’‐(2‐cyanoethyl‐*N***, ***N*** **‐diisopropyl)phosphoramidite (15)**: To a solution of compound **14** (120 mg, 0.128 mmol), chloro(2‐cyanoethoxy)(diisopropylamino)phosphine (45 μL, 0.191 mmol) and anhydrous iPr_2_EtN (45 μL, 0.255 mmol) were added at rt in anhydrous CH_2_Cl_2_ (5 mL). After stirring for 30 min, the mixture was diluted with CH_2_Cl_2_ (5 mL) and the reaction was quenched by adding a 5 % aq. solution of NaHCO_3_ (20 mL). Then, the aq. layer was extracted with CH_2_Cl_2_ (60 mL) and the combined organic layer was dried (Na_2_SO_4_) and evaporated. The residual colorless oil was applied to FC (silica gel, column 10×3 cm, CH_2_Cl_2_/acetone, 97 : 3). From the main zone, compound **15** was obtained as a colorless foam (98 mg, 70 %). TLC (silica gel, CH_2_Cl_2_/acetone, 97 : 3): *R*_f_= 0.67. ^31^P NMR (121 MHz, CDCl_3_, 26 °C): *δ=*148.9. HRMS (ESI‐TOF) m/z calcd for C_71_H_65_N_8_NaO_5_P^+^ [*M*+Na]^+^: 1163.4713; found: 1163.4717.

**General procedure for Huisgen‐Meldal‐Sharpless [3+2] cycloaddition performed on oligonucleotides containing stabilizer 3 in aqueous solution with 1‐azidomethylpyrene to obtain sensor 4**: To a ss‐oligonucleotide (5 A_260_ units) were added CuSO_4_•TBTA (1 : 2) ligand complex (50 μL of a 20 mM stock solution in H_2_O/DMSO/t‐BuOH, 4 : 3 : 1), tris(carboxyethyl)‐phosphine (TCEP, 50 μL of a 20 mM stock solution in water), NaHCO_3_ (50 μL, 200 mM stock solution in water), 1‐azidomethylpyrene (100 μL, 20 mM stock solution in H_2_O/dioxane/DMSO, 1 : 1 : 1), and DMSO (30 μL), and the reaction mixture was stirred at room temperature for 12 h. The reaction mixture was concentrated in a speed‐vac and dissolved in 500 μL bi‐distilled water and centrifuged for 30 min at 14 000 rpm. The supernatant solution was collected and further purified by reversed‐phase HPLC with the gradient 0–3 min 10–15 % B in A, 3–15 min 15–50 % B in A, 15–20 min 50–10 % B in A, flow rate 0.7 cm^3^ min^−1^. The molecular masses of the oligonucleotides were determined by MALDI‐TOF spectra (Table 1).

## Conflict of interest

The authors declare no conflict of interest.

1

## Supporting information

As a service to our authors and readers, this journal provides supporting information supplied by the authors. Such materials are peer reviewed and may be re‐organized for online delivery, but are not copy‐edited or typeset. Technical support issues arising from supporting information (other than missing files) should be addressed to the authors.

Supporting Information

## Data Availability

The data that support the findings of this study are available from the corresponding author upon reasonable request.
