# Supplementary material for: DNA Strand Displacement with Base Pair Stabilizers: Purine‐2,6‐Diamine and 8‐Aza‐7‐Bromo‐7‐Deazapurine‐2,6‐Diamine Oligonucleotides Invade Canonical DNA and New Fluorescent Pyrene Click Sensors Monitor the Reaction
Source: Chemistry. 2022 Nov 15;28(72):e202202412. doi: 10.1002/chem.202202412 (PMC10100337; doi:10.1002/chem.202202412)
Supplement: Supplementary file 1 — Supporting Information [file CHEM-28-0-s001.pdf]

Chemistry–A European Journal

Supporting Information

DNA Strand Displacement with Base Pair Stabilizers: Purine-2,6-Diamine and 8-Aza-7-Bromo-7-Deazapurine-2,6-Diamine Oligonucleotides Invade Canonical DNA and New Fluorescent Pyrene Click Sensors Monitor the Reaction

Aigui Zhang, Dasharath Kondhare, Peter Leonard, and Frank Seela*

Table of Contents

Figure S1. Modified phosphoramidites used in this study S2

Table S1. ^{13}C NMR chemical shifts of 7-octadiynyl-8-aza-7-deazapurine-2,6-diamine derivatives S2

Table S2. ^{13}C NMR chemical shifts of bis-pyrene derivatives **5** and **15** S2

Table S3. T_m values of oligonucleotide duplexes before and after addition of invader strands and duplexes containing 2,6-diamino nucleosides S3-4

References S4

Table S4. T_m values and thermodynamic data of antiparallel stranded duplexes S5

Table S5. T_m values and thermodynamic data of oligonucleotide duplexes after addition of the invader strand S6-7

Figure S2. Fluorescence of ODN-**10** and ODN-**12** and corresponding duplexes S8

Figure S3. Reversed-phase HPLC elution profiles of purified oligonucleotides S9-10

Figure S4. Thermal denaturation curves of oligonucleotide duplexes S11-14

NMR Measurements S14

Figures S5-S31. NMR spectra of synthesized compounds S15-28

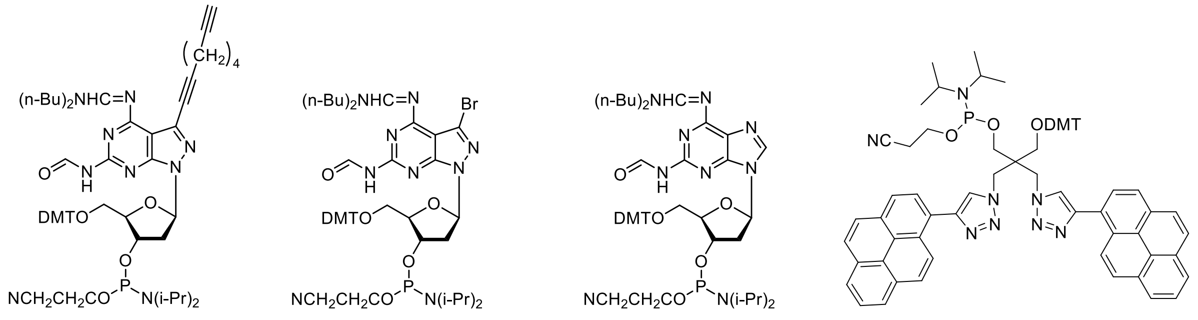

8 9[1] 10[2] 15

Figure S1. Modified phosphoramidites used in this study.

Table S1. ^{13}C NMR chemical shifts of the 7-octadiynylated 8-aza-7-deazapurine-2,6-diamine derivative **6**.[a]

Cpd.	C(2)[b,d]<br>C(2)[c]	C(4)[d]<br>C(7*a*)	C(5)<br>C(3*a*)	C(6)[d]<br>C(4)	C(7)<br>C(3)	C≡C	CH3<br>CH2		C=O<br>CH=N	OCH3	C(1')	C(2')	C(3')	C(4')	C(5')
**6**	157.9	158.2	105.6	162.7	126.4	70.5<br>73.9<br>84.1<br>93.1	13.6<br>13.8<br>17.3<br>18.5<br>19.2<br>19.7	27.2<br>27.4<br>28.8<br>30.3<br>44.8<br>51.1	164.4<br>155.5	54.9	83.6	37.9	71.4	85.3	64.1

[a] Measured in [D6]DMSO at 298 K. [b] Purine numbering. [c] Systematic numbering. [d] Tentative.

Table S2. ^{13}C NMR chemical shifts of bis-pyrene derivatives **5** and **14**.[a]

Cpd.	CH2	Pyrene-C	Triazole-C	OCH3
**5**	50.1<br>60.3	123.9-130.9	130.4<br>145.9	-
**14**	50.8<br>60.9<br>62.3	123.9-158.0	130.4<br>145.9	54.7

[a] Measured in [D6]DMSO at 298 K.

Table S3. T_m values of oligonucleotide duplexes before and after addition of the invader strand and duplexes containing 2,6-diamino nucleosides **1-4** or bis-pyrene sensor **5**.[a] (Table S3 is identical to Table 2 in the manuscript but data are listed in a different arrangement.)

**Displacement Input**<br>**Invader added to the Duplex**	T_m[b]<br>[°C]	**Displacement Output**<br>**New duplex and Released Strand**	T_m[c]<br>[°C]
**Substrate Duplex**<br>5'-d(TAG GTC AAT ACT) (ODN-**1**)<br>3'-d(ATC CAG TTA TGA) (ODN-**2**)<br>**Invader Input**<br>3'-d(ATC CAG TT**1** TGA) (ODN-**3**)	47	5'-d(TAG GTC AAT ACT) (ODN-**1**)<br>3'-d(ATC CAG TT**1** TGA) (ODN-**3**)<br>+<br>3'-d(ATC CAG TTA TGA) (ODN-**2**)	52
**Substrate Duplex**<br>5'-d(TAG GTC AAT ACT) (ODN-**1**)<br>3'-d(ATC CAG TTA TGA) (ODN-**2**)<br>**Invader Input**<br>3'-d(ATC C**1**G TT**1** TG**1**) (ODN-**4**)	47	5'-d(TAG GTC AAT ACT) (ODN-**1**)<br>3'-d(ATC C**1**G TT**1** TG**1**) (ODN-**4**)<br>+<br>3'-d(ATC CAG TTA TGA) (ODN-**2**)	52
**Substrate Duplex**<br>5'-d(TAG GTC AAT ACT) (ODN-**1**)<br>3'-d(ATC CAG TTA TGA) (ODN-**2**)<br>**Invader Input**<br>3'-d(ATC CAG TT**2** TGA) (ODN-**5**)	47	5'-d(TAG GTC AAT ACT) (ODN-**1**)<br>3'-d(ATC CAG TT**2** TGA) (ODN-**5**)<br>+<br>3'-d(ATC CAG TTA TGA) (ODN-**2**)	54
**Substrate Duplex**<br>5'-d(TAG GTC AAT ACT) (ODN-**1**)<br>3'-d(ATC CAG TTA TGA) (ODN-**2**)<br>**Invader Input**<br>3'-d(ATC C**2**G TT**2** TG**2**) (ODN-**6**)	47	5'-d(TAG GTC AAT ACT) (ODN-**1**)<br>3'-d(ATC C**2**G TT**2** TG**2**) (ODN-**6**)<br>+<br>3'-d(ATC CAG TTA TGA) (ODN-**2**)	60
**Substrate Duplex**<br>5'-d(TAG GTC AAT ACT) (ODN-**1**)<br>3'-d(ATC CAG TTA TGA) (ODN-**2**)<br>**Invader Input**<br>3'-d(ATC C**2**G TT**3** TG**2**) (ODN-**7**)	47	5'-d(TAG GTC AAT ACT) (ODN-**1**)<br>3'-d(ATC C**2**G TT**3** TG**2**) (ODN-**7**)<br>+<br>3'-d(ATC CAG TTA TGA) (ODN-**2**)	58
**Substrate Duplex**<br>5'-d(TAG GTC AAT ACT) (ODN-**1**)<br>3'-d(ATC CAG TTA TGA) (ODN-**2**)<br>**Invader Input**<br>3'-d(ATC C**2**G TT**4** TG**2**) (ODN-**7**)	47	5'-d(TAG GTC AAT ACT) (ODN-**1**)<br>3'-d(ATC C**2**G TT**4** TG**2**) (ODN-**7**)<br>+<br>3'-d(ATC CAG TTA TGA) (ODN-**2**)	66
**Substrate Duplex**<br>5'-d(TAG GTC AAT ACT) (ODN-**1**)<br>3'-d(ATC CAG TTA TGA) (ODN-**2**)<br>**Invader Input**<br>3'-d(ATC C**2**G TT**3** TGA) (ODN-**9**)	47	5'-d(TAG GTC AAT ACT) (ODN-**1**)<br>3'-d(ATC C**2**G TT**3** TGA) (ODN-**9**)<br>+<br>3'-d(ATC CAG TTA TGA) (ODN-**2**)	56
**Substrate Duplex**<br>5'-d(TAG GTC AAT ACT) (ODN-**1**)<br>3'-d(ATC CAG TTA TGA) (ODN-**2**)<br>**Invader Input**<br>3'-d(ATC C**2**G TT**4** TGA) (ODN-**9**)	47	5'-d(TAG GTC AAT ACT) (ODN-**1**)<br>3'-d(ATC C**2**G TT**4** TGA) (ODN-**9**)<br>+<br>3'-d(ATC CAG TTA TGA) (ODN-**2**)	67
**Substrate Duplex**<br>5'-d(TAG GTC AAT ACT) (ODN-**1**)<br>3'-d(ATC CAG TTA TGA) (ODN-**2**)<br>**Invader Input**<br>3'-d(ATC CAG TT**3** TG**2**) (ODN-**11**)	47	5'-d(TAG GTC AAT ACT) (ODN-**1**)<br>3'-d(ATC CAG TT**3** TG**2**) (ODN-**11**)<br>+<br>3'-d(ATC CAG TTA TGA) (ODN-**2**)	54
**Substrate Duplex**<br>5'-d(TAG GTC AAT ACT) (ODN-**1**)<br>3'-d(ATC CAG TTA TGA) (ODN-**2**)<br>**Invader Input**<br>3'-d(ATC CAG TT**4** TG**2**) (ODN-**11**)	47	5'-d(TAG GTC AAT ACT) (ODN-**1**)<br>3'-d(ATC CAG TT**4** TG**2**) (ODN-**11**)<br>+<br>3'-d(ATC CAG TTA TGA) (ODN-**2**)	63
**Substrate Duplex**<br>5'-d(TAG GTC AAT ACT) (ODN-1)<br>3'-d(ATC CAG TTA TGA**5**) (ODN-**13**)<br>**Invader Input**	50	5'-d(TAG GTC AAT ACT) (ODN-**1**)<br>3'-d(ATC C**1**G TT**1** TG**1**) (ODN-**4**)<br>+<br>3'-d(ATC CAG TTA TGA**5**) (ODN-**13**)	53

3’-d(ATC C1G TT1 TG1) (ODN-**4**)			
**Substrate Duplex**<br>5’-d(TAG GTC AAT ACT) (ODN-**1**)<br>3’-d(ATC CAG TTA TGA5) (ODN-**13**)<br>**Invader Input**<br>3’-d(ATC C2G TT2 TG2) (ODN-**6**)	50	5’-d(TAG GTC AAT ACT) (ODN-**1**)<br>3’-d(ATC C2G TT2 TG2) (ODN-**6**)<br>+<br>3’-d(ATC CAG TTA TGA5) (ODN-**13**)	59
**Duplexes Only**	T_m[b] [°C]	**Duplexes Only**	T_m[b] [°C]
5’-d(TAG GTC AAT ACT) (ODN-**1**)<br>3’-d(ATC CAG TTA TGA) (ODN-**2**)	47	5’-d(TAG GTC AAT ACT) (ODN-**1**)<br>3’-d(ATC CAG TTA TGA5) (ODN-**13**)	50
5’-d(TAG GTC AAT ACT) (ODN-**1**)<br>3’-d(ATC CAG TT1 TGA) (ODN-**3**)	50	5’-d(TAG GTC AAT ACT) (ODN-**1**)<br>3’-d(ATC CAG TT2 TGA) (ODN-**5**)	54
5’-d(TAG GTC AAT ACT) (ODN-**1**)<br>3’-d(ATC C1G TT1 TG1) (ODN-**4**)	52	5’-d(TAG GTC AAT ACT) (ODN-**1**)<br>3’-d(ATC C2G TT2 TG2) (ODN-**6**)	59
5’-d(TAG GTC AAT ACT) (ODN-**1**)<br>3’-d(ATC C2G TT3 TG2) (ODN-**7**)	58	5’-d(TAG GTC AAT ACT) (ODN-**1**)<br>3’-d(ATC C2G TT4 TG2) (ODN-**7**)	66
5’-d(TAG GTC AAT ACT) (ODN-**1**)<br>3’-d(ATC C2G TT3 TGA) (ODN-**9**)	57	5’-d(TAG GTC AAT ACT) (ODN-**1**)<br>3’-d(ATC C2G TT4 TGA) (ODN-**9**)	67
5’-d(TAG GTC AAT ACT) (ODN-**1**)<br>3’-d(ATC CAG TT3 TG2) (ODN-**11**)	54	5’-d(TAG GTC AAT ACT) (ODN-**1**)<br>3’-d(ATC CAG TT4 TG2) (ODN-**11**)	62

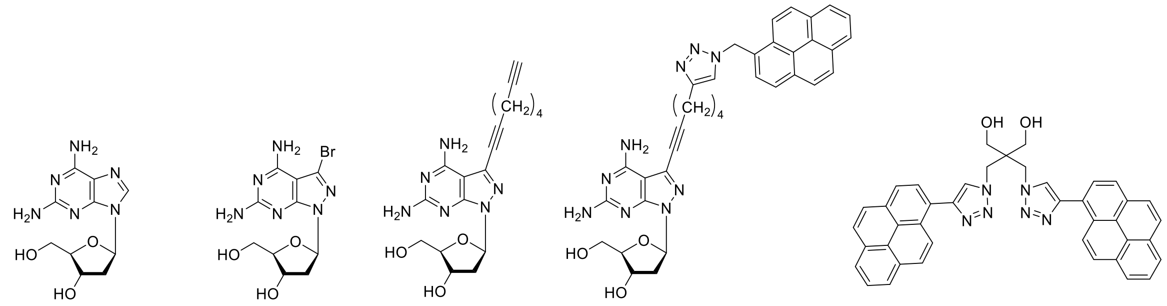

1 2 3 4 5

[a] Measured at 260 nm at a concentration of 5 µM + 5 µM single strand at a heating rate of 1.0°C/min in 100 mM NaCl, 10 mM $MgCl_2$, and 10 mM Na-cacodylate (pH 7.0). [b] T_m values were calculated from the heating curves using the program *Meltwin 3.0*[3]. [c] T_m values were calculated from the heating curves after adding the corresponding invader strand with 5 µM concentration using the program *Meltwin 3.0*.[3] The standard deviation for the T_m values is ± 0.5 °C.

References

[1] F. Seela, G. Becher, *Nucleic Acids Res*. **2001**, *29*, 2069-2078; b) G. Becher, J. He, F. Seela, *Helv. Chim. Acta* **2001**, *84*, 1048-1065.

[2] Y. Chai, D. Kondhare, A. Zhang, P. Leonard, F. Seela, *Chem. Eur. J.* **2021**, *27*, 2093-2103.

[3] J. A. McDowell, D. H. Turner, *Biochemistry* **1996**, *35*, 14077-14089.

Table S4. T_m values and thermodynamic data for antiparallel stranded duplexes.[a]

Homochiral Duplexes	T_m[b] [°C]	$\Delta H°$ [kcal/mol]	$\Delta S°$ [cal/K mol]	$\Delta G°_{310}$ [kcal/mol]
5'-d(TAG GTC AAT ACT) (ODN-**1**)<br>3'-d(ATC CAG TTA TGA) (ODN-**2**)	47	-82	-228	-11.0
5'-d(TAG GTC AAT ACT) (ODN-**1**)<br>3'-d(ATC CAG TT**1** TGA) (ODN-**3**)	50	-92	-259	-12.1
5'-d(TAG GTC AAT ACT) (ODN-**1**)<br>3'-d(ATC CAG TT**2** TGA) (ODN-**5**)	54	-88	-242	-12.9
5'-d(TAG GTC AAT ACT) (ODN-**1**)<br>3'-d(ATC C**1**G TT**1** TG**1**) (ODN-**4**)	52	-88	-244	-12.4
5'-d(TAG GTC AAT ACT) (ODN-**1**)<br>3'-d(ATC C**2**G TT**2** TG**2**) (ODN-**6**)	59	-90	-243	-14.4
5'-d(TAG GTC AAT ACT) (ODN-**1**)<br>3'-d(ATC C**2**G TT**3** TG**2**) (ODN-**7**)	58	-95	-259	-14.4
5'-d(TAG GTC AAT ACT) (ODN-**1**)<br>3'-d(ATC C**2**G TT**4** TG**2**) (ODN-**7**)	66	-85	-225	-15.4
5'-d(TAG GTC AAT ACT) (ODN-**1**)<br>3'-d(ATC C**2**G TT**3** TGA) (ODN-9)	57	-87	-236	-13.5
5'-d(TAG GTC AAT ACT) (ODN-**1**)<br>3'-d(ATC C**2**G TT**4** TGA) (ODN-**9**)	67	-87	-230	-16.0
5'-d(TAG GTC AAT ACT) (ODN-**1**)<br>3'-d(ATC CAG TT**3** TG**2**) (ODN-**11**)	54	-84	-232	-12.7
5'-d(TAG GTC AAT ACT) (ODN-**1**)<br>3'-d(ATC CAG TT**4** TG**2**) (ODN-**11**)	62	-82	-216	-14.6
5'-d(TAG GTC AAT ACT) (ODN-**1**)<br>3'-d(ATC CAG TTA TGA**5**) (ODN-**13**)	50	-81	-225	-11.7

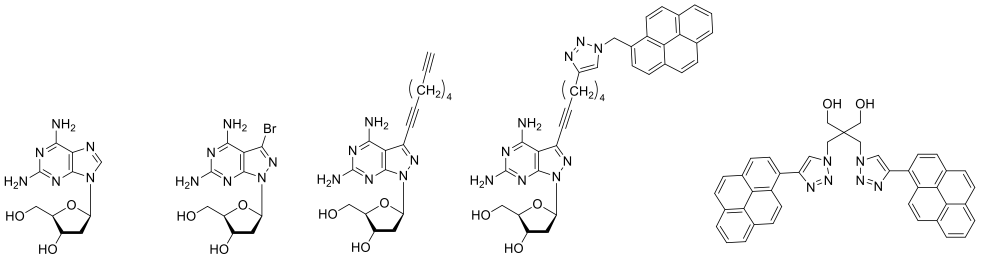

1 2 3 4 5

[a] Measured at 260 nm at a concentration of 5 µM + 5 µM single strand at a heating rate of 1.0°C/min in 100 mM NaCl, 10 mM $MgCl_2$, and 10 mM Na-cacodylate (pH 7.0). [b] T_m values were calculated from the heating curves using the program *Meltwin 3.0*.[3] The standard deviation for the T_m values is ± 0.5 °C.

Table S5. T_m values and thermodynamic data oligonucleotide duplexes after addition of the invader strand.[a]

Displacement Input<br>Invader added to the Duplex	T_m[b] [°C]	ΔH° [kcal/mol]	ΔS° [cal/K mol]	ΔG°$_{310}$ [kcal/mol]
5'-d(TAG GTC AAT ACT) (ODN-**1**)<br>3'-d(ATC CAG TT**1** TGA) (ODN-**3**)<br>+<br>3'-d(ATC CAG TTA TGA) (ODN-**2**)	52	-99	-276	-13.0
5'-d(TAG GTC AAT ACT) (ODN-**1**)<br>3'-d(ATC C**1**G TT**1** TG**1**) (ODN-**4**)<br>+<br>3'-d(ATC CAG TTA TGA) (ODN-**2**)	52	-98	-273	-13.0
5'-d(TAG GTC AAT ACT) (ODN-**1**)<br>3'-d(ATC CAG TT**2** TGA) (ODN-**5**)<br>+<br>3'-d(ATC CAG TTA TGA) (ODN-**2**)	54	-99	-275	-13.4
5'-d(TAG GTC AAT ACT) (ODN-**1**)<br>3'-d(ATC C**2**G TT**2** TG**2**) (ODN-**6**)<br>+<br>3'-d(ATC CAG TTA TGA) (ODN-**2**)	60	-96	-262	-14.9
5'-d(TAG GTC AAT ACT) (ODN-**1**)<br>3'-d(ATC C**2**G TT**3** TG**2**) (ODN-**7**)<br>+<br>3'-d(ATC CAG TTA TGA) (ODN-**2**)	58	-92	-252	-14.4
5'-d(TAG GTC AAT ACT) (ODN-**1**)<br>3'-d(ATC C**2**G TT**4** TG**2**) (ODN-**7**)<br>+<br>3'-d(ATC CAG TTA TGA) (ODN-**2**)	66	-80	-207	-15.3
5'-d(TAG GTC AAT ACT) (ODN-**1**)<br>3'-d(ATC C**2**G TT**3** TGA) (ODN-**9**)<br>+<br>3'-d(ATC CAG TTA TGA) (ODN-**2**)	56	-84	-228	-13.2
5'-d(TAG GTC AAT ACT) (ODN-**1**)<br>3'-d(ATC C**2**G TT**4** TGA) (ODN-**9**)<br>+<br>3'-d(ATC CAG TTA TGA) (ODN-**2**)	67	-83	-217	-15.6
5'-d(TAG GTC AAT ACT) (ODN-**1**)<br>3'-d(ATC CAG TT**3** TG**2**) (ODN-**11**)<br>+<br>3'-d(ATC CAG TTA TGA) (ODN-**2**)	54	-88	-242	-12.7
5'-d(TAG GTC AAT ACT) (ODN-**1**)<br>3'-d(ATC CAG TT**4** TG**2**) (ODN-**11**)<br>+<br>3'-d(ATC CAG TTA TGA) (ODN-**2**)	63	-75	-196	-14.1
5'-d(TAG GTC AAT ACT) (ODN-**1**)<br>3'-d(ATC C**1**G TT**1** TG**1**) (ODN-**4**)<br>+<br>3'-d(ATC CAG TTA TGA**5**) (ODN-**13**)	53	-95	-265	-13.1
5'-d(TAG GTC AAT ACT) (ODN-**1**)<br>3'-d(ATC C**2**G TT**2** TG**2**) (ODN-**6**)<br>+<br>3'-d(ATC CAG TTA TGA**5**) (ODN-**13**)	59	-85	-229	-13.9

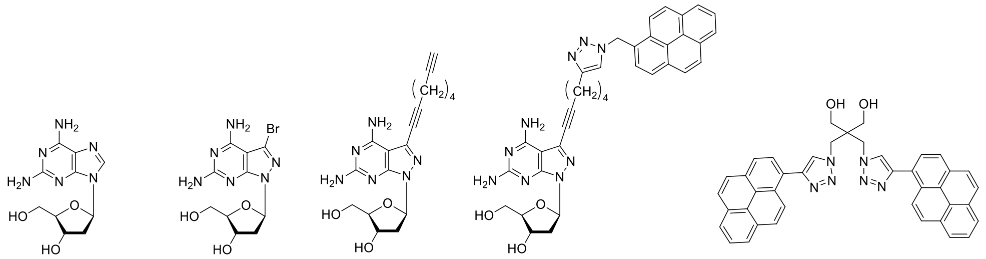

1 2 3 4 5

[a] Measured at 260 nm at a concentration of 5 µM + 5 µM single strand at a heating rate of 1.0°C/min in 100 mM NaCl, 10 mM $MgCl_2$, and 10 mM Na-cacodylate (pH 7.0). [b] T_m values were calculated from the heating curves. [c] T_m values were calculated from the heating curves after adding the corresponding invader strand with 5 µM concentration using the program *Meltwin 3.0*.[3] The standard deviation for the T_m values is ± 0.5 °C.

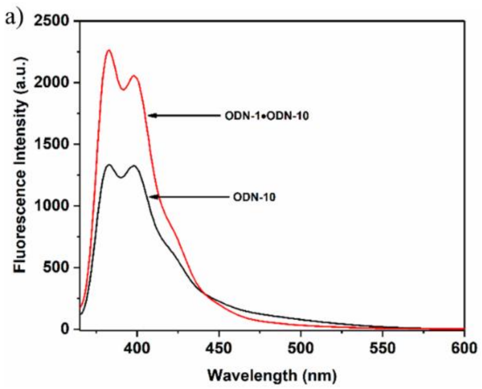

3’-d(ATC C **2**G TT**4** TGA) (ODN-**10**)

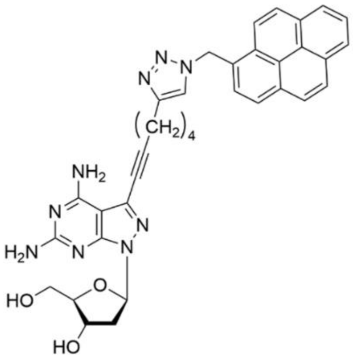

5’-d(TAG GTC AAT ACT) (ODN-**1**)
3’-d(ATC C **2**G TT**4** TGA) (ODN-**10**)

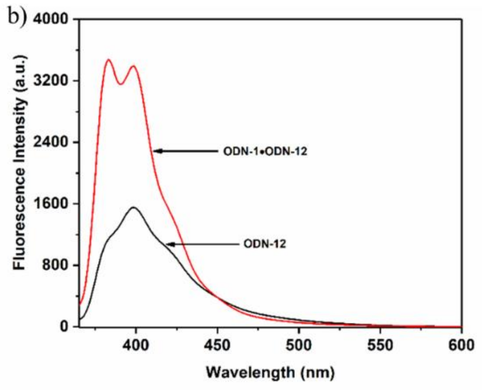

3’-d(ATC CAG TT**4** TG **2**) (ODN-**12**)

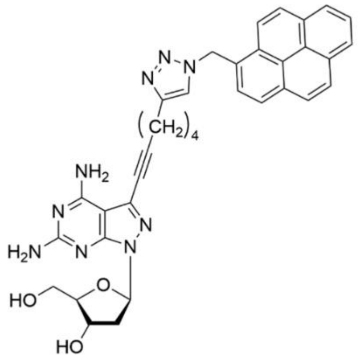

5’-d(TAG GTC AAT ACT) (ODN-**1**)
3’-d(ATC CAG TT**4** TG **2**) (ODN-**12**)

Figure S2. Fluorescence of a) oligonucleotide ODN-**10** and the corresponding duplex 5’-d(TAG GTC AAT ACT) (ODN-**1**) • 3’-d(ATC C**2**G TT**4** TGA) (ODN-**10**); b) oligonucleotide ODN-**12** and the corresponding duplex 5’-d(TAG GTC AAT ACT) (ODN-**1**) • 3’-d(ATC CAG TT**4** TG**2**) (ODN-**12**). The excitation and emission bandpass was 10 nm. The exciation wavelength was 345 nm.

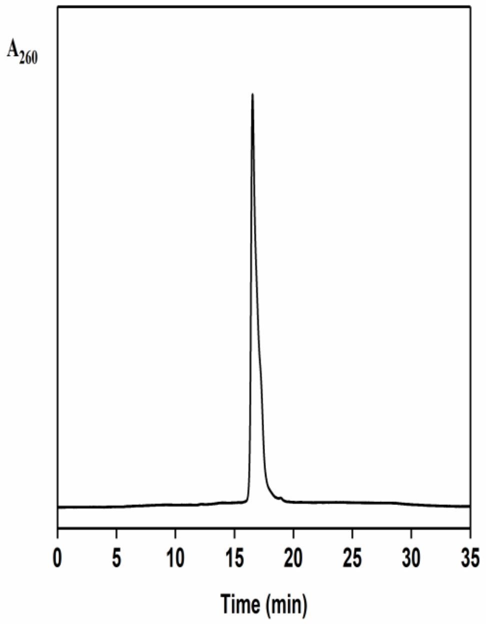

5’-d(**2**GT**3**TTG**2**CCTA) (ODN-**7**)

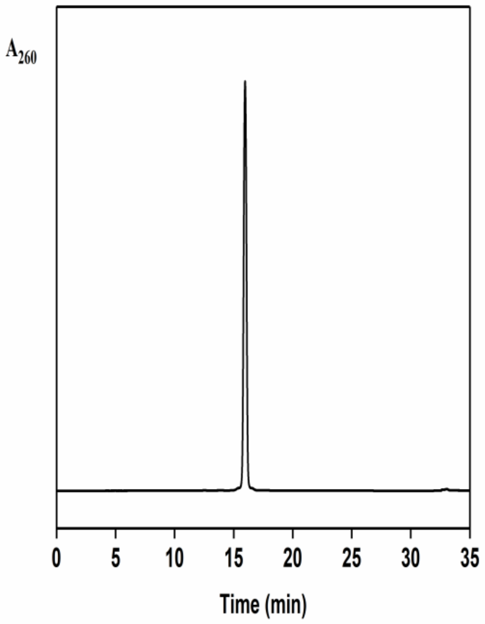

5’-d(AGT**3**TTG**2**CCTA) (ODN-**9**)

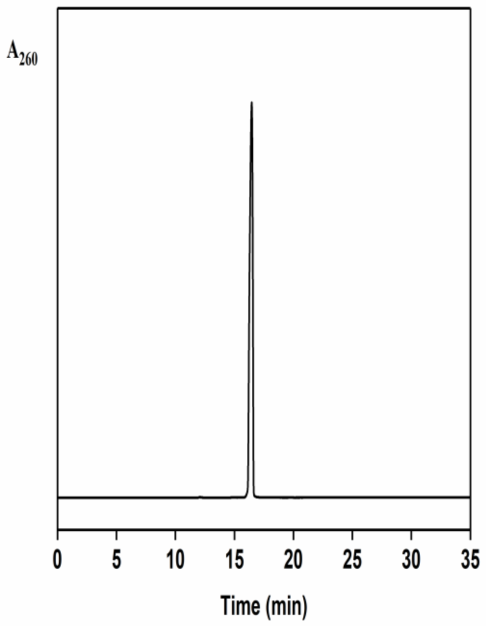

5’-d(**2**GT**3**TTGACCTA) (ODN-**11**)

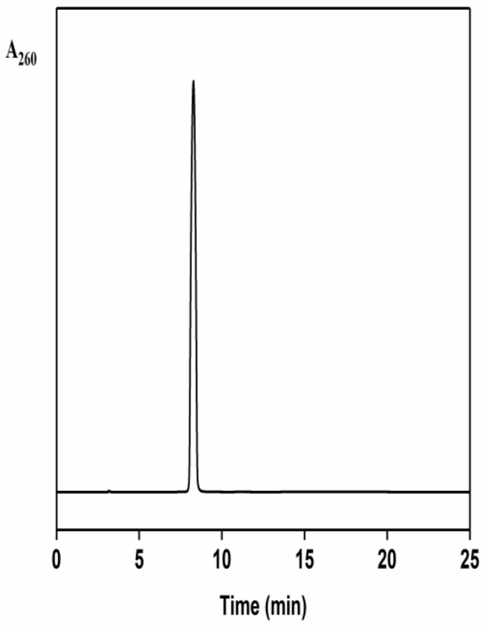

5’-d(**2**GT**4**TTG**2**CCTA) (ODN-**8**)

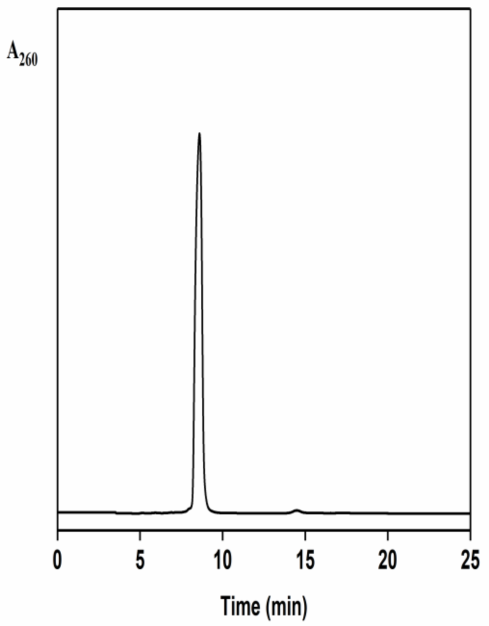

5’-d(AGT**4**TTG**2**CCTA) (ODN-**10**)

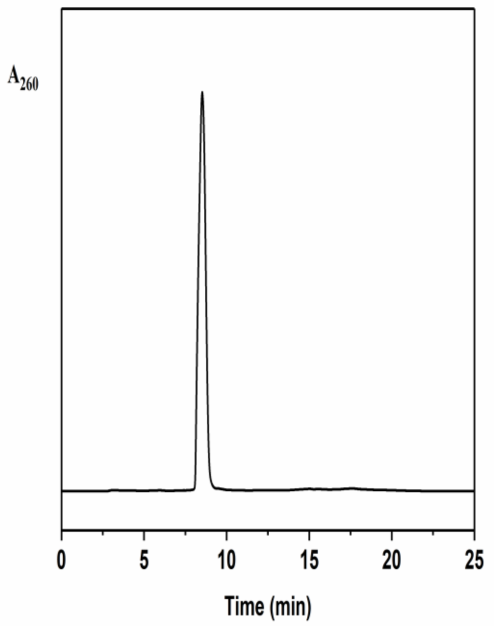

5’-d(**2**GT**4**TTGACCTA) (ODN-**12**)

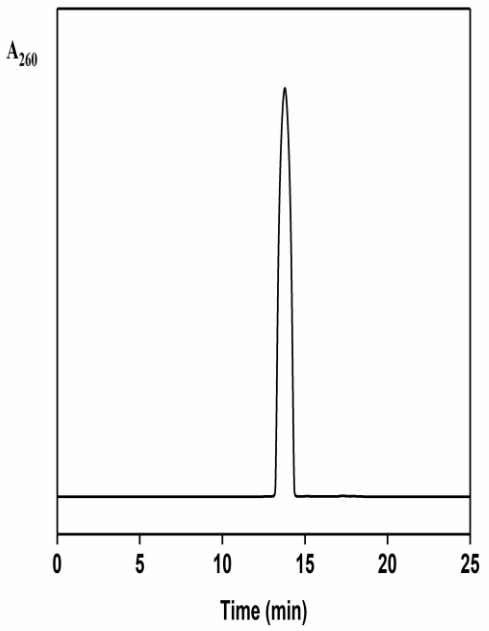

5’-d(**5**AGTATTGACCTA) (ODN-**13**)

Figure S3. Reversed-phase (RP-18) HPLC elution profiles of purified oligonucleotides monitored at 260 nm. X-axis refers to retention time (min); Y-axis refers to UV absorbance at 260 nm, measured in mV. For elution, the following system was used: (A) MeCN, (B) 0.1 M (Et_3NH)OAc (pH 7.0)/MeCN, 95:5; gradient: 0-20 min 0-20% A in B; 20-25 min, 20% A in B; flow rate 0.7 mL/min.

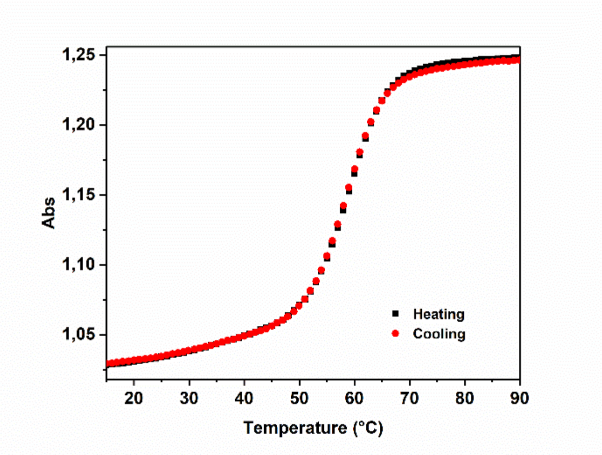

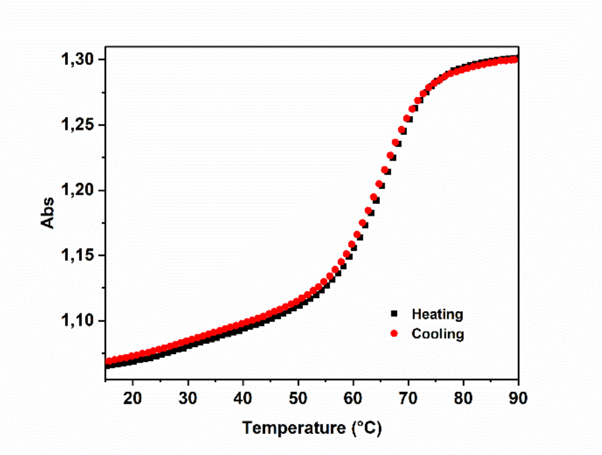

5’-d(TAG GTC AAT ACT) (ODN-**1**)
3’-d(ATC C2G TT3 TG2) (ODN-**7**)

5’-d(TAG GTC AAT ACT) (ODN-**1**)
3’-d(ATC C2G TT4 TG2) (ODN-**8**)

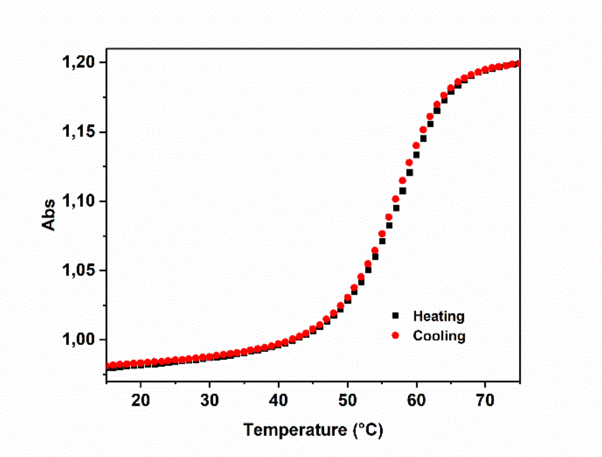

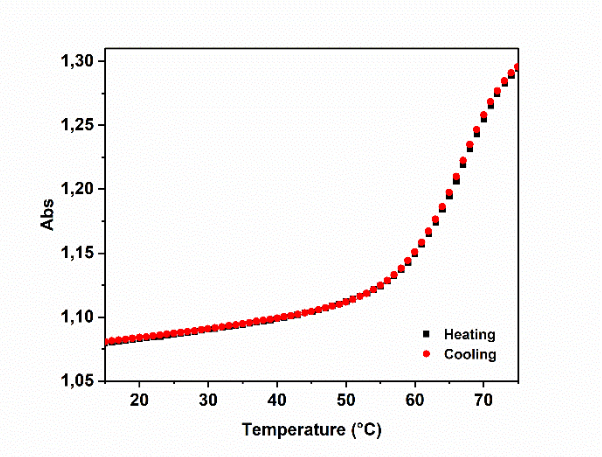

5’-d(TAG GTC AAT ACT) (ODN-**1**)
3’-d(ATC C2G TT3 TGA) (ODN-**9**)

5’-d(TAG GTC AAT ACT) (ODN-**1**)
3’-d(ATC C2G TT4 TGA) (ODN-**10**)

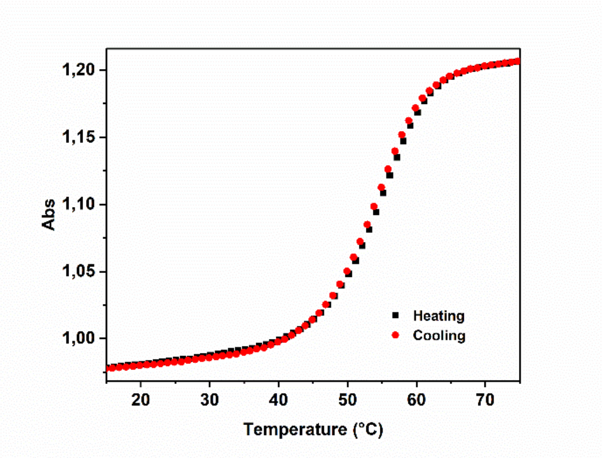

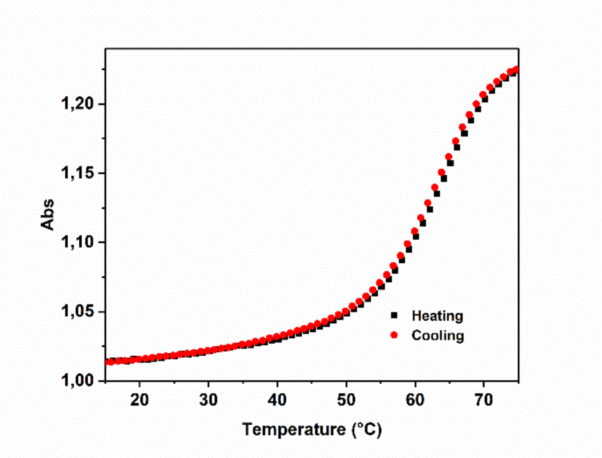

5’-d(TAG GTC AAT ACT) (ODN-**1**)
3’-d(ATC CAG TT3 TG2) (ODN-**11**)

5’-d(TAG GTC AAT ACT) (ODN-**1**)
3’-d(ATC CAG TT4 TG2) (ODN-**12**)

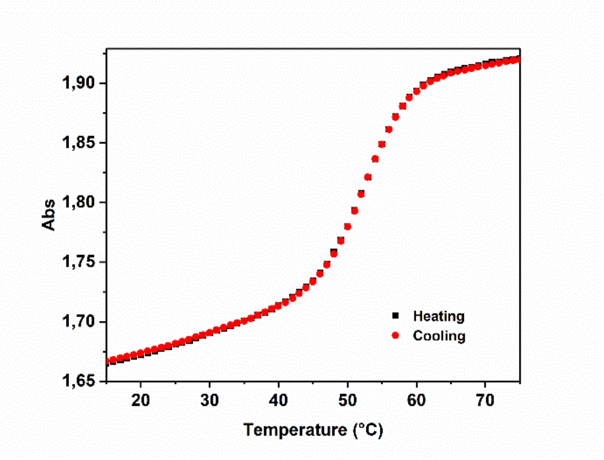

5’-d(TAG GTC AAT ACT) (ODN-**1**)
3’-d(ATC CAG TT1 TGA) (ODN-**3**)
3’-d(ATC CAG TTA TGA) (ODN-**2**)

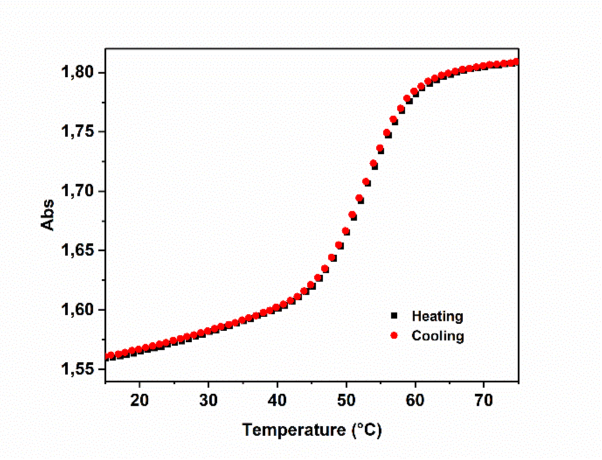

5’-d(TAG GTC AAT ACT) (ODN-**1**)
3’-d(ATC C1G TT1 TG1) (ODN-**4**)
3’-d(ATC CAG TTA TGA) (ODN-**2**)

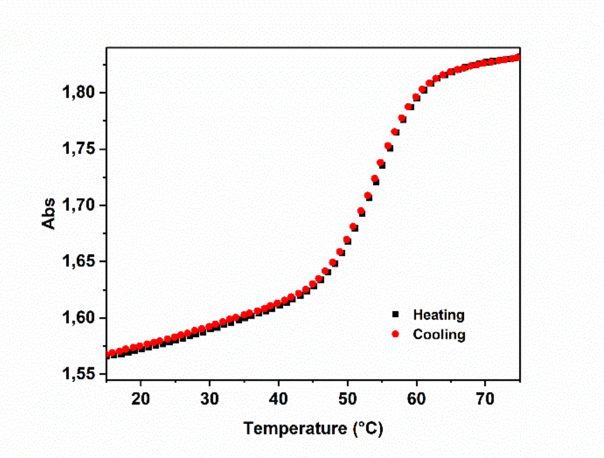

5’-d(TAG GTC AAT ACT) (ODN-**1**)
3’-d(ATC CAG TT2 TGA) (ODN-**5**)
3’-d(ATC CAG TTA TGA) (ODN-**2**)

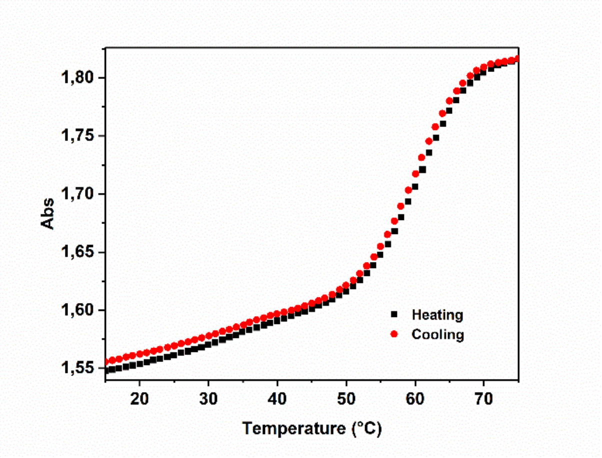

5’-d(TAG GTC AAT ACT) (ODN-**1**)
3’-d(ATC C2G TT2 TG2) (ODN-**6**)
3’-d(ATC CAG TTA TGA) (ODN-**2**)

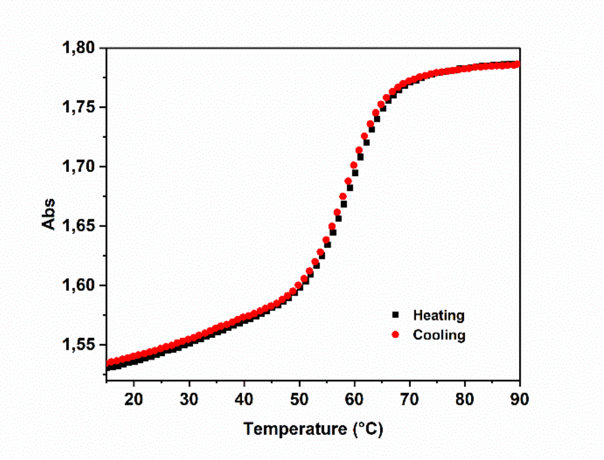

5’-d(TAG GTC AAT ACT) (ODN-**1**)
3’-d(ATC C2G TT3 TG2) (ODN-**7**)
3’-d(ATC CAG TTA TGA) (ODN-**2**)

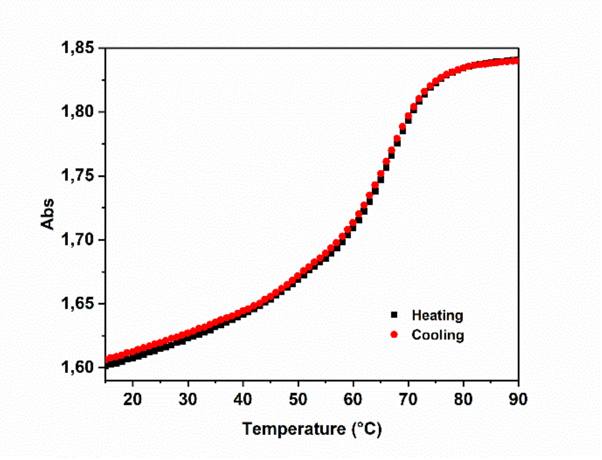

5’-d(TAG GTC AAT ACT) (ODN-**1**)
3’-d(ATC C2G TT4 TG2) (ODN-**8**)
3’-d(ATC CAG TTA TGA) (ODN-**2**)

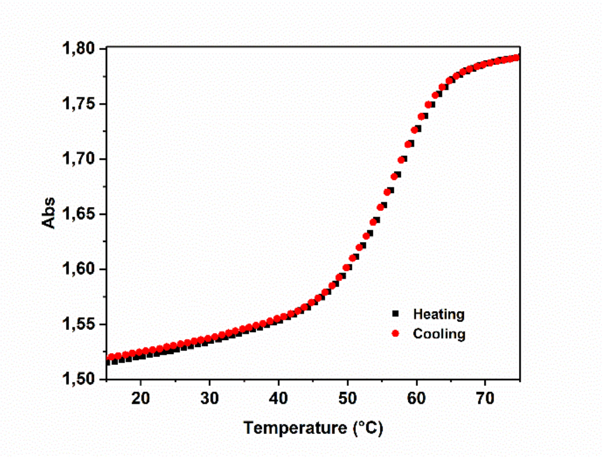

5’-d(TAG GTC AAT ACT) (ODN-**1**)
3’-d(ATC C2G TT3 TGA) (ODN-**9**)
3’-d(ATC CAG TTA TGA) (ODN-**2**)

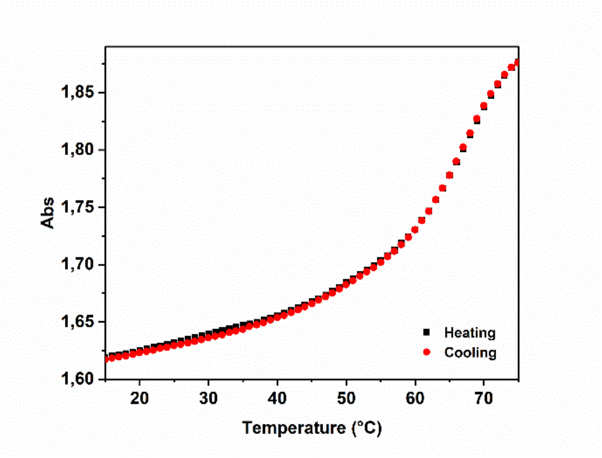

5’-d(TAG GTC AAT ACT) (ODN-**1**)
3’-d(ATC C2G TT4 TGA) (ODN-**10**)
3’-d(ATC CAG TTA TGA) (ODN-**2**)

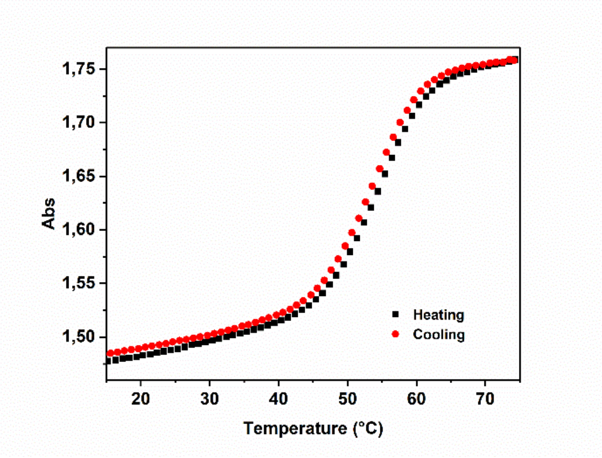

5’-d(TAG GTC AAT ACT) (ODN-**1**)
3’-d(ATC CAG TT3 TG2) (ODN-**11**)
3’-d(ATC CAG TTA TGA) (ODN-**2**)

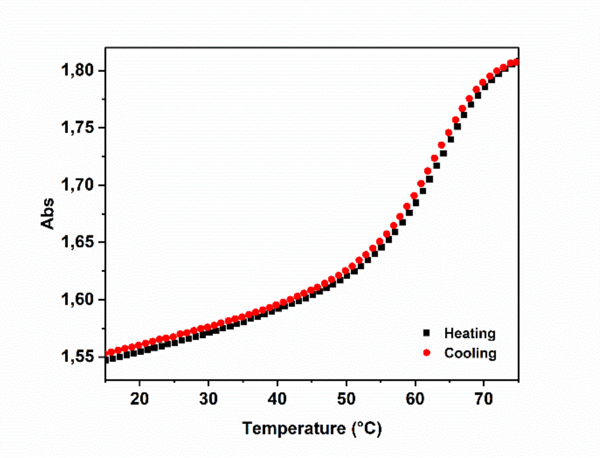

5’-d(TAG GTC AAT ACT) (ODN-**1**)
3’-d(ATC CAG TT4 TG2) (ODN-**12**)
3’-d(ATC CAG TTA TGA) (ODN-**2**)

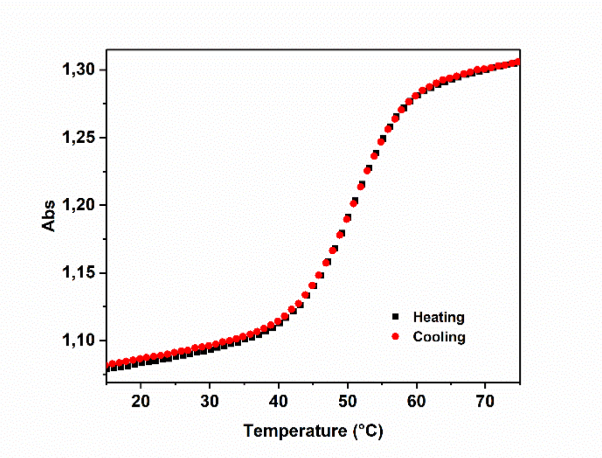

5’-d(TAG GTC AAT ACT) (ODN-**1**)
3’-d(ATC CAG TTA TGA5) (ODN-**13**)

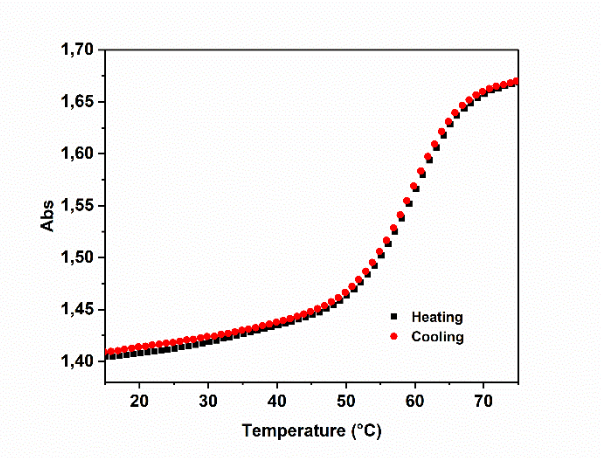

5’-d(TAG GTC AAT ACT) (ODN-**1**)
3’-d(ATC C2G TT2 TG2) (ODN-**6**)
3’-d(ATC CAG TTA TGA5) (ODN-**13**)

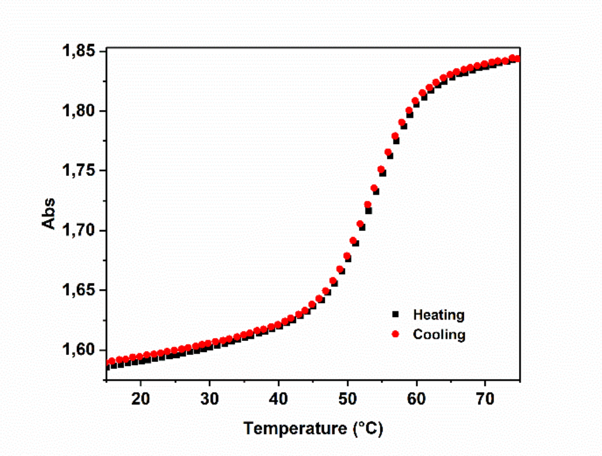

5’-d(TAG GTC AAT ACT) (ODN-**1**)
3’-d(ATC C**1**G TT**1** TG**1**) (ODN-**4**)
3’-d(ATC CAG TTA TGA**5**) (ODN-**13**)

Figure S4. Thermal denaturation curves of oligonucleotide duplexes. All measurements were performed at 260 nm at a concentration of 5 µM + 5 µM single strand at a heating rate of 1.0 °C/min in 100 mM NaCl, 10 mM $MgCl_2$, and 10 mM Na-cacodylate (pH 7.0).

NMR Measurements

General: NMR spectra were measured at 599.74 MHz, 399.89 MHz or 300.15 MHz for ^{1}H, at 150.82 MHz, 100.56 MHz or 75.47 MHz for ^{13}C and at 121.5 MHz for ^{31}P. The *J* values are given in Hz; δ values in ppm relative to Me_4Si as internal standard. For NMR spectra recorded in [D_6]DMSO, the chemical shift of the solvent peak was set to 2.50 ppm for ^{1}H NMR and 39.50 ppm for ^{13}C NMR.

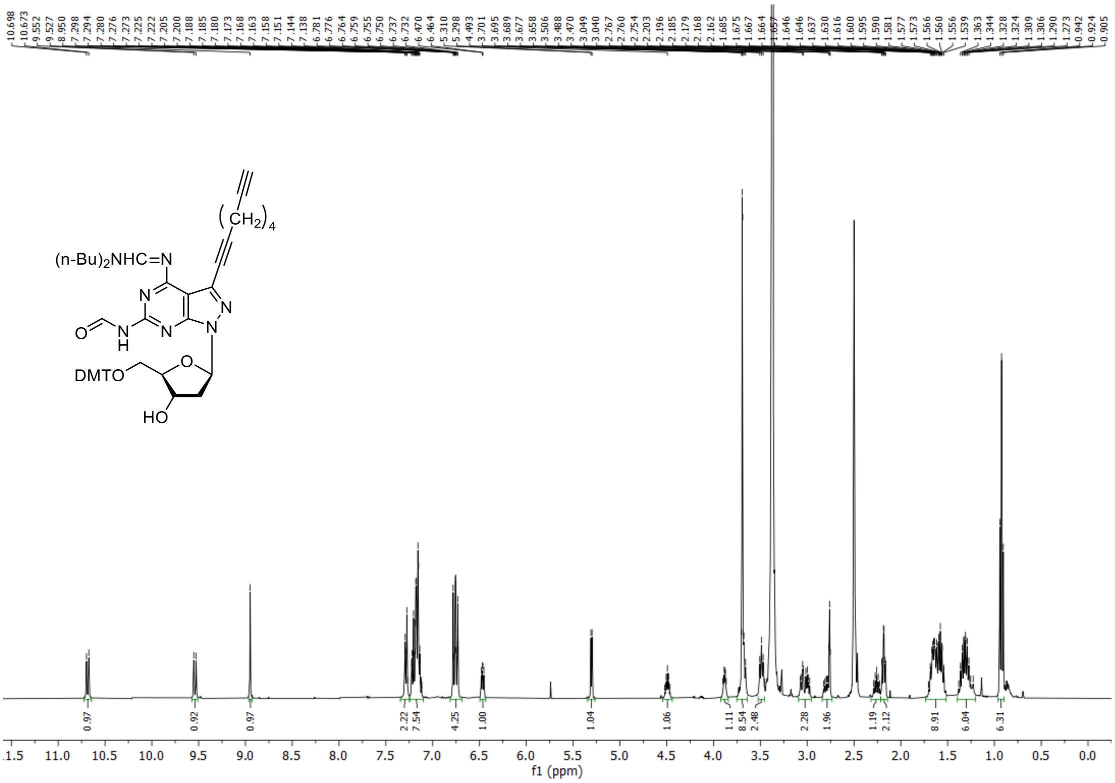

Figure S5. ^{1}H NMR spectrum of compound **7**

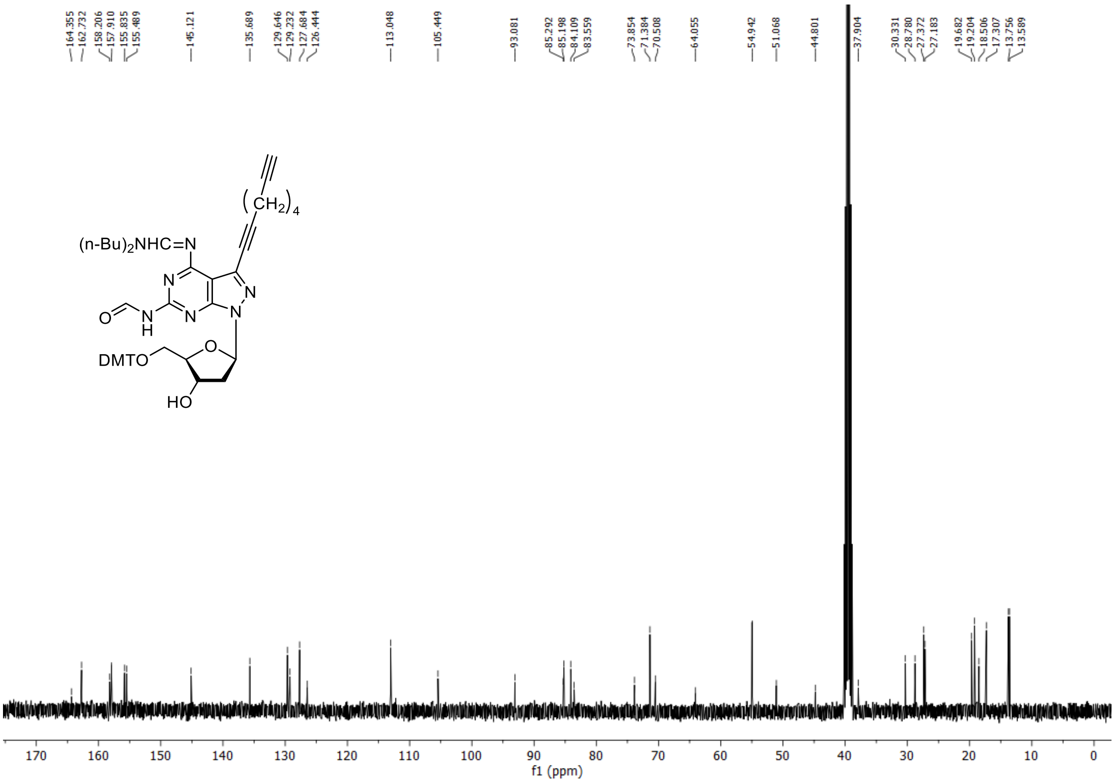

Figure S6. ^{13}C NMR spectrum of compound **7**

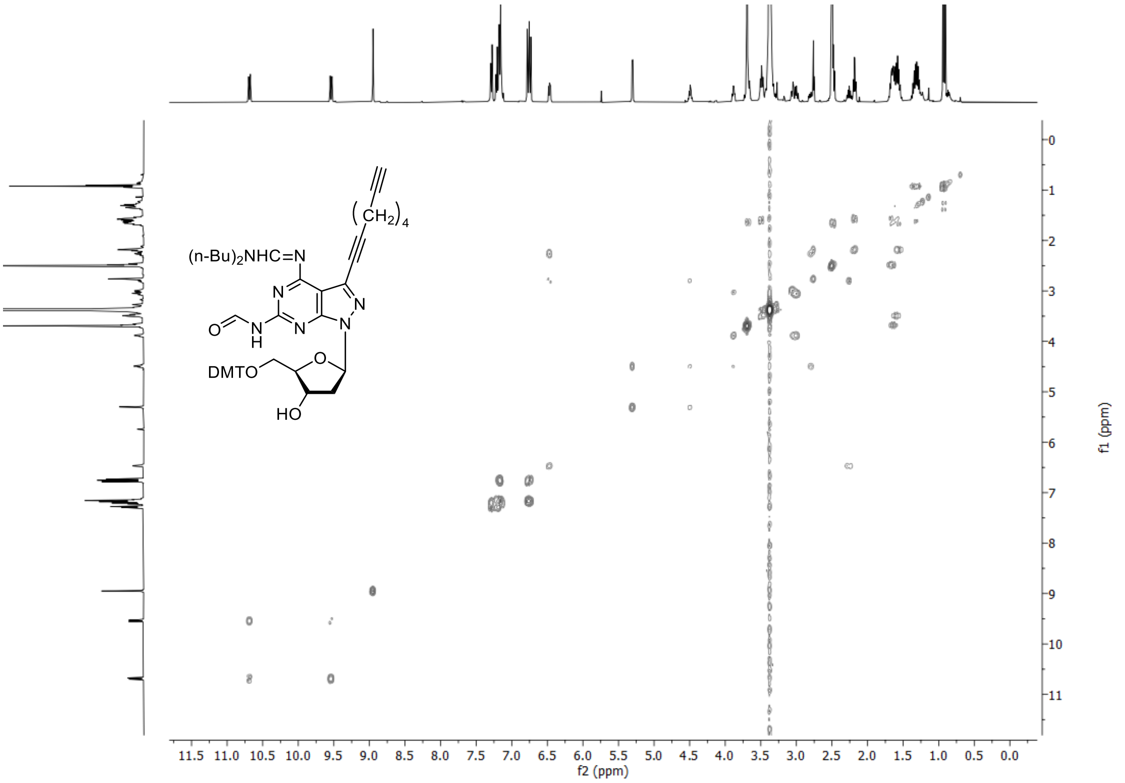

Figure S7. COSY spectrum of compound **7**

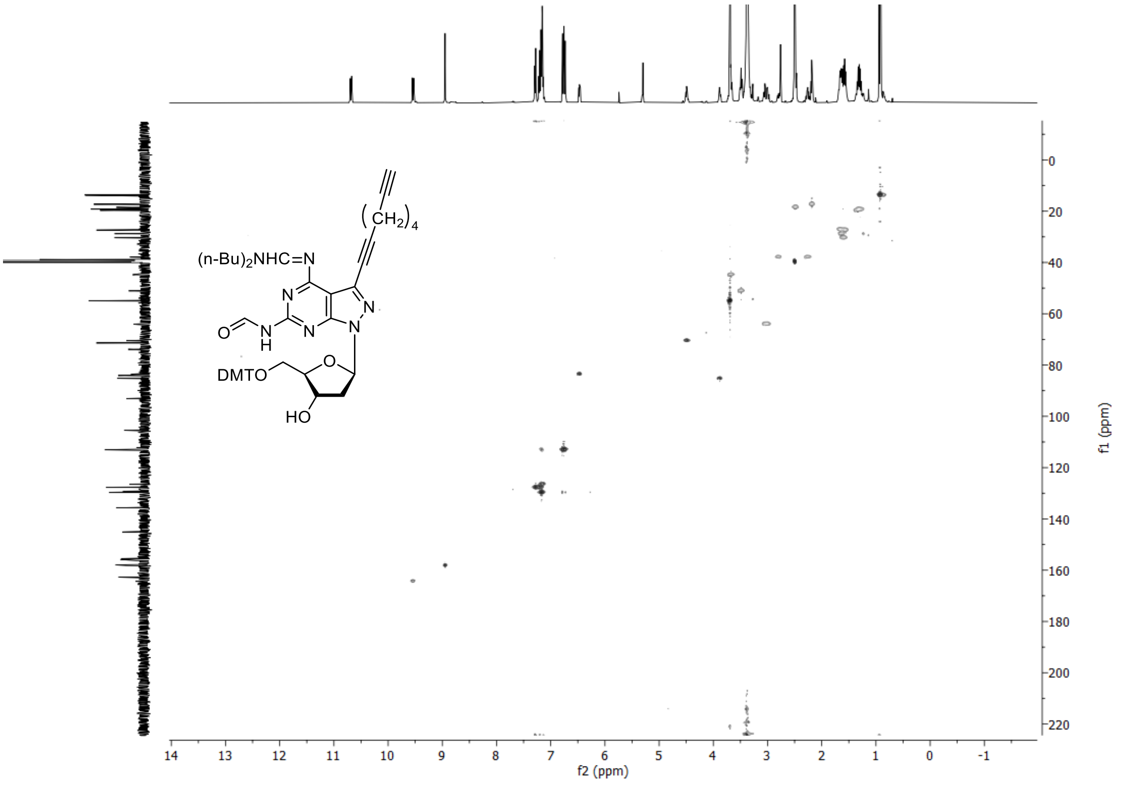

Figure S8. HSQC spectrum of compound **7**

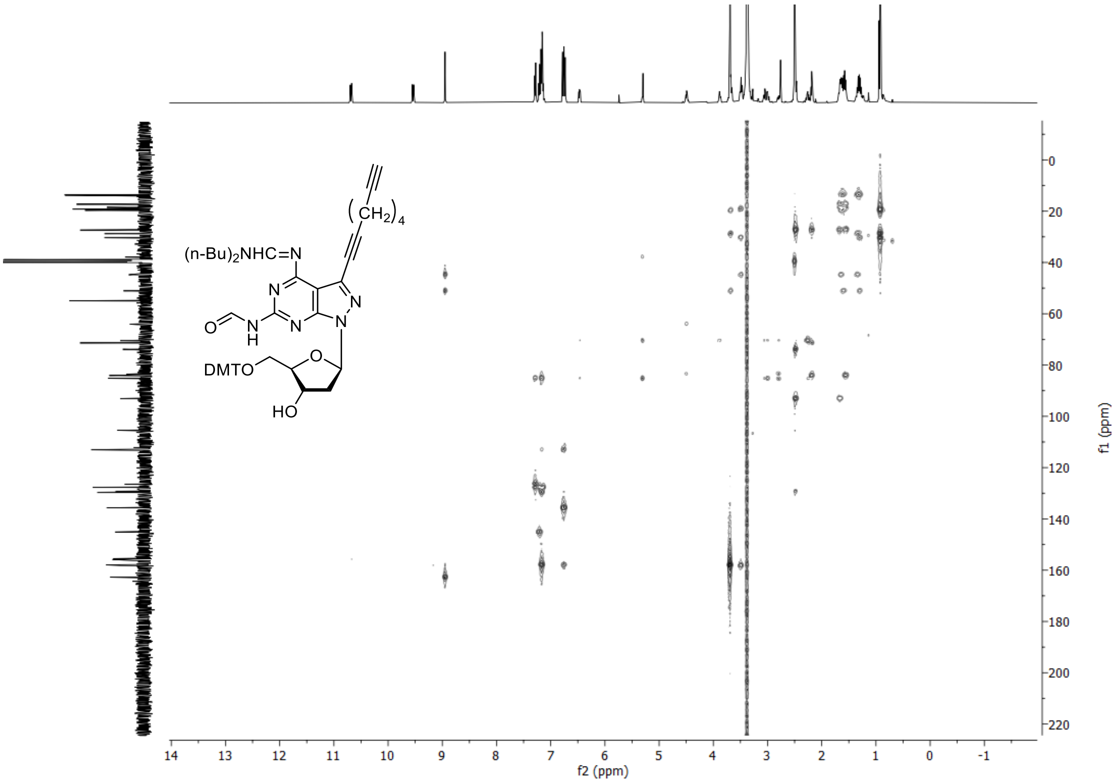

Figure S9. HMBC spectrum of compound **7**

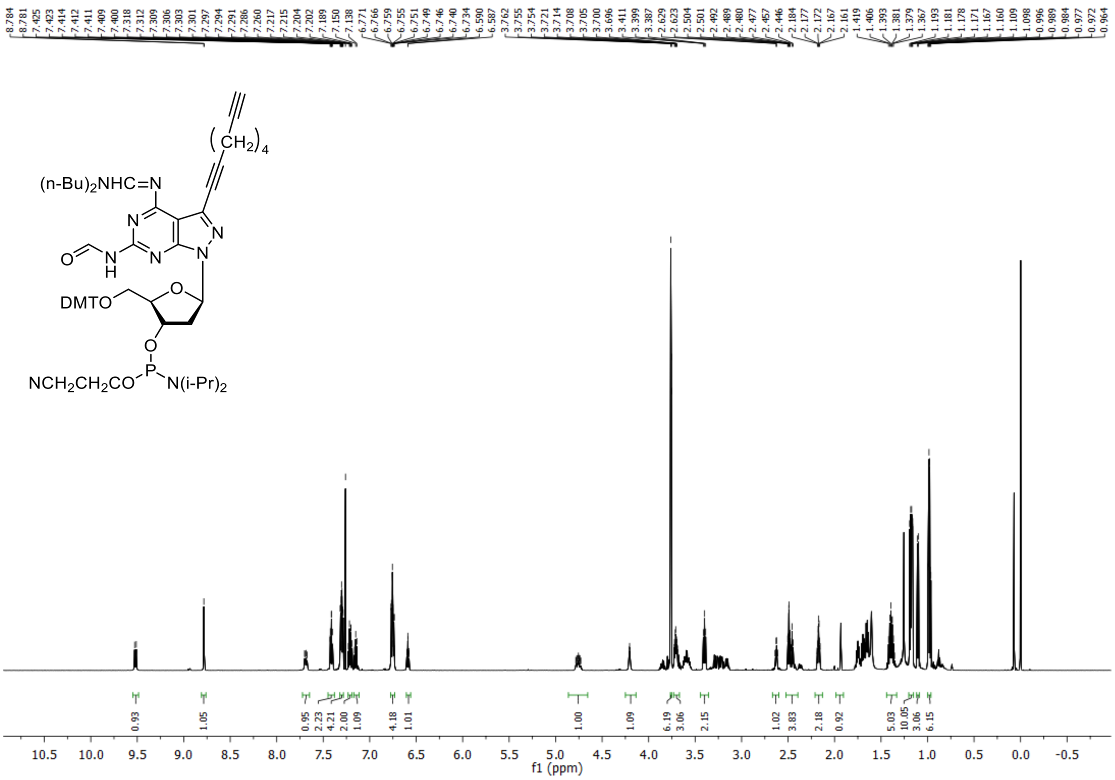

Figure S10. ^{1}H NMR spectrum of compound **8**

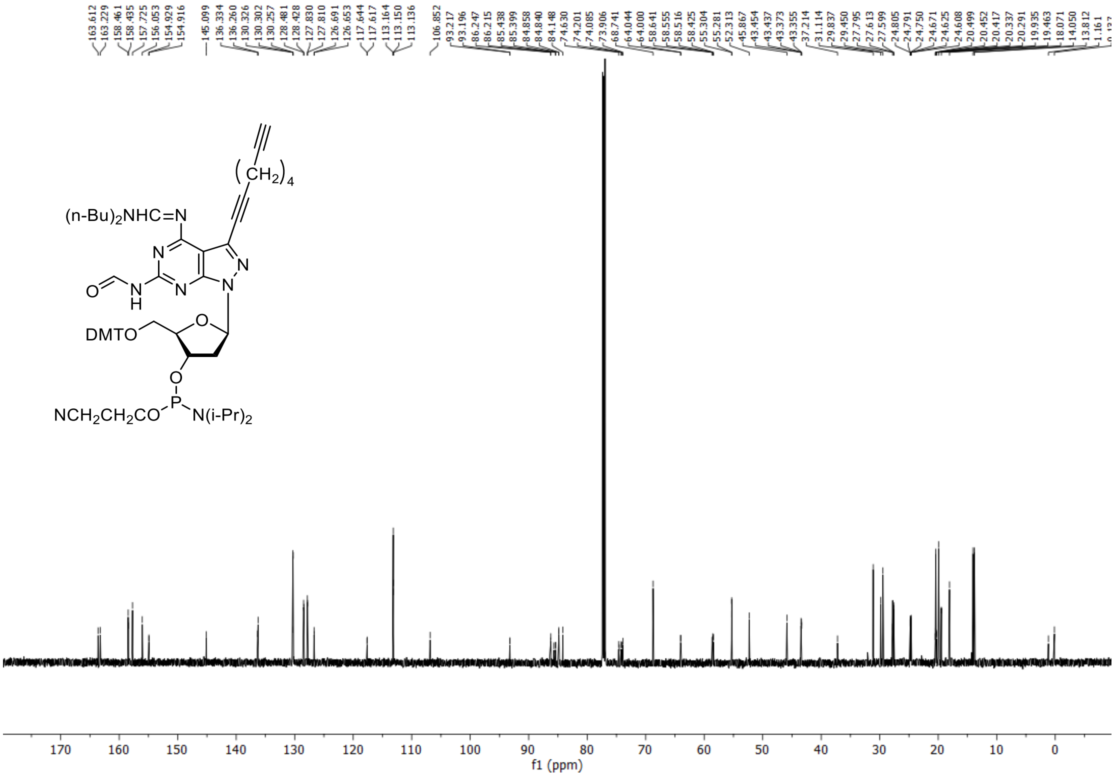

Figure S11. ^{13}C NMR spectrum of compound **8**

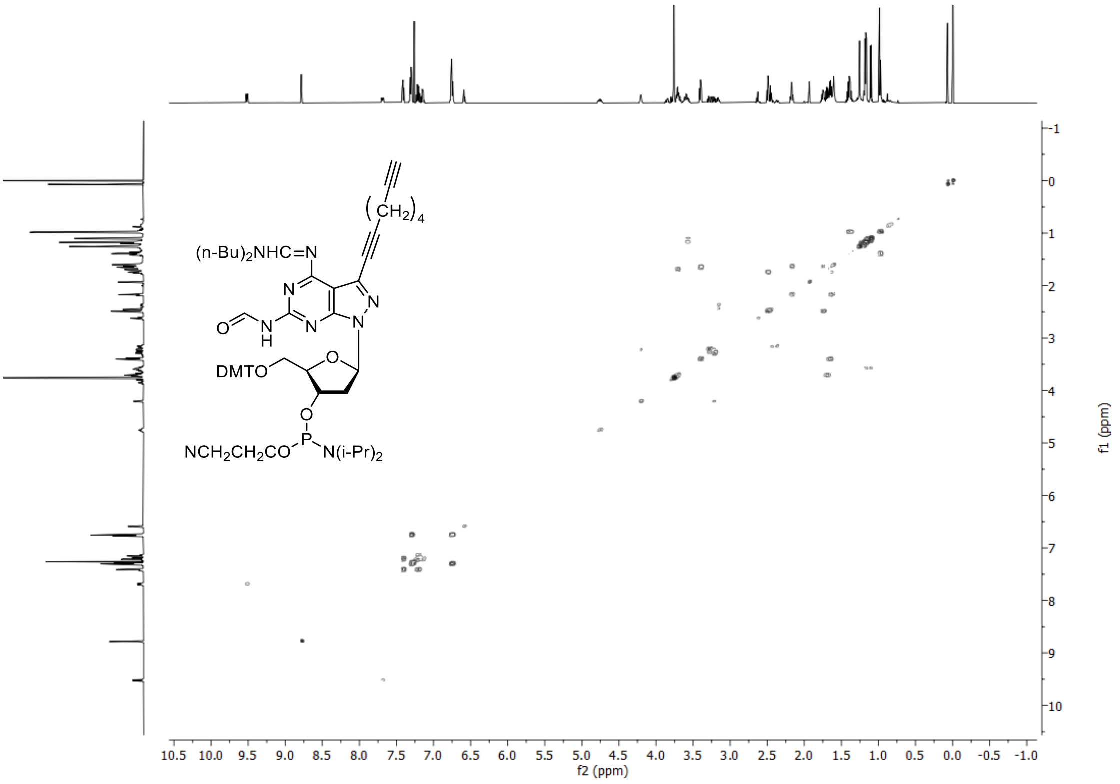

Figure S12. COSY spectrum of compound **8**

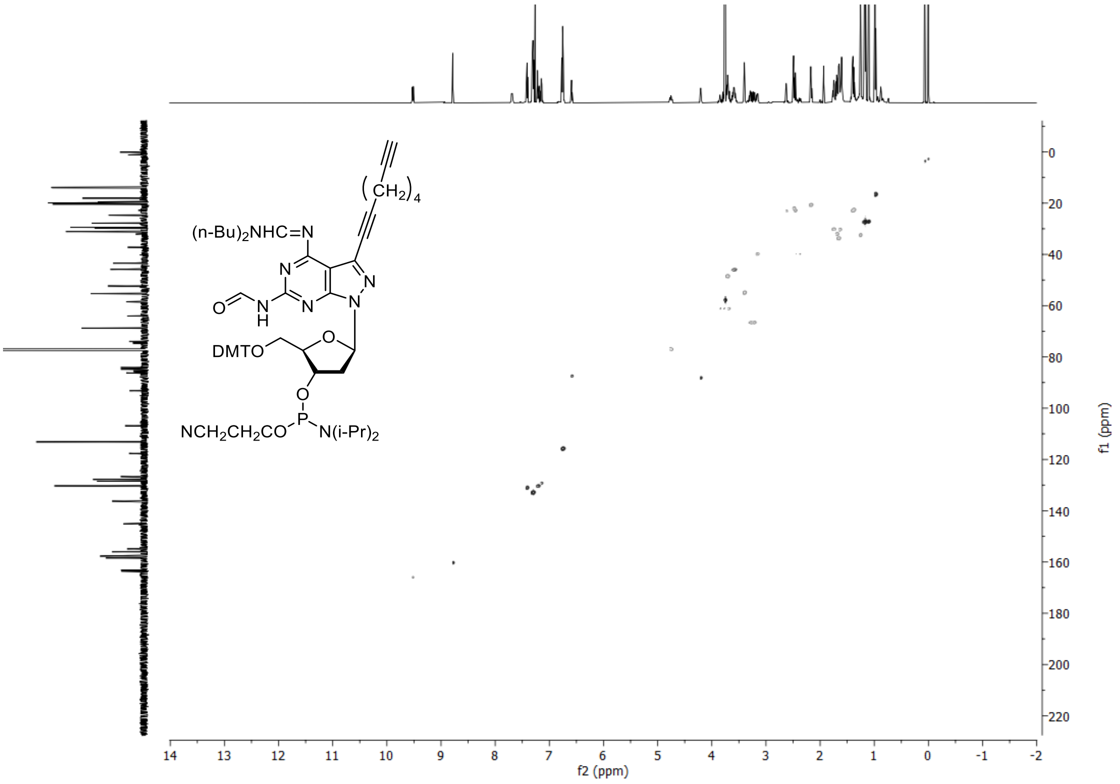

Figure S13. HSQC spectrum of compound **8**

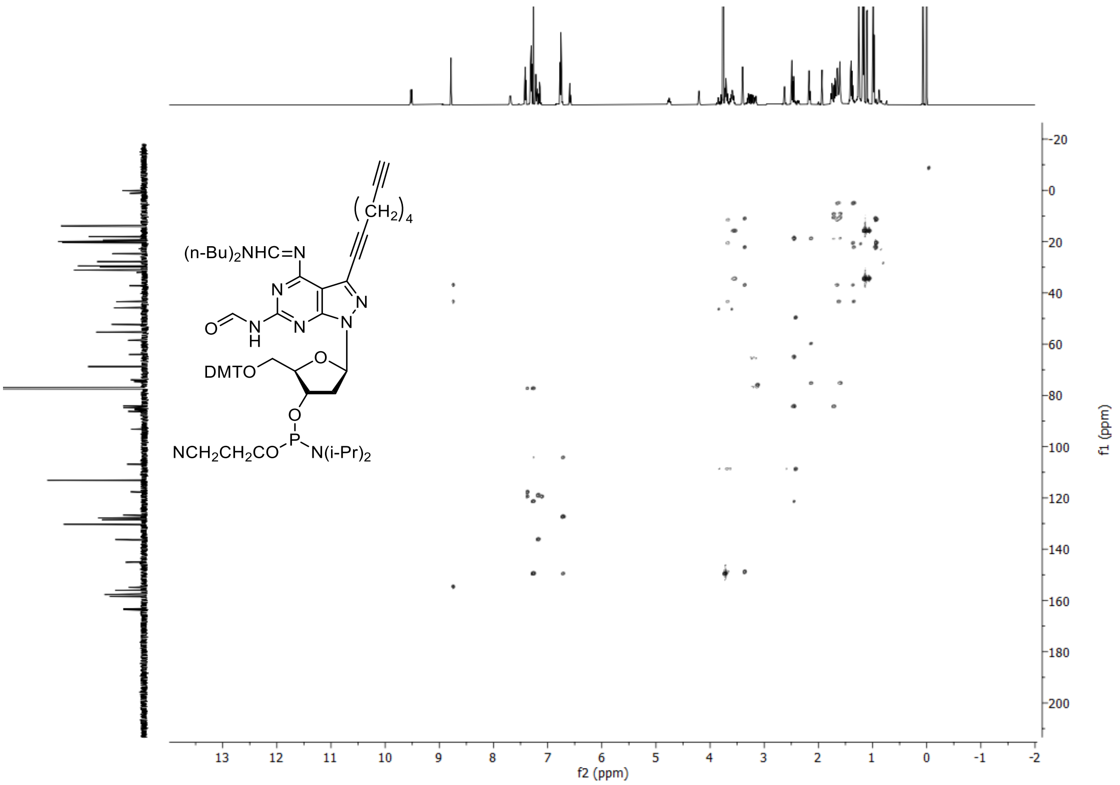

Figure S14. HMBC spectrum of compound **8**

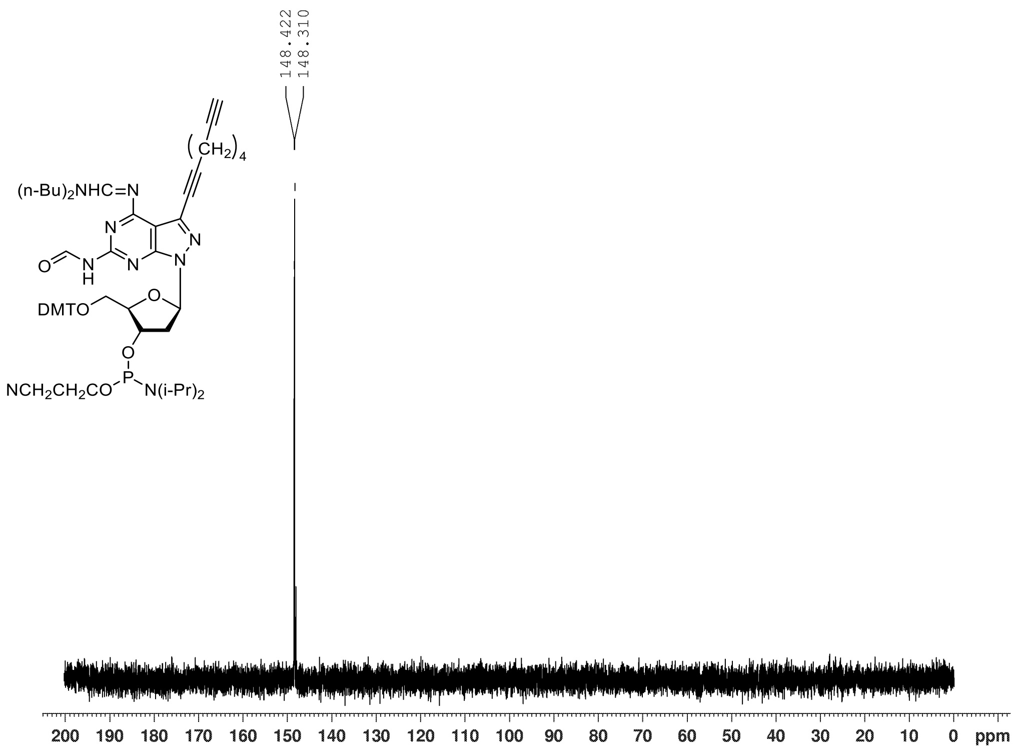

Figure S15. ^{31}P NMR spectrum of compound **8**

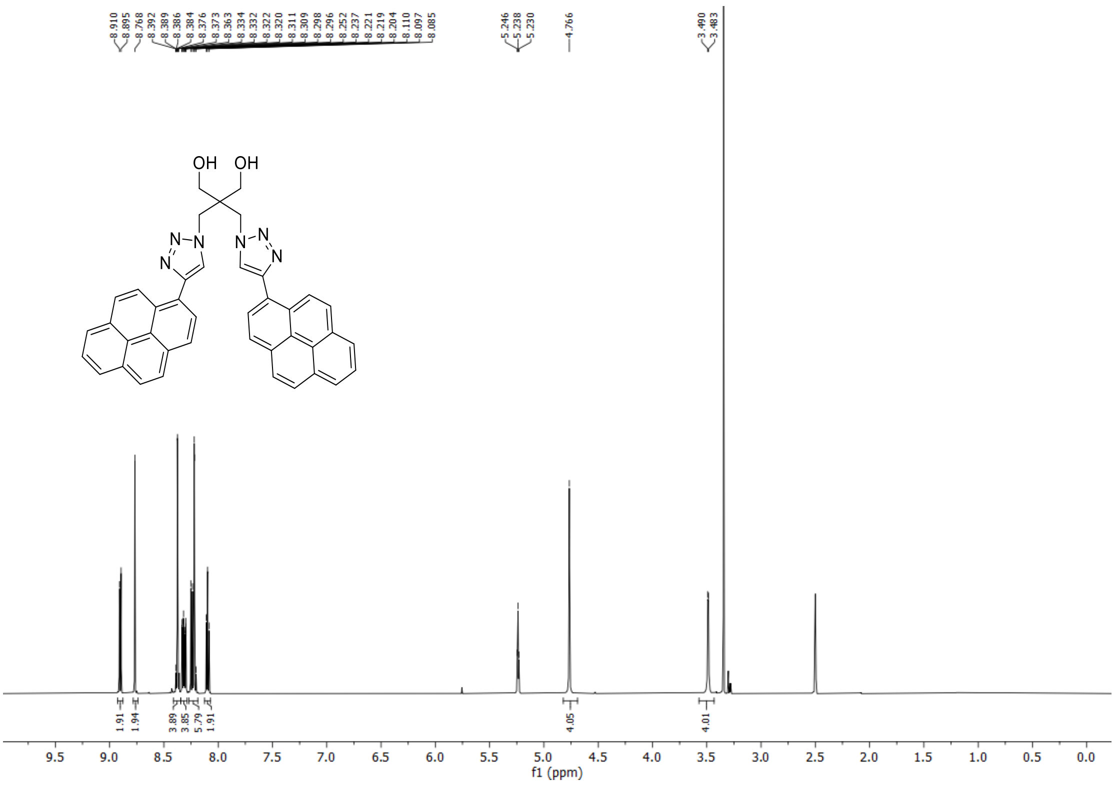

Figure S16. ^{1}H NMR spectrum of compound **5**

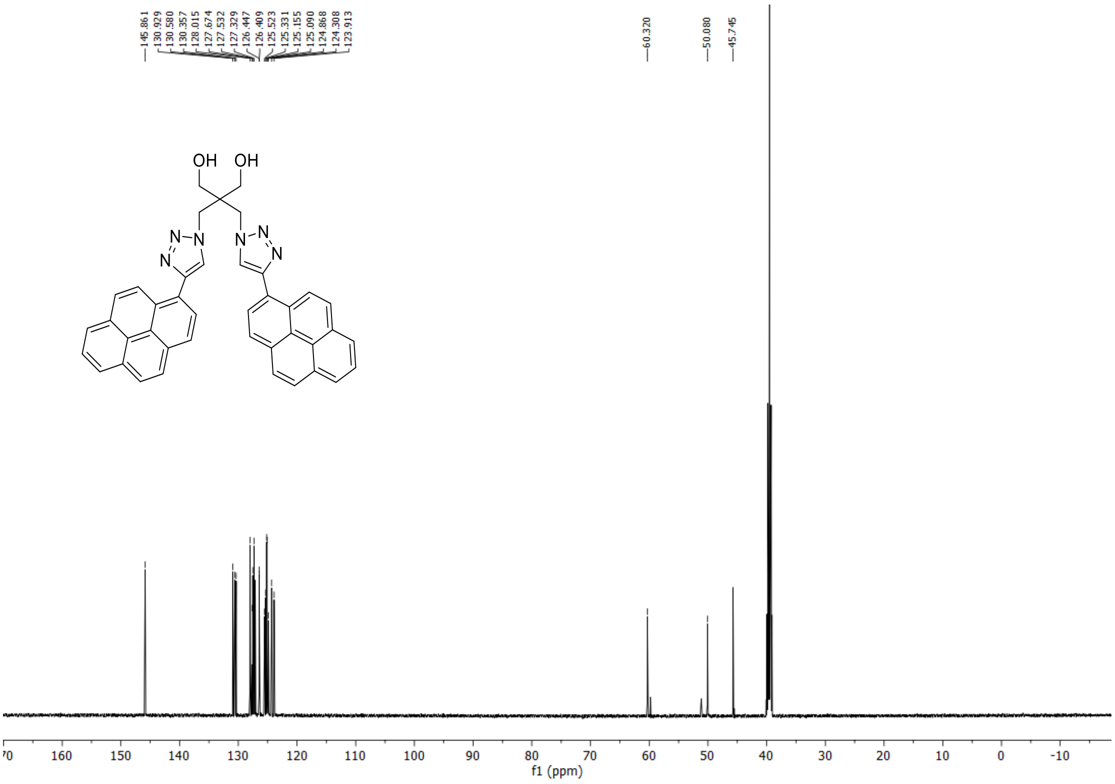

Figure S17. ^{13}C NMR spectrum of compound **5**

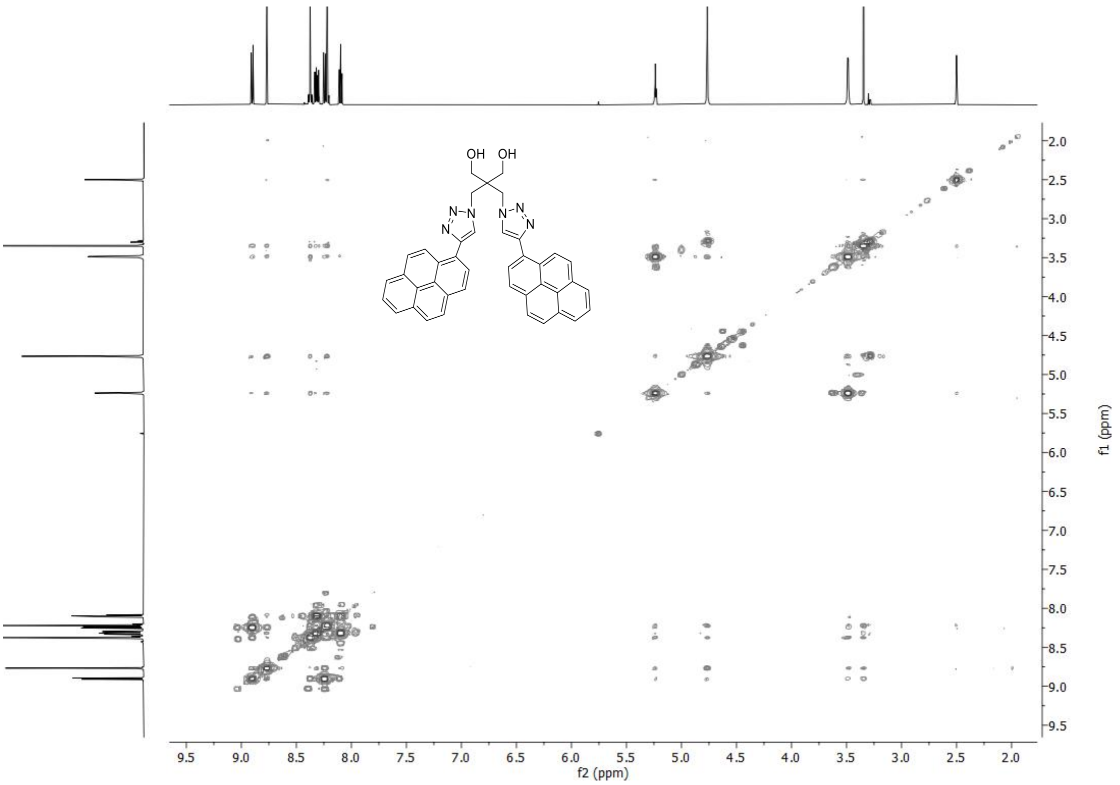

Figure S18. COSY spectrum of compound **5**

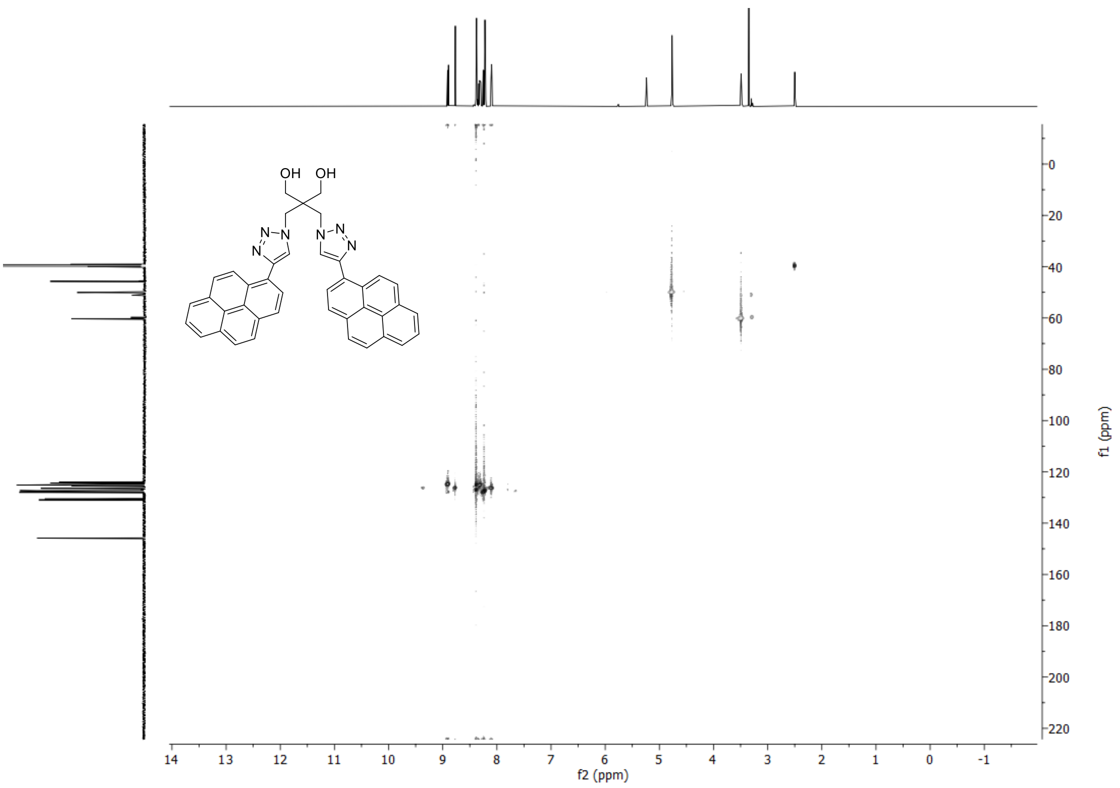

Figure S19. HSQC spectrum of compound **5**

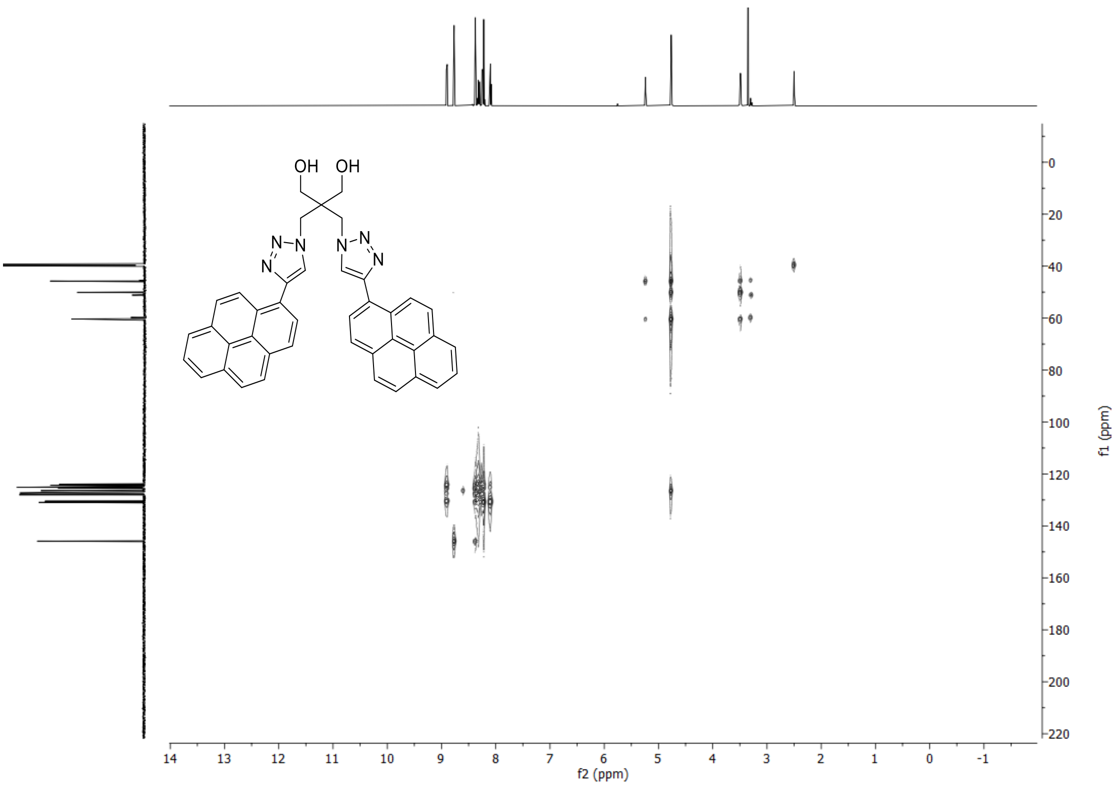

Figure S20. HMBC spectrum of compound **5**

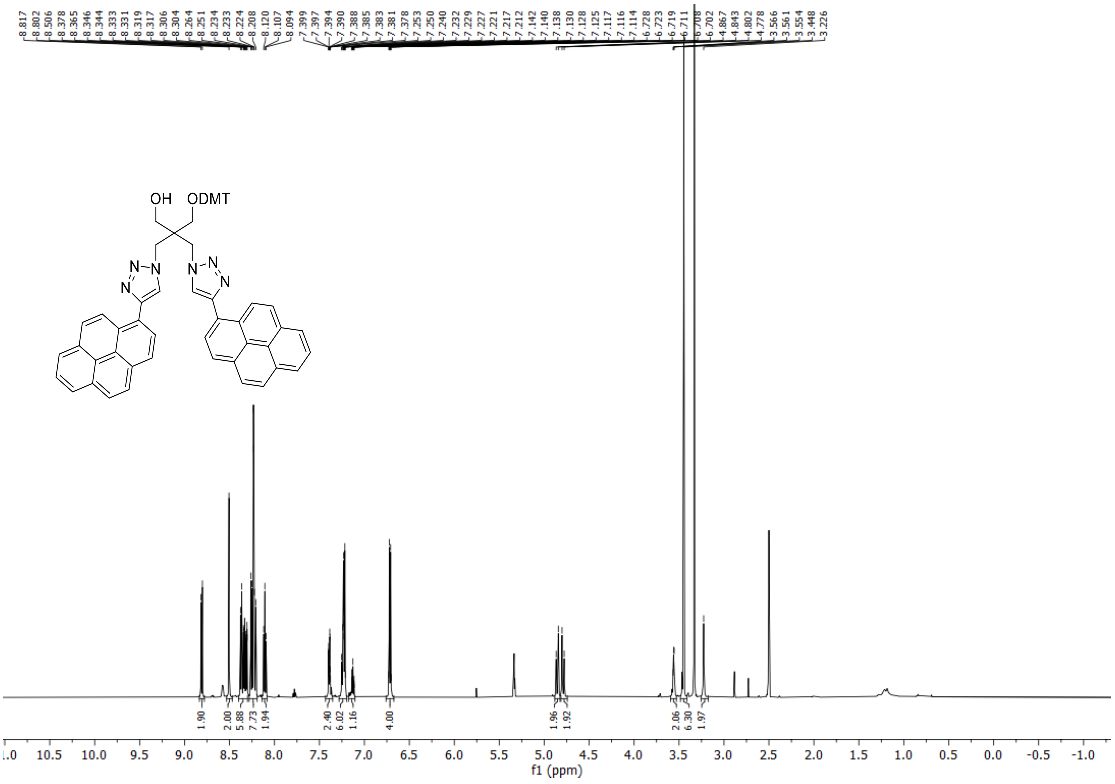

Figure S21. ^{1}H NMR spectrum of compound **14**

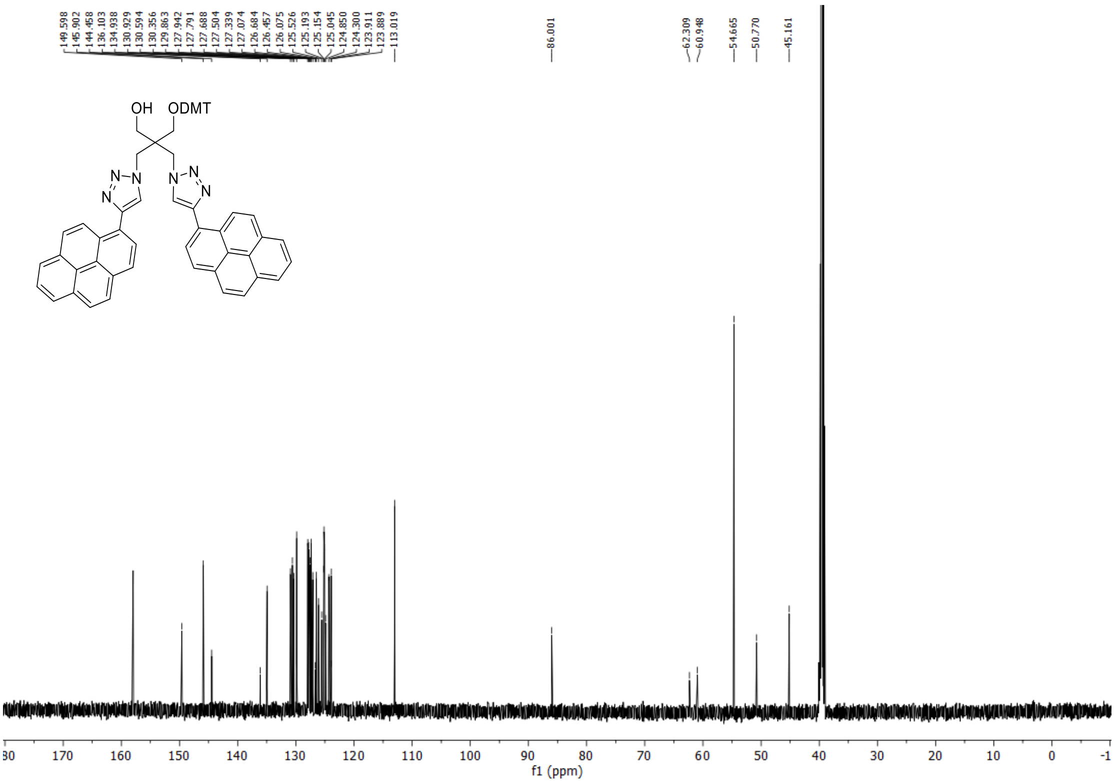

Figure S22. ^{13}C NMR spectrum of compound **14**

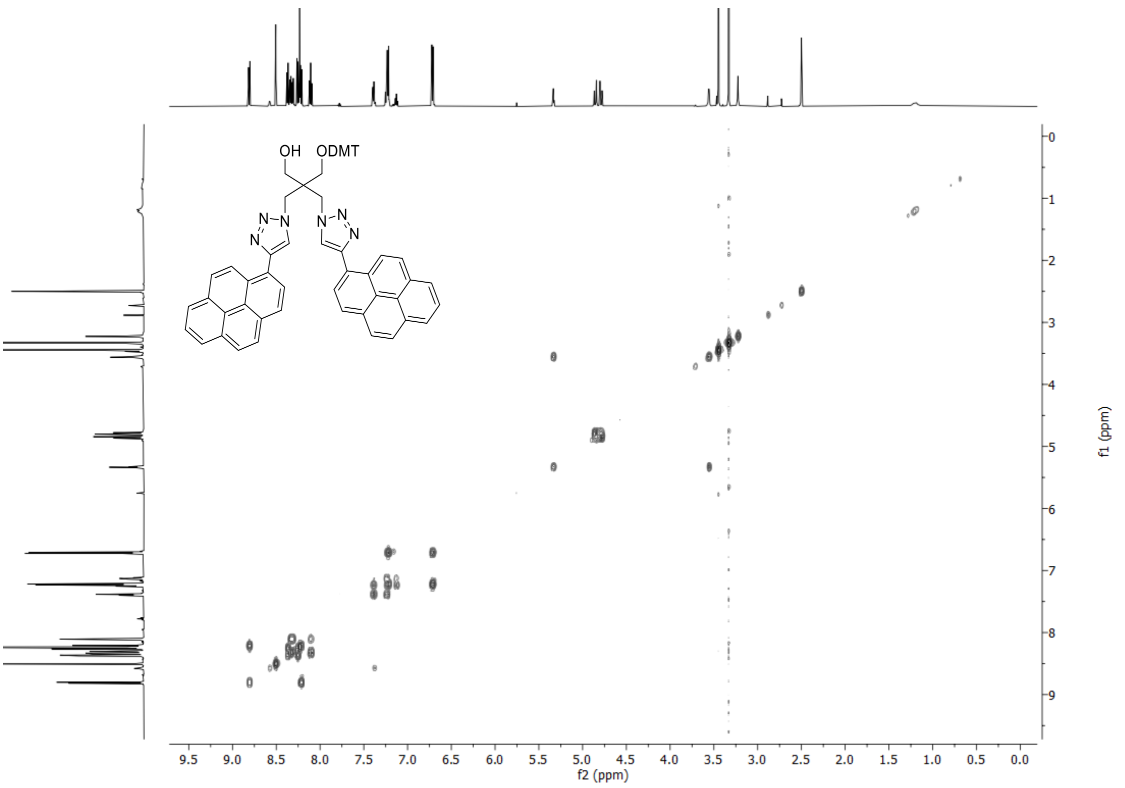

Figure S23. COSY spectrum of compound **14**

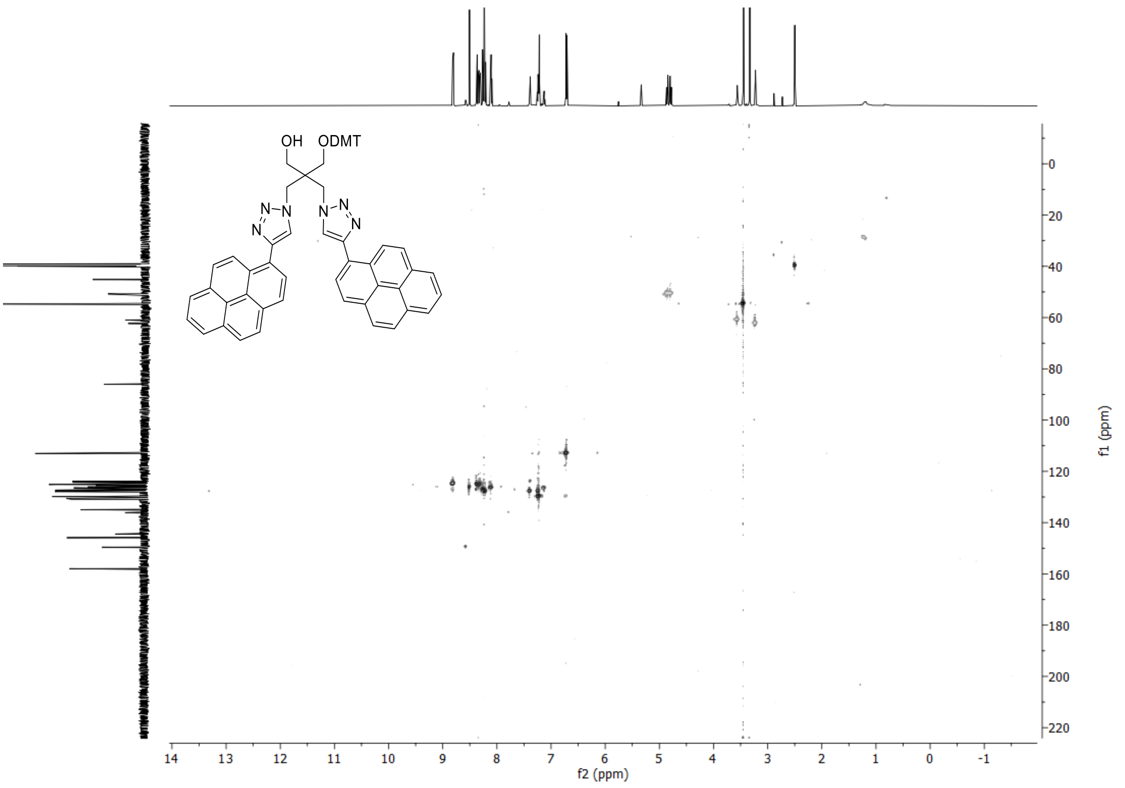

Figure S24. HSQC spectrum of compound **14**

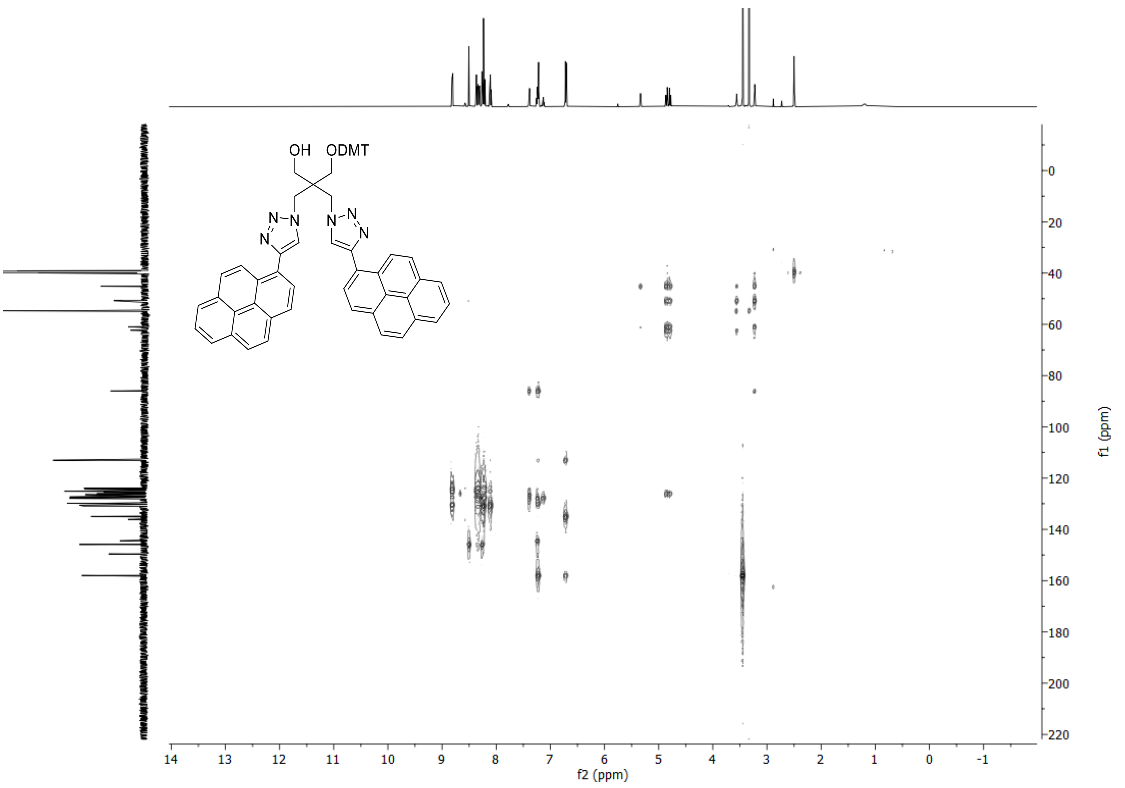

Figure S25. HMBC spectrum of compound **14**

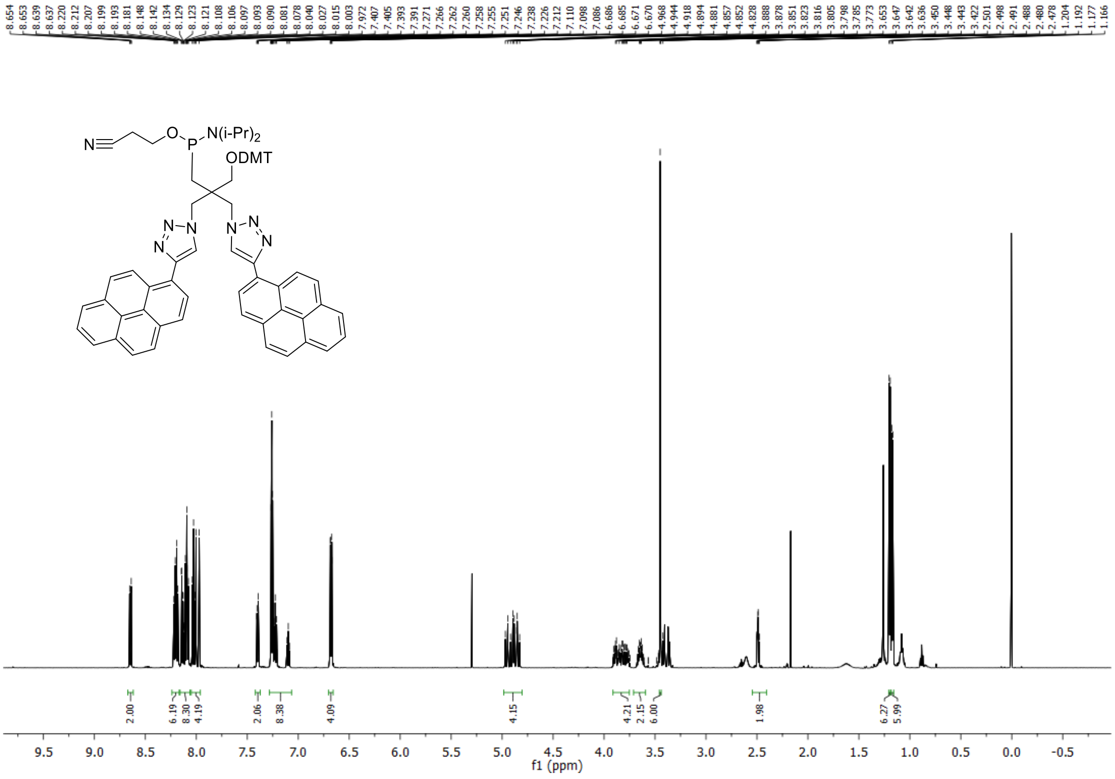

Figure S26. ^{1}H NMR spectrum of compound **15**

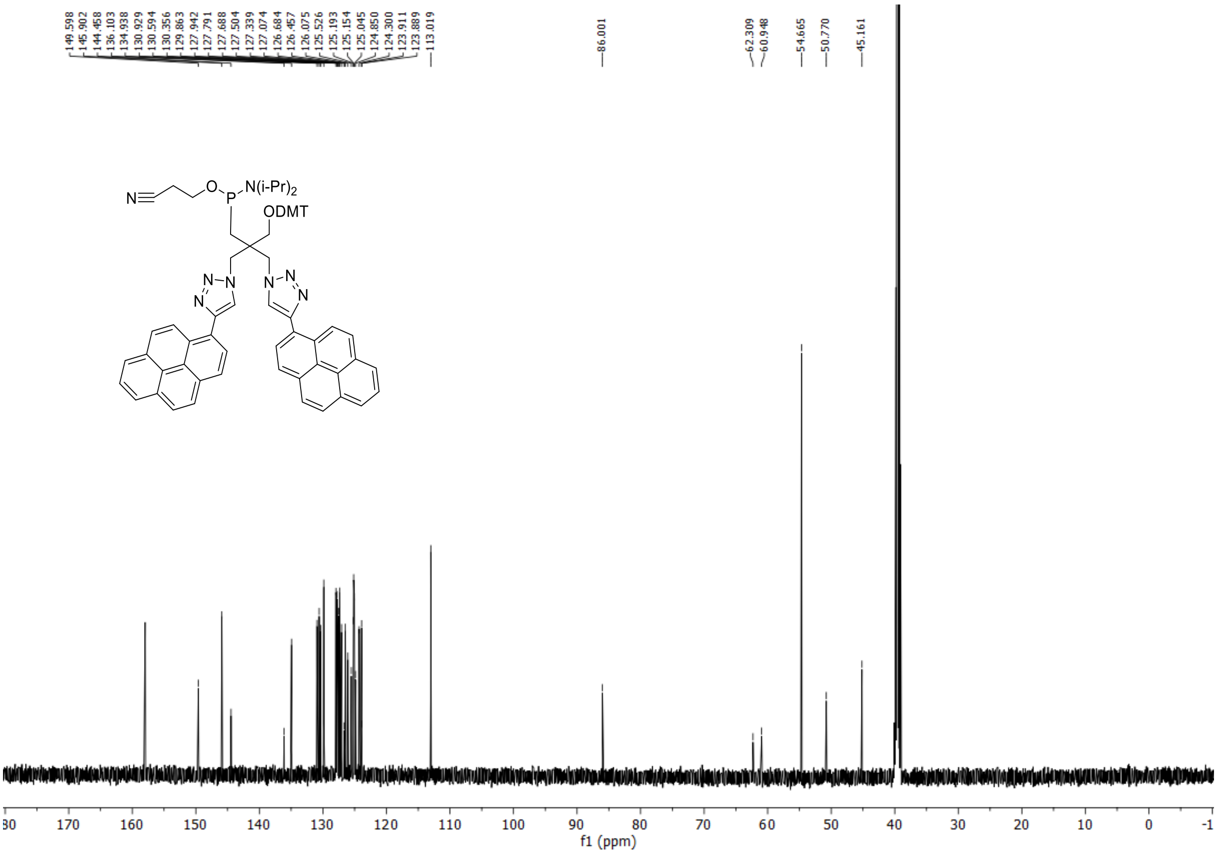

Figure S27. ^{13}C NMR spectrum of compound **15**

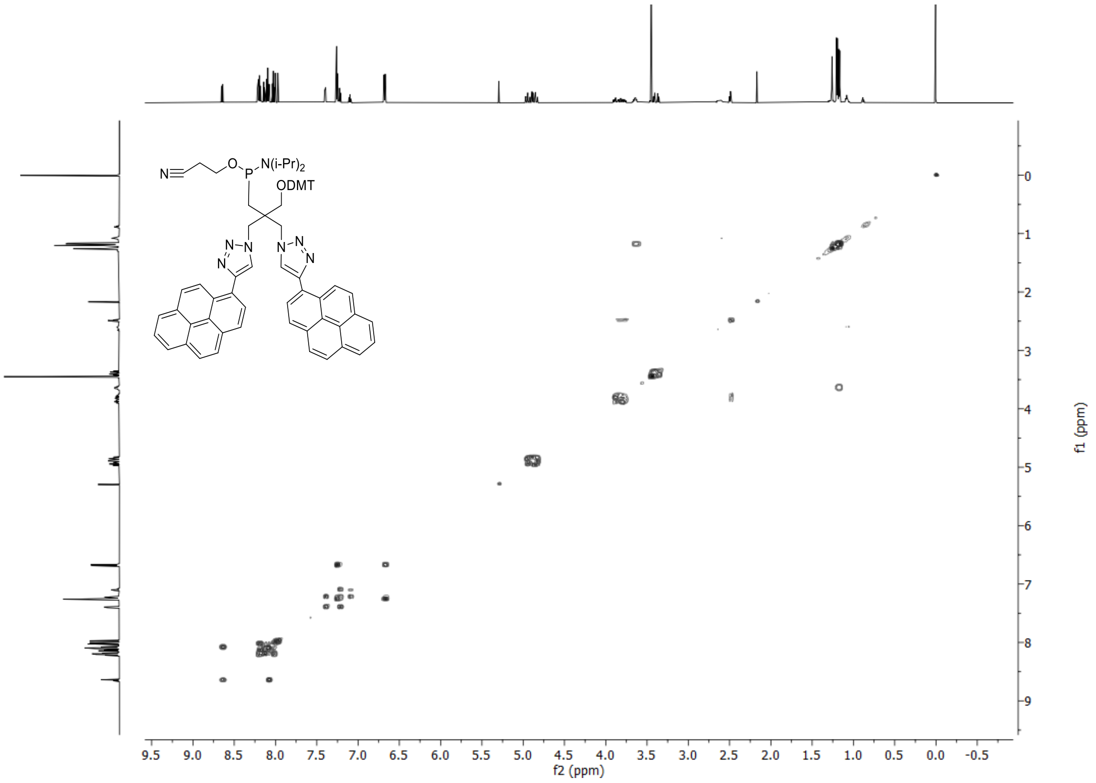

Figure S28. COSY spectrum of compound **15**

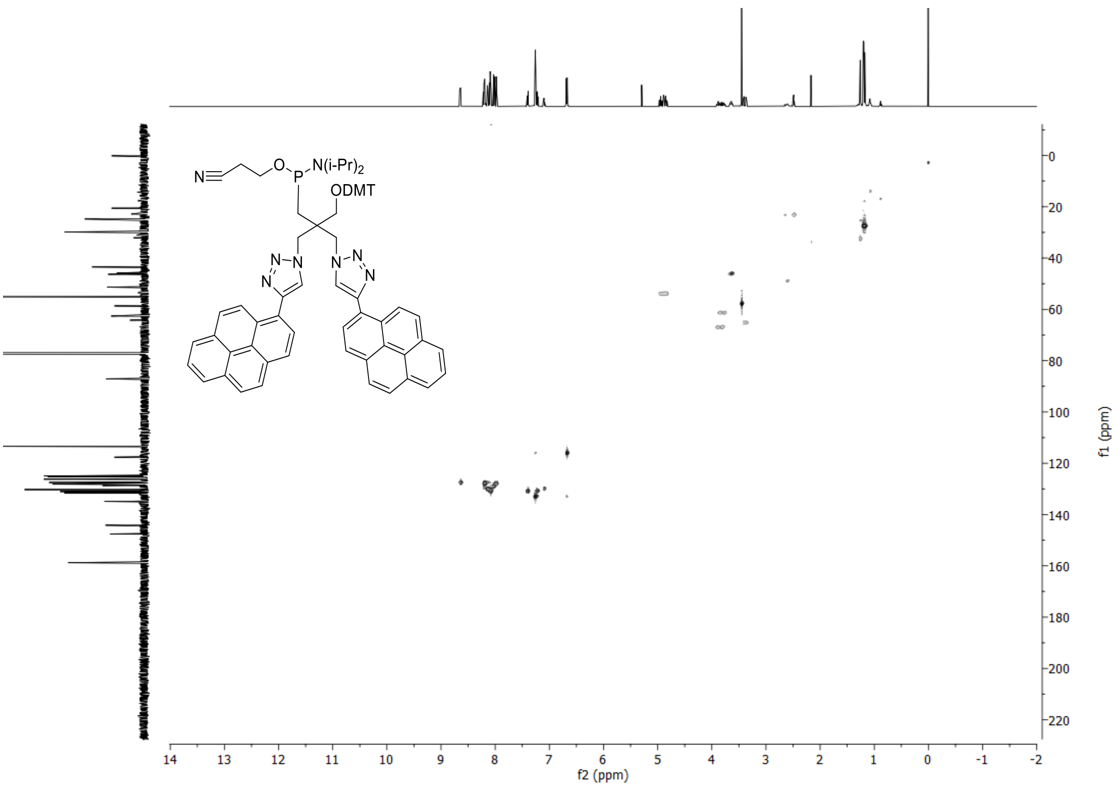

Figure S29. HSQC spectrum of compound **15**

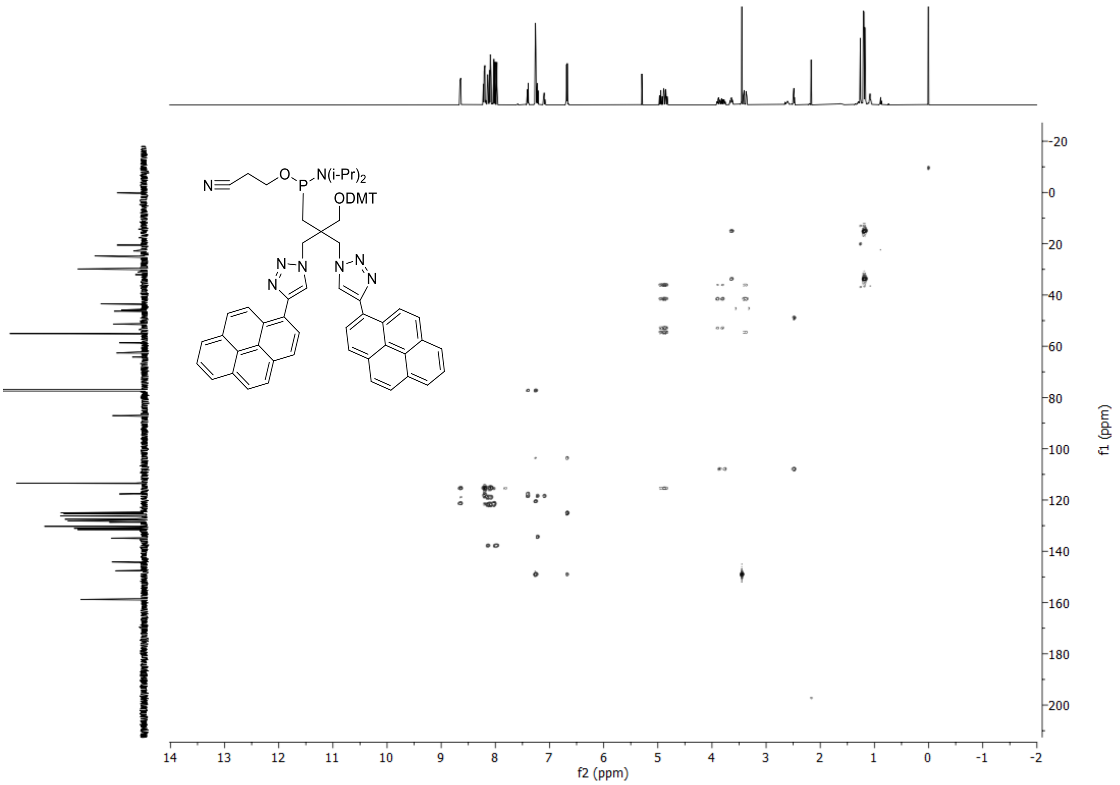

Figure S30. HMBC spectrum of compound **15**

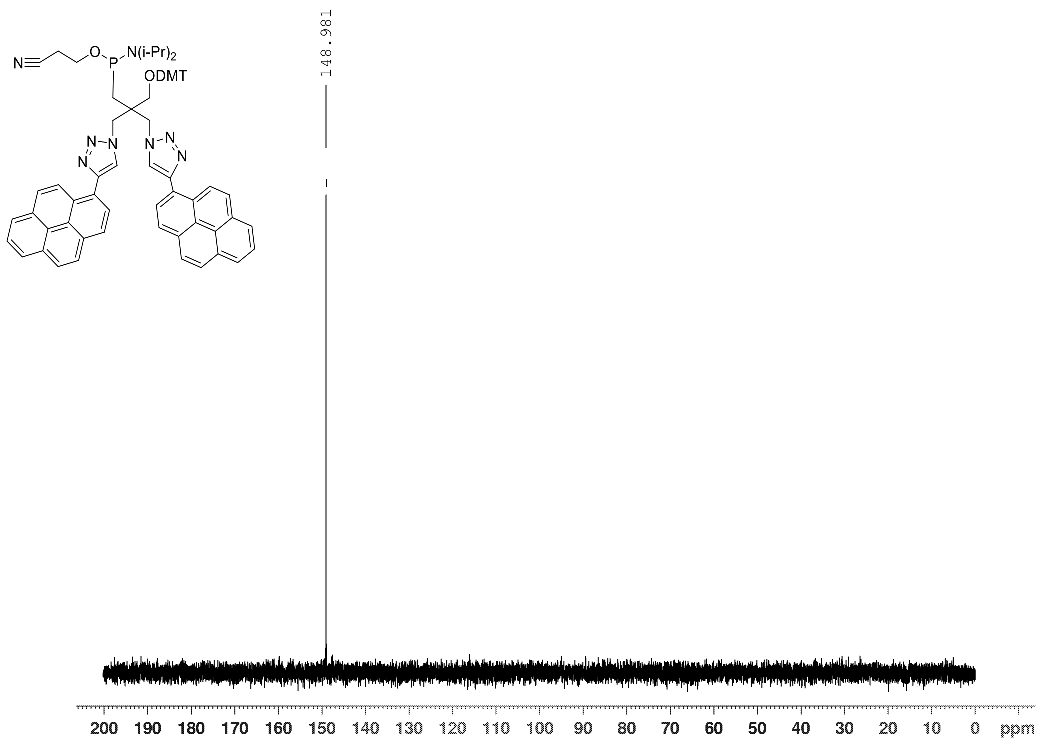

Figure S31. ^{31}P NMR spectrum of compound **15**
